# Breaking dependence on melanisation imparts diversity to a dogmatic invasion strategy of phytopathogenic fungi

**DOI:** 10.1038/s41467-026-74937-6

**Published:** 2026-06-27

**Authors:** Takeru Ohzawa, Ayaka Tani, Kazuho Takesue, Takehiro Kudo, Yunoka Akaba, Toko Yagisawa, Fukunosuke Arao, Koki Kume, Hiroki Irieda

**Affiliations:** 1 Graduate School of Science and Technology, Shinshu University, Nagano, 399-4598 Japan; 2 Faculty of Agriculture, Shinshu University, Nagano, 399-4598 Japan; 3 Academic Assembly, Institute of Agriculture, Shinshu University, Nagano, 399-4598 Japan

**Keywords:** Pathogens, Microbe, Fungi

## Abstract

*Colletotrichum* and *Pyricularia* are destructive fungal phytopathogens that typically invade plants by forming a heavily melanised, unicellular infection structure known as an appressorium, enabling melanised appressorium-mediated entry (MAE). Appressorial melanisation is essential for invasion by these phytopathogens. Here, we report *Colletotrichum* fungi that can invade plants via nonmelanised appressorium-mediated entry (NMAE). Multifaceted analyses revealed interspecific variation in the degree of melanisation dependence across appressorial functions. NMAE-type appressoria accomplished these functions regardless of melanisation, thus being capable of early invasion. Comprehensive screening identified a monophyletic NMAE-type *Colletotrichum* group with rapid expansion. Critically, we identified an eccentric NMAE-type strain with erratic appressorial melanisation. We also rediscovered appressorium-independent hyphal tip-based entry (HTE)-type strains. Collectively, these findings establish a key paradigm for the plant infection strategy of *Colletotrichum* fungi. In contrast to the widely accepted view based on the MAE of *Colletotrichum*, this study suggests that appressorial melanisation is not universally required, underscoring the need to consider the emergence of NMAE- and HTE-type pathogens.

## Abstract (web summary)

Melanised appressorium-dependent invasion of phytopathogenic anthracnose and rice blast fungi is a longstanding dogma in plant pathology. Here the authors report melanisation- and/or appressorium-independent invasion strategies of anthracnose fungi.

## Introduction

Various groups of fungi have evolved specialised infection structures known as appressoria, enabling them to adhere to and breach host surfaces^1^. The anthracnose fungi *Colletotrichum* species and the rice blast fungus *Pyricularia oryzae*, which are among the top 10 most destructive fungal plant pathogens globally^2^, conventionally develop a dome-shaped, heavily melanised, unicellular appressorium to invade plants via melanised appressorium-mediated entry (MAE)^1,3,4^. Therefore, appressorial melanisation is essential for successful MAE-based invasion by these fungi^5,6^. Several types of commercial melanin biosynthesis inhibitors (MBIs), including MBI-Ds, MBI-Rs, and MBI-P, that target melanin biosynthesis enzymes SCD1, THR1, and PKS1, respectively, have been used as effective agrochemicals for decades, particularly against leaf blast caused by *P. oryzae* in paddy fields^7–9^ (Supplementary Fig. 1). Similar effects have been observed in *Colletotrichum* spp., and studies of their melanin biosynthesis have provided valuable insights into drug targets for controlling rice blast disease. MAE research is critical for mitigating agricultural losses caused by *Colletotrichum* spp., which possess the broadest host range and global distribution; there are over 3000 *Colletotrichum* species–host plant species occurrence records^10^ and many outbreaks have occurred^11^.

The melanin layer forms within the appressorial cell wall and contributes to multiple structural and physiological functions (Supplementary Fig. 2). First, it serves as a semipermeable barrier that retains osmolytes while allowing water influx, thereby generating high turgor pressure for host penetration^12–14^; however, dihydroxyhexanoic acid (DHHA) polymers have recently been identified as genuine factors that reduce cell wall porosity for semipermeability^15^. Melanin pigmentation also directs the vertical emergence of the penetration peg at the host contact point, where the penetration pore is nonmelanised and surrounded by the toroidal F-actin network and septin ring which provide the cortical rigidity and membrane curvature necessary for penetration^16–19^. Furthermore, melanisation enhances cell wall rigidity protecting appressoria from fungal and plant-derived cell wall-degrading enzymes (CWDEs)^20,21^. Although previous studies have examined MBI-treated or melanin-deficient mutants of *Colletotrichum* and *Pyricularia*, none have described a fungus capable of plant invasion via a morphologically mature yet nonmelanised appressorium that induces necrotic lesion^16,17,20–30^. To date, crop infection through nonmelanised appressoria of *Colletotrichum* and *Pyricularia* has not been confirmed under agricultural conditions. A previously reported MBI-resistant *P. oryzae* strain carried a single mutation in SDH/SCD1, conferring reduced sensitivity to MBI-Ds alone^31–33^. The mulberry pathogen *Colletotrichum tropicale* (*Ctro*) S9275 exhibited MBI insensitiveness only in the presence of carbohydrates; however, appressorium development was blocked, and unconventional hyphal tip-based entry (HTE) into plant leaves occurred via the tips of elongated germ tube-like hyphae^34^. Moreover, under HTE-inducing conditions, an aseptate and nonmelanised appressorium-like structure at the tip of the germ tube-like hyphae was sometimes observed, where the successful entry occurred^34^. A similar nonmelanised appressorium-like swelling with successful leaf penetration has been reported in melanin-deficient mutants (Δ*pks1*) of the poplar pathogen *Colletotrichum gloeosporioides* (*Cglo*) CFCC80308^35^. These atypical intermediate forms are presumed to be HTE derivatives owing to their morphological immaturity, characterised by outline extension from the germ tube, indistinct septation, lack of melanisation, and greater invasiveness than MAE^34,35^. Collectively, these reports reinforce that unicellular appressorial maturation and melanisation are tightly linked in conventional appressorium-based plant infections by *Colletotrichum* and *Pyricularia* fungi.

Interestingly, melanin-deficient mutants of *Colletotrichum graminicola* (*Cgra*) CgM2 (Δ*pks1*) and *Cglo* EX2016-02 (Δ*scd1*) have been reported to exhibit normal turgor generation in morphologically mature, nonmelanised appressoria; however, these structures were more susceptible to external CWDEs than wild-type melanised appressoria^20,21^. These reports partially challenged the notion that appressorial melanisation is essential for appressorial functions, particularly in turgor generation. The identification of the semipermeability control by DHHA polymers resolved the contradiction between melanisation and turgor generation; the melanin layer provides cell wall rigidity to withstand high turgor pressure^15^. Notably, Δ*pks1* mutants of *Cgra* CgM2 failed to invade host plants^20^. Similarly, Δ*scd1* mutants of *Cglo* EX2016-02 exhibited minimal hydrogen peroxide accumulation on the host surface, indicating penetration defects^21^. These observations indicate that the theoretical prevention of MAE-based plant infections by *Colletotrichum* using MBIs remains unchallenged. Therefore, the roles of melanisation in appressorium-based plant invasion by *Colletotrichum* and *Pyricularia* remain unclear.

In the present study, we identified and characterised a group of *Colletotrichum* fungi that can invade plants via nonmelanised appressorium-mediated entry (NMAE) and rediscovered HTE-type strains. Our findings challenge the prevailing notion that appressorial formation and melanisation are universally essential for plant infections by *Colletotrichum*.

## Results

### MBI-insensitive *Colletotrichum* fungus

**Fig. 1:**
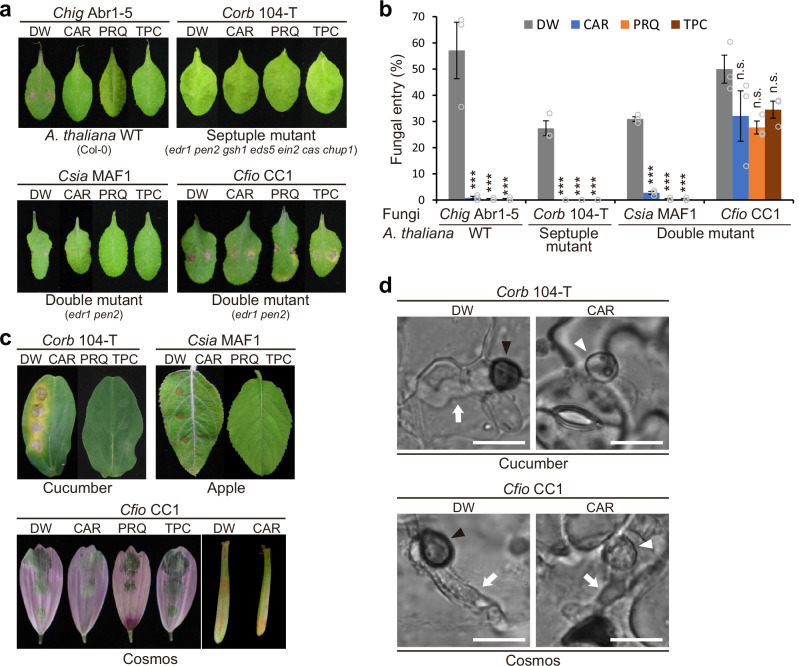
Plant invasion and lesion formation of cosmos anthracnose fungus *Colletotrichum fioriniae* CC1 that is insensitive to agrochemical melanin biosynthesis inhibitors (MBIs). **a** Efficacy of MBIs against *Colletotrichum* fungi-induced lesion formation in *Arabidopsis* mutants. A conidial suspension of adapted *Chig* Abr1-5 and nonadapted *Corb* 104-T, *Csia* MAF1, and *Cfio* CC1 was inoculated onto the leaves of wild-type *Arabidopsis* (Col-0), double (*edr1 pen2*), and septuple (*edr1 pen2 gsh1 eds5 ein2 cas chup1*) immunocompromised mutants in the presence or absence of MBIs, carpropamid (CAR), pyroquilon (PRQ), and tolprocarb (TPC) and incubated for 7 d (except for the inoculum of *Corb* 104-T, which was incubated for 20 d). **b** Fungal invasion of *Arabidopsis* epidermis. Each conidial suspension was inoculated onto the cotyledons of the indicated *Arabidopsis* mutants, with or without MBIs. The entry ratio was quantified at 4 d post-inoculation (dpi). Only the inoculum of *Chig* Abr1-5 was analysed at 3 dpi. At least 100 appressoria were investigated. The mean and SE were calculated from three independent plants. Asterisks indicate significant differences from the control (DW): ***P < 0.001, one-way analysis of variance with Dunnett’s test. n.s.: not significant. **c** Efficacy of MBIs on the pathogenicity of *Colletotrichum* fungi in host plants. A conidial suspension of *Corb* 104-T, *Csia* MAF1, and *Cfio* CC1 was inoculated onto cucumber cotyledons, apple leaves, and cosmos petals and cotyledons, with or without MBIs, and incubated for 7, 15, 2, and 12 d. **d** Fungal invasion of host epidermis at 4 dpi. Each fungus was inoculated onto the cotyledons of host plants with or without CAR. Similar results were observed in three independent experiments and the representative images were shown. Arrowheads indicate melanised (black) and nonmelanised (white) appressoria. Arrows indicate invasive hyphae. Scale bar = 10 µm.

*Arabidopsis thaliana* with mutations in multiple immune components allows the MAE of nonadapted *Colletotrichum*, suggesting that immunocompromised *A. thaliana* could be used as a tool to screen for fungal MAE ability^36^. To confirm the sensitivity of four *Colletotrichum* strains^36^ to different types of MBIs—MBI-D carpropamid, MBI-R pyroquilon, and MBI-P tolprocarb (Supplementary Fig. 1)—we inoculated wild-type (Col-0), double (*edr1 pen2*), or septuple (*edr1 pen2 gsh1 eds5 ein2 cas chup1*) immunocompromised mutants of *A. thaliana* with adapted *Colletotrichum higginsianum* (*Chig*) Abr1-5 and nonadapted *Colletotrichum orbiculare* (*Corb*) 104-T, *Colletotrichum siamense* (*Csia*) MAF1, and *Colletotrichum fioriniae* (*Cfio*) CC1, respectively, in the presence or absence of MBIs (Fig. 1a). As expected, lesion formation by *Chig* Abr1-5, *Corb* 104-T, and *Csia* MAF1 was strongly prevented in the presence of MBIs, although *Corb* 104-T formed small lesions even on septuple mutants. Notably, *Cfio* CC1 exhibited strong insensitiveness to all MBIs and produced prominent lesions on *A. thaliana* mutants (Fig. 1a). Consistent with the degree of lesion formation, *Cfio* CC1 invaded the *A. thaliana* epidermis through appressoria, even in the presence of MBIs (Fig. 1b and Supplementary Fig. 3). The appressoria of *Cfio* CC1 were morphologically mature and exhibited sufficient sensitivity to MBIs in terms of melanisation. However, the nonmelanised appressoria formed invasive hyphae within the plant epidermis (Supplementary Fig. 3).

We subsequently examined the effects of MBIs on compatible interactions between *Colletotrichum* fungi and host plants (Fig. 1c). As in the case of the *A. thaliana*–*Chig* Abr1-5 interaction (Fig. 1a), we confirmed the preventive effects of MBIs on lesion formation by *Corb* 104-T and *Csia* MAF1 in their host plants, cucumber and apple, respectively (Fig. 1c). In contrast, *Cfio* CC1 demonstrated clear insensitiveness to MBIs and developed lesions on the petals and cotyledons of its host cosmos (Fig. 1c). In the presence of MBIs, only *Cfio* CC1 formed invasive hyphae in the host epidermis via nonmelanised appressoria, similar to the immunocompromised *A. thaliana* mutants (Fig. 1d and Supplementary Fig. 3). These results indicate a potential dogma-breaking *Colletotrichum* fungus with NMAE capability.

### Unprecedented NMAE-type *Colletotrichum* fungus

**Fig. 2:**
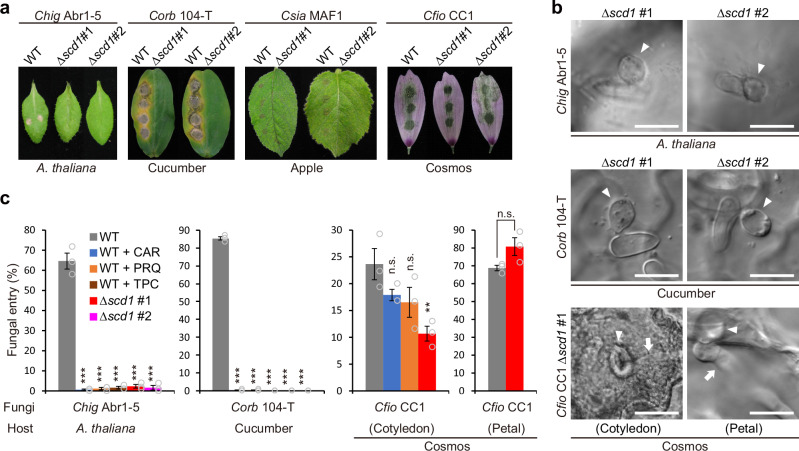
*Colletotrichum fioriniae* CC1 is an unprecedented nonmelanised appressorium-mediated entry (NMAE)-type pathogen. **a** Pathogenicity of *Colletotrichum* fungi in host plants. A conidial suspension of wild-type or melanin-deficient (Δ*scd1*) mutants of *Chig* Abr1-5, *Corb* 104-T, *Csia* MAF1, and *Cfio* CC1 was inoculated onto *Arabidopsis thaliana* leaves, cucumber cotyledons, apple leaves, and cosmos petals and incubated for 7, 7, 12, and 4 d. **b** Fungal invasion of the host epidermis. A conidial suspension of Δ*scd1* mutants of *Chig* Abr1-5, *Corb* 104-T, and *Cfio* CC1 was inoculated onto each host plant and observed at 3, 4, and 4 d post-inoculation (dpi). White arrowheads and arrows indicate nonmelanised appressoria and invasive hyphae, respectively. Scale bar = 10 µm. **c** Quantification of fungal invasion of host plants. A conidial suspension of the wild-type or Δ*scd1* mutants of each fungus was inoculated onto host plants. When necessary, melanin biosynthesis inhibitors, carpropamid (CAR), pyroquilon (PRQ), or tolprocarb (TPC) were added. The entry ratio was quantified at 4 dpi. Only the inoculum of *Chig* Abr1-5 was analysed at 3 dpi. At least 100 appressoria were investigated. The mean and SE were calculated from three independent samples. Asterisks indicate significant differences from the control: ***P < 0.001, **P < 0.01, one-way analyses of variance with Dunnett’s test or unpaired two-tailed *t*-test. n.s.: not significant.

To further verify the NMAE ability of *Cfio* CC1, we generated melanin-deficient (Δ*scd1*) mutants of the four *Colletotrichum* strains, all of which formed unicellular, morphologically mature, nonmelanised appressoria (Supplementary Fig. 4). Consistent with the MBI treatment assays, lesion formation by *Cfio* CC1 Δ*scd1* mutants was comparable to that of the wild-type, whereas the Δ*scd1* mutants of other strains were nonpathogenic to their host plants (Fig. 2a). Microscopic observations revealed that *Cfio* CC1 Δ*scd1* exhibited NMAE-based invasive hyphal development *in planta* (Fig. 2b), and the entry rates revealed a clear distinction in melanisation dependency between *Cfio* CC1 and other strains (Fig. 2c). Except for *Cfio* CC1, these typical phenotypes of MBI-treated wild-type (Fig. 1) or melanin-deficient mutants (Fig. 2 and Supplementary Fig. 4) of *Colletotrichum* strains were consistent with the long-standing dogma that melanisation was universally essential for plant invasion^5,6^. Therefore, we identified *Cfio* CC1 as an unprecedented MBI-insensitive *Colletotrichum* strain that causes anthracnose symptoms in plants via NMAE. Meanwhile, *Cfio* CC1 exhibited a slightly lower entry rate via NMAE than via MAE in cosmos cotyledons, suggesting that even in *Cfio* CC1, appressorial melanisation partly contributed to plant invasion under some conditions (Fig. 2c); this notion is supported by the plant immune responses. In *Arabidopsis* Col-0, the epidermal chloroplast response (ECR) was triggered by an entry attempt of *Cfio* CC1 as one of the lower-layer preinvasive defences^36,37^. However, ECR was rarely induced by *Cfio* CC1 Δ*scd1* melanin-deficient mutant, whereas it induced sufficient ECR in *pen2-1* plants, which were partly defective in the higher-layer preinvasive defences^36,37^ (Supplementary Fig. 5). This observation likely reflects a slight decrease in the penetration capability of *Cfio* CC1 Δ*scd1* compared with that of the wild-type.

### Appressorial functionality and melanisation

**Fig. 3:**
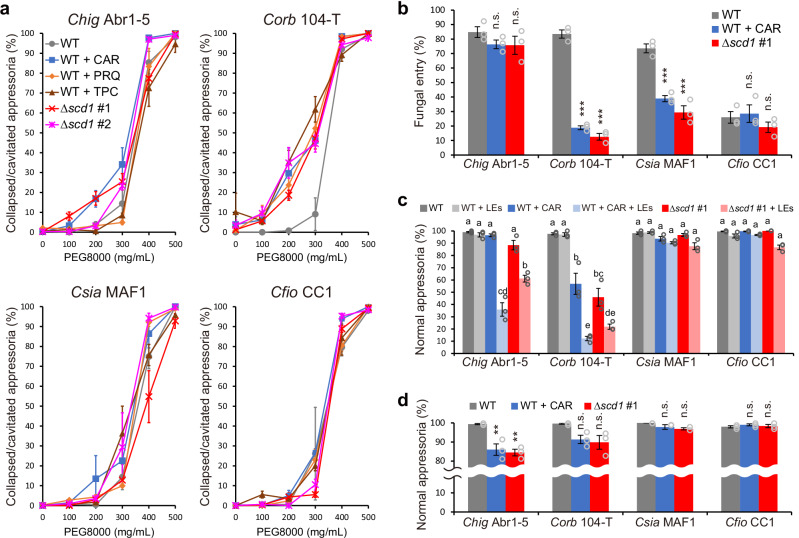
Melanisation dependency of appressorial functions varies among *Colletotrichum* fungi. **a** Appressorial turgor generation. Turgor was evaluated to assess the frequency of appressorial collapse or cavitation. The appressoria of wild-type or Δ*scd1* mutants of *Chig* Abr1-5, *Corb* 104-T, *Csia* MAF1, and *Cfio* CC1 that formed on glass surfaces were exposed to a range of PEG8000 concentrations. When necessary, melanin biosynthesis inhibitors, carpropamid (CAR), pyroquilon (PRQ), or tolprocarb (TPC) were added. At least 100 appressoria were investigated. The mean and SE were calculated from three independent samples. **b** Quantification of fungal entry into artificial substratum cellulose membranes. A conidial suspension of each fungus was incubated on cellophane for 24 (*Chig* Abr1-5 and *Corb* 104-T) or 48 h (*Csia* MAF1 and *Cfio* CC1). The entry ratio was quantified for at least 100 appressoria. The mean and SE were calculated from three independent samples. Asterisks indicate significant differences from the control (WT): ***P < 0.001, one-way analysis of variance with Dunnett’s test. n.s.: not significant. **c** Integrity of the appressorial cell wall against cell wall-degrading enzymes. Morphologically mature appressoria of each fungus were incubated with lysing enzymes (LEs) from *Trichoderma harzianum*. At least 90 appressoria were investigated in each sample. The mean and SE were calculated from three independent samples. Means with different letters significantly differ (*P* < 0.05, two-way analysis of variance with Tukey’s HSD). **d** Durability of appressoria on plants. A conidial suspension of each fungus was inoculated onto *A. thaliana* Col-0. The proportion of normally shaped appressoria was quantified at 24 h post-inoculation. At least 100 appressoria on each plant were investigated. The mean and SE were calculated from three independent plants. Asterisks indicate significant differences from the control (WT): **P < 0.01, one-way analysis of variance with Dunnett’s test. n.s.: not significant.

Based on the NMAE ability of *Cfio* CC1, we hypothesised that this strain accomplishes the appressorial functions required for plant invasion without relying on melanisation. Exposure of the appressorium to external high osmotic stress causes collapse through incipient cytorrhysis or cavitation through cytoplasmic water loss^38^ (Supplementary Fig. 6a) and this assay is valuable for estimating appressorial turgor pressure^12,13,15,20,21,27,35,39–51^. Consistent with previous reports^13,52^, the nonmelanised appressoria of *Corb* 104-T exhibited reduced resistance to external high osmotic stress, suggesting melanisation-dependent turgor generation and/or cell wall rigidity in the appressoria^15^ (Fig. 3a). Notably, similar to *Cgra* CgM2^20^ and *Cglo* EX2016-02^21^, the appressoria of *Chig* Abr1-5, *Csia* MAF1, and *Cfio* CC1 generated sufficient turgor pressure, regardless of melanisation (Fig. 3a). The results of these five *Colletotrichum* species were consistent with the recent finding that DHHA polymers, but not melanin, directly contributed to the pore size reduction of appressorial cell wall and consequent turgor generation^15^. We also confirmed the melanisation dependency of *P. oryzae* in the same appressorial turgor evaluation assay (Supplementary Fig. 7a). Collectively, only the data of the well-studied *Corb* 104-T and *P. oryzae* aligned with the long-accepted theory on melanisation-dependent turgor and/or cell wall rigidity in appressoria (Fig. 3a and Supplementary Fig. 7a). To further strengthen the notion that the appressorial turgor and/or cell wall rigidity in most *Colletotrichum* fungal strains is essentially melanisation-independent, we evaluated the turgor of another two *Colletotrichum* strains belonging to different species (Supplementary Fig. 7b). As expected, the appressoria of *Colletotrichum nymphaeae* (*Cnym*) GCP26 and *Colletotrichum godetiae* (*Cgod*) MC1 exhibited melanisation-independent turgor generation (Supplementary Fig. 7b).

We tested appressorial penetration ability using artificial cellulose membranes (Fig. 3b). Consistent with previous reports^15,26,53^, the nonmelanised appressoria of *Corb* 104-T frequently formed lateral germ tube and secondary appressoria; however, even normally shaped appressoria drastically lost their cellulose membrane-penetrating ability, suggesting melanisation-dependent physical force generation for artificial substratum penetration (Fig. 3b and Supplementary Fig. 6b). Upon melanisation inhibition, *Csia* MAF1 occasionally formed aberrantly shaped appressoria on the cellulose membranes, with penetration efficiency almost halved, whereas appressorial formation and penetration in *Chig* Abr1-5 and *Cfio* CC1 in vitro remained unaffected (Fig. 3b and Supplementary Fig. 6b).

Melanisation-dependent resistance of appressoria to external CWDEs has been reported in some *Colletotrichum* fungi^20,21^. Therefore, we investigated the effects of lysing enzymes (LEs) on the appressorial cell wall integrity of the four strains. We found that CWDE resistance of appressoria in *Chig* Abr1-5 depended on melanisation, as we observed extrusion of cytoplasmic contents in ruptured nonmelanised appressoria (Fig. 3c and Supplementary Fig. 6c). Notably, the nonmelanised appressoria of *Corb* 104-T moderately collapsed even in the absence of LEs and further collapsed by subsequent LE application, suggesting further decreased cell wall rigidity compared to other strains (Fig. 3c and Supplementary Fig. 6c). We did not detect any difference in CWDE resistance between melanised and nonmelanised appressoria in *Csia* MAF1 and *Cfio* CC1 (Fig. 3c and Supplementary Fig. 6c).

To evaluate the durability of the nonmelanised appressoria developed on the plant surface, we compared the melanised and nonmelanised appressoria of each *Colletotrichum* strain on *Arabidopsis* cotyledons. Only *Chig* Abr1-5 demonstrated a statistically significant decrease in the proportion of normally shaped appressoria under melanisation-inhibiting conditions (Fig. 3d and Supplementary Fig. 6d). This observation is similar to that described in a previous report, in which the appressoria of *Cgra* CgM2 melanin-deficient mutants on host leaves ruptured more frequently than those of the wild-type^20^. In contrast, the nonmelanised appressoria of *Corb* 104-T, *Csia* MAF1, and *Cfio* CC1 remained structurally intact, although *Corb* 104-T exhibited a decreasing trend in the proportion of normally shaped appressoria under melanisation-inhibiting conditions (Fig. 3d and Supplementary Fig. 6d).

Hence, in addition to the data on plant invasion and lesion formation via appressorium-mediated entry (Figs. 1 and 2, and Supplementary Fig. 3), these results demonstrate that the roles of appressorial melanisation during plant invasion vary among *Colletotrichum* fungi (Supplementary Fig. 8). Of these, *Cfio* CC1 maintained almost all plant invasion functions in nonmelanised appressoria, thus being capable of exhibiting NMAE.

### A group of NMAE-type *Colletotrichum* fungi

**Fig. 4:**
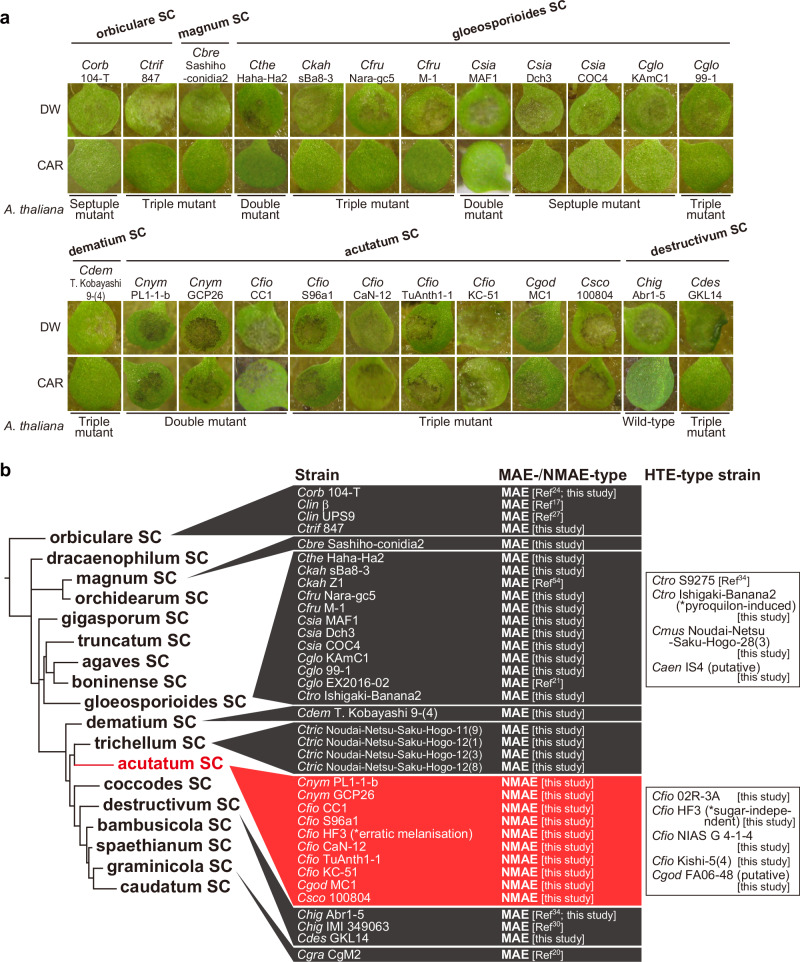
Emergence of a nonmelanised appressorium-mediated entry (NMAE)-type *Colletotrichum* fungal group. **a** Candidate screening for NMAE-type *Colletotrichum* fungi capable of inducing lesions in plants in the presence of melanin biosynthesis inhibitors. An adapted *C. higginsianum* (*Chig*) Abr1-5 and various nonadapted *Colletotrichum* strains belonging to various species complexes (SCs), *C. orbiculare* (*Corb*) 104-T, *C. trifolii* (*Ctrif*) 847, *C. brevisporum* (*Cbre*) Sashiho-conidia2, *C. theobromicola* (*Cthe*) Haha-Ha2, *C. kahawae* (*Ckah*) sBa8-3, *C. fructicola* (*Cfru*) Nara-gc5, *Cfru* M-1, *C. siamense* (*Csia*) MAF1, *Csia* Dch3, *Csia* COC4, *C. gloeosporioides* (*Cglo*) KAmC1, *Cglo* 99-1, *C. dematium* (*Cdem*) T. Kobayashi 9-(4), *C. nymphaeae* (*Cnym*) PL1-1-b, *Cnym* GCP26, *C. fioriniae* (*Cfio*) CC1, *Cfio* S96a1, *Cfio* CaN-12, *Cfio* TuAnth1-1, *Cfio* KC-51,* C. godetiae* (*Cgod*) MC1, *C. scovillei* (*Csco*) 100804, and *C. destructivum* (*Cdes*) GKL14, were inoculated onto cotyledons of *Arabidopsis thaliana* double (*edr1 pen2*), triple (*edr1 pen2 gsh1*), or septuple (*edr1 pen2 gsh1 eds5 ein2 cas chup1*) immunocompromised mutants with or without carpropamid (CAR) and incubated for 5 d. Only inocula of *Ctrif* 847 and *Cdem* T. Kobayashi 9-(4) were incubated for 7 d. **b** Phylogenetic relationships between MAE-/NMAE-type strains and *Colletotrichum* SC. A simplified illustration of the phylogenetic tree was generated based on the phylogeny of *Colletotrichum* species^10^. NMAE-type strains are highlighted in red. The classification of hyphal tip-based entry (HTE)-type strains is presented on the right side of the information on MAE- and NMAE-type strains.

To explore the relationship between NMAE capability and the evolution and phylogeny of *Colletotrichum* fungi, we comprehensively screened NMAE-type strains based on lesion formation in immunocompromised *A. thaliana* mutants in the presence of MBI (Fig. 4a and Supplementary Fig. 9a). Of the 24 *Colletotrichum* strains capable of MAE-based invasion and inducing lesions on either *Arabidopsis* mutant in the absence of MBI, we further identified 8 MBI-insensitive strains: *Cnym* PL1-1-b, *Cnym* GCP26, *Cfio* S96a1, *Cfio* CaN-12, *Cfio* TuAnth1-1, *Cfio* KC-51, *Cgod* MC1, and *Colletotrichum scovillei* (*Csco*) 100804, in addition to *Cfio* CC1 (Fig. 4a and Supplementary Fig. 10). These MBI-insensitive strains, similar to *Cfio* CC1, successfully invaded the plant epidermis and developed invasive hyphae via definitive NMAE (Supplementary Fig. 10). Notably, as indicated in the phylogenetic tree of *Colletotrichum* species complexes (SCs)^10^, these unprecedented strains with NMAE traits clustered into a single monophyletic group, the acutatum SC (Fig. 4b). As for the trichellum SC, which is closely related to acutatum SC^10^ (Fig. 4b), we did not confirm lesion formation attributed to the four *Colletotrichum trichellum* (*Ctric*) strains, even on *A. thaliana* septuple mutants, similar to other strains belonging to various SCs (Supplementary Fig. 9a). These four *Ctric* strains developed morphologically mature and melanised appressoria with sufficient sensitivity to MBI in terms of melanisation on *A. thaliana* (Supplementary Fig. 9b). Therefore, we examined the effects of MBI on their invasion and pathogenicity in the host Japanese ivy and determined their MBI sensitivity (Supplementary Fig. 9c, d), leading to the characterisation of *Ctric* strains as conventional MAE-type (Fig. 4b). On the surface of *A. thaliana*, all MBI-sensitive *Colletotrichum* strains formed nonmelanised appressoria but were arrested at the prepenetration stage in the presence of MBI; therefore, they were classified as conventional MAE-type strains (Supplementary Fig. 10). In addition to previously characterised strains^17,20,21,24,27,30,34,54^, conventional MAE-type strains were highly dispersed across the phylogenetic tree, except for the acutatum SC; they belonged to orbiculare, magnum, gloeosporioides, dematium, trichellum, destructivum, or graminicola SCs^10^ (Fig. 4b). These findings suggest that a common ancestral line to the acutatum SC acquired a NMAE capability during the evolution, resulting in the emergence of NMAE-type *Colletotrichum* fungi.

### Spontaneous NMAE-based plant infection

**Fig. 5:**
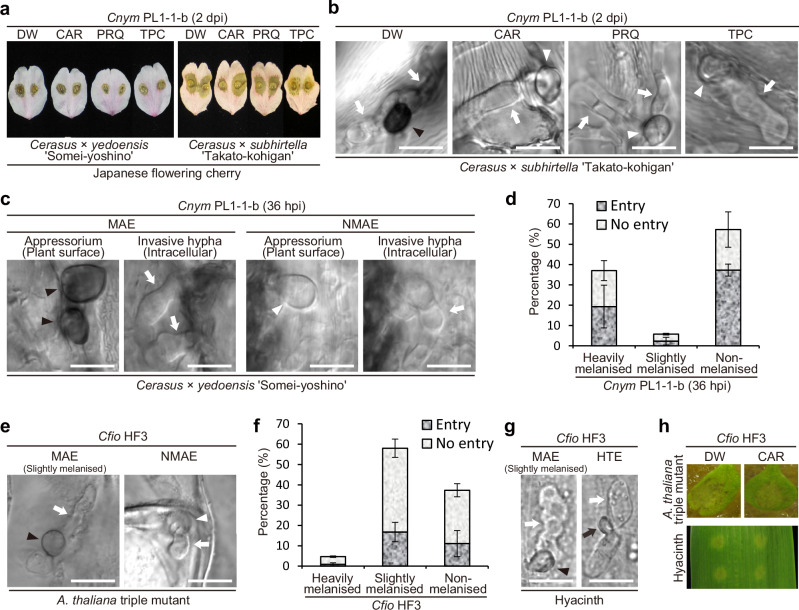
Some *Colletotrichum* fungi infect plants via spontaneous nonmelanised appressorium-mediated entry (NMAE). **a** Pathogenicity of *Cnym* PL1-1-b on host plants. A conidial suspension was inoculated onto the petals of Japanese flowering cherry cvs. ‘Somei-yoshino’ and ‘Takato-kohigan’ and incubated for 2 d. When necessary, melanin biosynthesis inhibitors, carpropamid (CAR), pyroquilon (PRQ), or tolprocarb (TPC) were added. **b** Fungal invasion of *Cnym* PL1-1-b into the host epidermis at 2 d post-inoculation (dpi). Similar results were observed in three independent experiments and the representative images were shown. Black and white arrowheads indicate melanised and nonmelanised appressoria, respectively. Arrows indicate invasive hyphae. Scale bar = 10 µm. **c**, **d** Early invasion of *Cnym* PL1-1-b into the host epidermis via NMAE at 36 h post-inoculation (hpi). Black and white arrowheads indicate melanised and nonmelanised appressoria, respectively. Arrows indicate invasive hyphae. Scale bar = 10 µm. The entry ratio was then quantified. At least 70 appressoria on each petal were investigated. The mean and SE were calculated for five independent petals. **e**, **f** Fungal invasion of *Cfio* HF3 into *A. thaliana* triple mutant (*edr1 pen2 gsh1*). A conidial suspension was inoculated onto the cotyledons and incubated for 4 d. Black and white arrowheads indicate slightly melanised and nonmelanised appressoria, respectively. Arrows indicate invasive hyphae. Scale bar = 10 µm. The entry ratio was then quantified. One hundred appressoria were investigated. The mean and SE were calculated from nine independent plants. **g** Fungal invasion of *Cfio* HF3 into host hyacinth via melanised appressorium-mediated entry (MAE) or hyphal tip-based entry (HTE). Similar results were observed in three independent experiments and the representative images were shown. Arrowhead indicates a slightly melanised appressorium. Black and white arrows indicate the elongated germ tube-like and invasive hyphae, respectively. Scale bar = 10 µm. A conidial suspension was inoculated onto the cotyledons and incubated for 3 d. **h** Inhibitory effects of CAR on *Cfio* HF3-induced lesion formation in *A. thaliana* triple mutants and hyacinth at 5 dpi. When necessary, CAR was added.

Although we identified NMAE-type *Colletotrichum* fungi under MBI-treated conditions where appressorial melanisation was artificially inhibited (Figs. 1–4 and Supplementary Figs. 3–10), it remained unclear whether NMAE-based plant invasion and infection by *Colletotrichum* fungi occur under MBI-free conditions. *Cnym* PL1-1-b, a causal agent of anthracnose disease in Japanese flowering cherry, induced necrotic lesions on petals of flowering cherry cultivars ‘Somei-yoshino’ and ‘Takato-kohigan’, regardless of the presence or absence of MBIs (Fig. 5a). This observation was attributed to NMAE-based invasion into host epidermis (Fig. 5b). Similar results were observed in the inoculation assay of immunocompromised mutants of the nonhost *A. thaliana* (Supplementary Fig. 11). Notably, in host plant tissue, *Cnym* PL1-1-b displayed sufficient invasive hyphal development and lesion formation at 2 d post-inoculation (dpi). Given that *Colletotrichum* fungi usually require at least a few dpi for conventional MAE-based plant invasion and that subsequent lesion formation on plants lags behind fungal entry owing to their hemibiotrophic characteristics, we speculated that *Cnym* PL1-1-b might penetrate the host epidermis via appressoria before melanisation. As expected, at an early time point [36 h post-inoculation (hpi)], we observed melanised and nonmelanised appressoria simultaneously, both of which had well-developed invasive hyphae in the host tissue without hindrance (Fig. 5c, d). These results indicate that *Cnym* PL1-1-b retains almost full appressorial functionality for penetration in the premelanisation stage and subsequently infects the host plants via spontaneous NMAE.

Notably, we further identified an impressive strain, *Cfio* HF3, that vigorously formed morphologically mature appressoria with poor melanisation on plant surfaces, even in the absence of MBIs (Fig. 5e–g). Consistent with the acutatum SC, *Cfio* HF3 exhibited NMAE and induced lesions on immunocompromised *A. thaliana* mutants regardless of the presence or absence of MBIs (Figs. 4b and 5e, f, h upper panel). Additionally, *Cfio* HF3 exhibited MBI-insensitive infection of its host hyacinth, and in the absence of MBIs, this pathogen invaded the host epidermis via slightly melanised appressoria, while sugar-independent HTE was also confirmed (Fig. 5g, h lower panel). Collectively, these results indicate that *Cfio* HF3 naturally follows melanisation-independent strategies to infect plants.

### Early plant invasion via NMAE and HTE

**Fig. 6:**
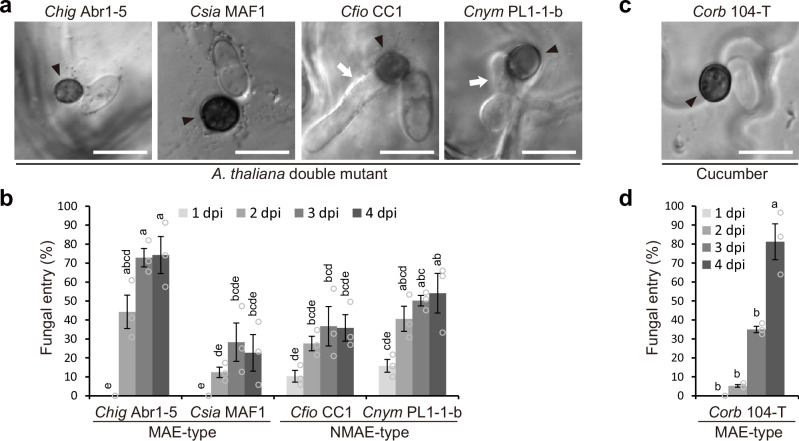
Nonmelanised appressorium-mediated entry (NMAE)-type pathogens invade plants earlier than melanised appressorium-mediated entry (MAE)-type pathogens. **a** Fungal invasion of MAE-type *Chig* Abr1-5 and *Csia* MAF1 and NMAE-type *Cfio* CC1 and *Cnym* PL1-1-b into *Arabidopsis thaliana* double mutants (*edr1 pen2*) at 1 d post-inoculation (dpi). A conidial suspension was inoculated onto the cotyledons and incubated. Black arrowheads and white arrows indicate melanised appressoria and invasive hyphae, respectively. Scale bar = 10 µm. **b** Quantification of fungal invasion into the cotyledons of *A. thaliana* double mutants. The entry ratio was quantified every 24 h. At least 100 appressoria on each plant were investigated. The mean and SE were calculated from three independent plants. Means with different letters significantly differ (*P* < 0.05, two-way analysis of variance with Tukey’s HSD). **c** Noninvasion of MAE-type *Corb* 104-T into host cucumbers at 1 dpi. A conidial suspension was inoculated onto cotyledons and incubated. The black arrowhead indicates the melanised appressorium. Scale bar = 10 µm. **d** Quantification of fungal invasion in cucumber cotyledons. The entry ratio was quantified every 24 h. At least 100 appressoria were investigated on each plant. The mean and SE were calculated from three independent plants. Means with different letters significantly differ (*P* < 0.05, one-way analysis of variance with Tukey’s HSD).

We hypothesised that early host invasion by *Cnym* PL1-1-b implies that NMAE-type *Colletotrichum* fungi might be inherently capable of penetrating the plant epidermis earlier than conventional MAE-type fungi owing to their melanisation-independent appressorial functionality. Therefore, we evaluated the fungal entry of MAE-type *Chig* Abr1-5 and *Csia* MAF1 and NMAE-type *Cfio* CC1 and *Cnym* PL1-1-b into immunocompromised *A. thaliana* double mutants every 24 h until 4 dpi. *Cfio* CC1 and *Cnym* PL1-1-b successfully penetrated the plant epidermis via appressoria and developed invasive hyphae by 1 dpi (Fig. 6a, b). In contrast, *Chig* Abr1-5 and *Csia* MAF1 formed melanised appressoria; however, they never developed invasive hyphae in the plant epidermis at 1 dpi, even though *Chig* Abr1-5 adapted to the host *A. thaliana* (Fig. 6a, b). MAE-type *Corb* 104-T did not exhibit MAE in the epidermal cells of its host cucumber at 1 dpi, corroborating the differences in penetration timing between NMAE- and MAE-type pathogens (Fig. 6c, d). These data indicate that melanisation-independent appressorial functionality in NMAE-type *Colletotrichum* strains accelerates penetration timing compared with that in conventional MAE-type strains.

**Fig. 7:**
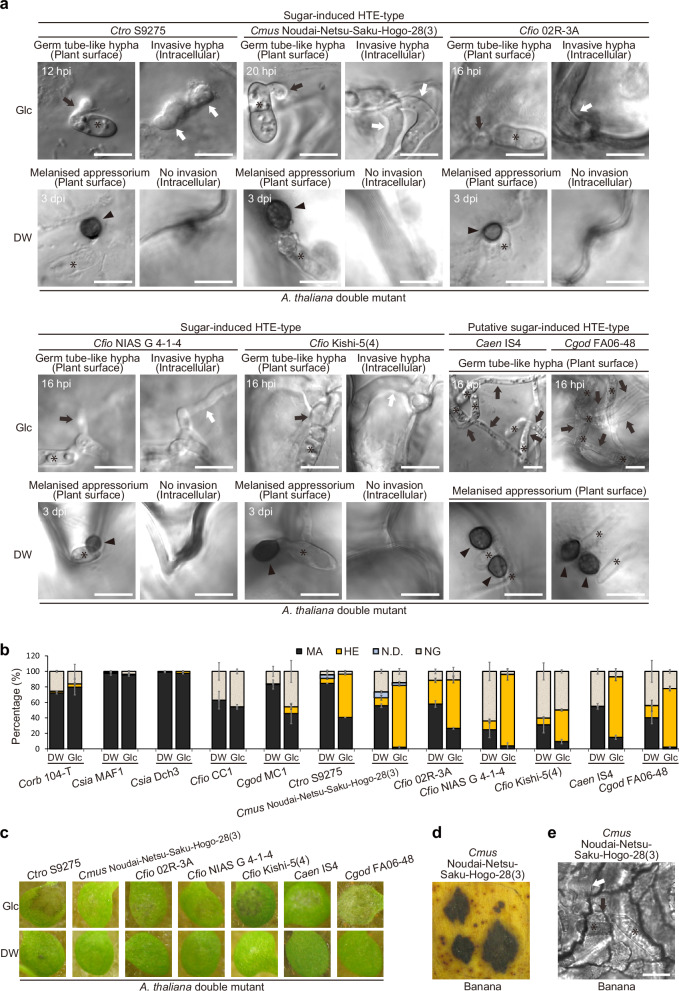
Several sugar-induced hyphal tip-based entry (HTE)-type *Colletotrichum* fungi were identified within the gloeosporioides and acutatum species complexes (SCs). **a** Identification of sugar-induced and putatively sugar-induced HTE-type *Colletotrichum* fungi. A conidial suspension of nonadapted *Colletotrichum* strains, *C. tropicale* (*Ctro*) S9275, *C. musae* (*Cmus*) Noudai-Netsu-Saku-Hogo-28(3), and *C. aenigma* (*Caen*) IS4 belonging to the gloeosporioides SC, and *C. fioriniae* (*Cfio*) 02R-3A, *Cfio* NIAS G 4-1-4, *Cfio* Kishi-5(4), and *C. godetiae* (*Cgod*) FA06-48 belonging to the acutatum SC, were inoculated onto the cotyledons of *Arabidopsis thaliana* double (*edr1 pen2*) immunocompromised mutants with or without glucose (Glc), and incubated for 12–20 h. Black arrowheads indicate melanised appressoria. Black and white arrows indicate the elongated germ tube-like and invasive hyphae, respectively. Asterisks indicate conidia. Scale bar = 10 µm. **b** Quantification of MAE- and HTE-related morphogenesis of *Colletotrichum* fungi in the presence of Glc. A conidial suspension was inoculated onto the cotyledons of *A. thaliana* Col-0, with or without Glc, and incubated for 16 h. The proportion of the conidia exhibiting melanised appressorium formation (MA), hyphal extension (HE), or no germination (NG) was then quantified. N.D. indicates not determined because of the formation of a short germ tube whose subsequent morphogenesis is unpredictable. At least 100 conidia on each plant were investigated. The mean and SE were calculated from three independent plants. **c** Increased lesion formation of sugar-induced HTE-type pathogens in the presence of sugar. A conidial suspension of each *Colletotrichum* strain was inoculated onto the cotyledons of *A. thaliana* double (*edr1 pen2*) mutants with or without Glc and incubated for 3 d. **d** Pathogenicity of *Cmus* Noudai-Netsu-Saku-Hogo-28(3) on host plants. A conidial suspension was inoculated onto the ripe fruits of the Banana cv. ‘Cardaba’ and incubated for 5 d. **e** Microscopic observations of banana epidermis inoculated with *Cmus* Noudai-Netsu-Saku-Hogo-28(3) at 5 dpi. Similar HTE-type morphogenesis was observed in three independent experiments and the representative images were shown. Black and white arrows indicate the elongated germ tube-like and invasive hyphae, respectively. Asterisks indicate conidia. Scale bar = 10 µm.

Similar early invasion of *A. thaliana pen2-1* mutants by appressorium-independent HTE of *Ctro* S9275 has also been reported; invasive hyphae frequently develop from the tip of the elongated germ tube-like hyphae by 14 hpi^34^. Under HTE-inducing conditions, appressorial formation and pigmentation were suppressed, but *Ctro* S9275 occasionally formed an immature appressorium-like swelling at the hyphal tip, as presumed for HTE derivatives with invasive hyphae^34^. However, these unconventional entry modes of *Ctro* S9275 at ~14 hpi were induced by sugar and were more invasive and occurred earlier than the NMAE of *Cfio* CC1 and *Cnym* PL1-1-b at 24 hpi against *Arabidopsis edr1 pen2* mutants, suggesting that HTE is distinct from NMAE^55^ (Fig. 6a, b). More importantly, in the presence of sugar, *Ctro* S9275 occasionally developed morphologically mature, fully melanised conventional appressoria but scarcely exhibited MAE in *pen2-1* mutants of nonhost *Arabidopsis*^34^, whereas *Cnym* PL1-1-b simultaneously demonstrated MAE and NMAE (Fig. 5c, d). These data emphasise the discrepancy between appressorium-mediated entry (MAE and NMAE) and HTE. To assess the differences among MAE, NMAE, and HTE from the viewpoint of phylogeny, we searched for HTE-type *Colletotrichum* strains other than *Ctro* S9275^34^ and *Cfio* HF3 (Fig. 5g). Based on microscopic observations, we identified *Colletotrichum musae* (*Cmus*) Noudai-Netsu-Saku-Hogo-28(3), *Cfio* 02R-3A, *Cfio* NIAS G 4-1-4, and *Cfio* Kishi-5(4) exhibiting sugar-induced suppression of appressorial development and HTE in immunocompromised *A. thaliana* mutants at early time points, similar to *Ctro* S9275^34^ (Fig. 7a, b). Notably, a previous study has characterised the infection process of HTE as a shared entry with MAE through penetration peg-like structures, excluding appressorium formation, based on staining of papillary callose deposition, a defence response marker beneath the fungal penetration pegs, and microscopic observation^34^. Consistently, we confirmed pathogen-induced papilla beneath successful fungal entry sites, where callose deposition was excluded from the centre of papillae, due to fungal penetration (Supplementary Fig. 12). These sugar-induced HTE-type strains developed morphologically mature, fully melanised appressoria in the absence of sugar but scarcely exhibited MAE (Fig. 7a, b), corroborating the more invasive penetration and increased lesion formation by HTE compared with MAE (Fig. 7a, c). We also identified *Colletotrichum aenigma* (*Caen*) IS4 and *Cgod* FA06-48, which displayed appressorial formation suppression, germ tube-like hyphal extension, and increased lesion formation in the presence of sugar, all of which are common characteristics of HTE^34^. These observations suggest that these strains are putative sugar-induced HTE-type, although their HTE sites could not be confirmed because the elongated hyphae grew too long and covered the surface of the plants at 16 hpi (Fig. 7). In contrast, some tested MAE- and NMAE-type strains stably formed melanised appressoria on the surface of *A. thaliana* mutants, even in the presence of sugar at 16 hpi (Supplementary Fig. 13). Notably, the pathogenicity of *Cmus* Noudai-Netsu-Saku-Hogo-28(3) on host banana was attributed to HTE-based invasion, as this pathogen scarcely formed appressoria on the host surface but exhibited HTE, which is induced by sugar and/or other ripe fruit-derived signal(s) (Fig. 7d, e). The HTE- and putative HTE-type strains were classified as gloeosporioides and acutatum SC members, differing from the phylogenetically broadly distributed conventional MAE-type and acutatum SC-specific NMAE-type strains; this observation further supports the differences between MAE, NMAE and HTE (Fig. 4b).

### Plant species-specific lesion formation by NMAE-type *Colletotrichum* fungi in melanisation inhibition conditions

Most *Colletotrichum* species in acutatum SC are polyphagous^56^. NMAE-type *Cfio* CC1, which was originally isolated from cosmos, could induce lesions on the leaves of several herbaceous dicot and monocot plants (Supplementary Fig. 14a-e). Therefore, *Cfio* CC1 is presumed to be polyphagous, although these visible lesions may reflect hypersensitive response-like cell death triggered by fungal invasions, attributed to plant immunity. As previously demonstrated, *Cfio* CC1 invaded cosmos cotyledons, partly through appressorial melanisation (Fig. 2c). Therefore, to gain further insight into the melanisation dependence of NMAE-type strains for plant invasion, we examined the ability of the *Cfio* CC1 Δ*scd1* melanin-deficient mutant to cause lesions in these plants (Supplementary Fig. 14a–e). Similar to cosmos and *A. thaliana* double mutants, NMAE-type *Cfio* CC1 formed lesions on spinach and rice regardless of appressorial melanisation (Supplementary Fig. 14a, e). We also confirmed that *Cfio* CC1 Δ*scd1* developed NMAE-based invasive hyphae in the rice epidermis to the same extent as MAE-based invasion by wild-type *Cfio* CC1 (Supplementary Fig. 14f). In contrast, *Cfio* CC1 exhibited a strong melanisation dependency in lesion formation in some plant species, such as cockscomb, maize, and sorghum (Supplementary Fig. 14b–d). The lesion-causing defects of *Cfio* CC1 Δ*scd1* were restored in heat shock-pretreated leaves, where plant immunity might be partly compromised (Supplementary Fig. 14b–d). To verify generalisability, we performed inoculation assays of NMAE-type strains on woody plants and obtained similar results. Specifically, even in the presence of MBI, *Cfio* CC1 produced lesions on sacred bamboo wild-type and cv. Otafukunanten, and *Cfio* S96a1 and *Cgod* MC1 formed lesions on Japanese summer orange, whereas lesion formation by *Cfio* CC1 on Japanese andromeda was prevented by MBI (Supplementary Fig. 15). These data indicate that the essentiality of appressorial melanisation for lesion formation depends on the interactions between NMAE-type *Colletotrichum* fungus and plant species; some plants might fulfil sufficient preinvasive defences against NMAE but not against MAE by the same pathogen.

### Pyroquilon-specific induction of *Colletotrichum* HTE and lesion formation

**Fig. 8:**
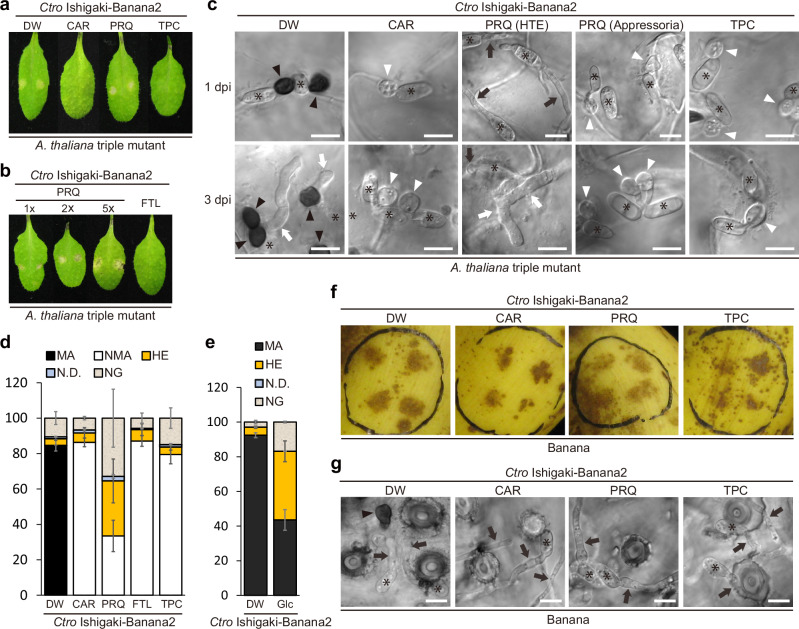
Pyroquilon-induced hyphal tip-based entry (HTE)-type *Colletotrichum* fungus is identified. **a** Efficacy of melanin biosynthesis inhibitors (MBIs), carpropamid (CAR), pyroquilon (PRQ), and tolprocarb (TPC), against nonadapted *C. tropicale* (*Ctro*) Ishigaki-Banana2-induced lesion formation in *Arabidopsis* triple mutants (*edr1 pen2 gsh1*). The photograph was taken at 7 d post-inoculation (dpi). **b** Efficacy of MBI-Rs, PRQ or fthalide (FTL), against *Ctro* Ishigaki-Banana2-induced lesion formation. The photograph was taken at 7 dpi. The concentration of PRQ was varied from 1 to 5 times. **c** Micrographs of *Arabidopsis* epidermis inoculated with *Ctro* Ishigaki-Banana2 at 1 and 3 dpi in the presence or absence of MBIs. Black and white arrowheads indicate melanised and nonmelanised appressoria, respectively. Black and white arrows indicate the elongated germ tube-like and invasive hyphae, respectively. Asterisks indicate conidia. Scale bar = 10 µm. **d** Quantification of MAE- and HTE-related morphogenesis of *Ctro* Ishigaki-Banana2 on *Arabidopsis* triple mutants, with or without MBIs. After 16 h of incubation, the proportion of the conidia exhibiting melanised (MA) and nonmelanised (NMA) appressorium formation, hyphal extension (HE), or no germination (NG) was quantified. N.D. indicates not determined owing to the formation of a short germ tube whose subsequent morphogenesis is unpredictable. At least 290 conidia were investigated. The mean and SE were calculated from five (DW, CAR, PRQ) or four (FTL, TPC) independent experiments. **e** Quantification of MAE- and HTE-related morphogenesis of *Ctro* Ishigaki-Banana2 in the presence or absence of sugar (Glc). After 16 h of incubation, the proportion of the conidia exhibiting MA, HE, or NG was quantified. N.D. indicates not determined. At least 100 conidia were investigated. The mean and SE were calculated from three independent plants. **f** Pathogenicity of *Ctro* Ishigaki-Banana2 on host plants. The inoculated ripe fruits of the Banana cv. ‘Gros Michel’ were incubated for 2 d. When necessary, MBIs were added. **g** Micrographs of banana epidermis inoculated with *Ctro* Ishigaki-Banana2 at 1 dpi with or without MBIs. Similar HTE-type morphogenesis was observed in three independent experiments. Black arrowhead and arrows indicate the melanised appressorium and elongated germ tube-like hyphae. Asterisks indicate conidia. Scale bar = 10 µm.

In contrast to MBI-D^31–33^, *P. oryzae* strains resistant to MBI-R fungicides have not emerged, despite several decades of continuous use^57^. Therefore, the potential risk for resistance development to MBI-R fungicides is estimated to be low. Intriguingly, we found that *Ctro* Ishigaki-Banana2 exhibited specific insensitiveness to MBI-R pyroquilon in the lesion production on *A. thaliana* immunocompromised mutants, whereas other MBIs, such as carpropamid and tolprocarb, remained effective (Fig. 8a). Another MBI-R, fthalide, strongly prevented the *Ctro* Ishigaki-Banana2-induced lesion formation in *A. thaliana*, in contrast with the dose-dependent lesion-producing effects of pyroquilon (Fig. 8b). Specifically, in the presence of carpropamid or tolprocarb, appressoria formed on the plant surface were arrested at the premelanisation stage and could not penetrate plant epidermis (Fig. 8c). Therefore, *Ctro* Ishigaki-Banana2 was classified as a conventional MAE-type pathogen (Fig. 4b). In contrast, *Ctro* Ishigaki-Banana2 unexpectedly exhibited suppressed appressorial formation, germ tube-like hyphal extension and HTE-based invasive hyphal development in the presence of pyroquilon, while melanisation of partly formed appressoria was sufficiently prevented (Fig. 8c, d). Therefore, *Ctro* Ishigaki-Banana2 was characterised as pyroquilon-induced HTE-type (Fig. 4b). Moreover, this pathogen was classified as a putative sugar-induced HTE-type strain (Fig. 8e). These phenomena indicate that, in addition to carbohydrates, specific agrochemicals may act as factors that induce appressorium-independent, HTE-related morphogenesis and the subsequent plant invasion via HTE by specific *Colletotrichum* strains, although MBIs are not applied to crops affected by *Colletotrichum*. On the contrary, the virulence of *Ctro* Ishigaki-Banana2 on ripe fruits of its host banana was not affected by treatments with any of the investigated MBIs (Fig. 8f). *Ctro* Ishigaki-Banana2 consistently exhibited HTE-type morphogenesis on the host surface regardless of the presence or absence of MBIs, suggesting host infection via HTE induced by sugar and/or other ripe fruit-derived signal(s) (Fig. 8g).

## Discussion

Many fungal phytopathogens produce an appressorium for host penetration^1^, and conventional MAE-based plant invasion by the destructive pathogens *Colletotrichum* spp. and *P. oryzae*^1,3,4^ has been extensively studied. The excellent agrochemical efficacy of MBIs^7–9^ against these pathogens has also been well-established for decades, stemming from the discovery of a close relationship between appressorial melanisation and penetration^16^. Plant invasion and lesion formation by these fungi have not occurred consistently via morphologically mature, unicellular nonmelanised appressorium^16,17,20–30,54^.

In the present study, we discovered *Colletotrichum* fungi with plant invasion capabilities via morphologically mature, nonmelanised unicellular appressoria (Supplementary Fig. 16). This entry mode, designated as NMAE, confers inherent insensitiveness to MBIs during appressorium-based plant invasion and lesion formation by achieving near-complete appressorial functionality well before the melanisation stage (Supplementary Figs. 8 and 16). The NMAE-type strains all belonged to the acutatum SC, a monophyletic group in the phylogenetic tree of the genus *Colletotrichum*, suggesting that the NMAE trait evolved near the base of this SC (Fig. 4b). However, two singleton species were identified, *Colletotrichum pseudoacutatum* and *Colletotrichum orchidophilum*, between the trichellum SC and acutatum SC divergences^10^. To accurately determine the point at which an ancestral NMAE-type pathogen emerged, analysing these singleton species was necessary. In practical settings, MBIs have been exclusively applied to rice paddies to control blast diseases caused by *P. oryzae* since the 1980s^7–9^. In contrast, MBIs are not used for anthracnose diseases caused by *Colletotrichum* species; therefore, the application of MBIs in the field does not exert any selection pressure on *Colletotrichum* species. Most importantly, the acutatum SC emerged over 9 million years ago^58^; hence, the NMAE-based insensitiveness of *Colletotrichum* fungi to MBIs was acquired incidentally.

In a recent study, Kumakura et al. demonstrated that, instead of the melanin biosynthesis gene *PKS1*, DHHA polymer biosynthesis genes *PKS2* and *PBG13* conserved across multiple fungal species in Ascomycota, directly reduce appressorial cell wall porosity in *Colletotrichum* fungi. These findings suggest that melanisation is not primarily required for turgor generation^15^. Notably, the Ascomycota fungus *Botrytis cinerea* produces a slightly swollen, nonmelanised germ tube apex called a protoappressorium and penetrates plants with turgor generation^59^; this is consistent with the presence of the *PKS2* and *PBG13* gene pair in *B. cinerea*^15^. Instead, Kumakura et al. indicated that melanin accumulation appeared to increase the physical rigidity of the appressorial cell wall, as evidenced by reduced stiffness in the Δ*pks1* mutant of *Corb*^15^. Notably, in our turgor evaluation assays, *Corb* 104-T exhibited a higher rate of collapsed or cavitated appressoria than other *Colletotrichum* fungi, including MAE- and NMAE-type strains, likely owing to low cell wall rigidity (Fig. 3a, c). Another fungal phytopathogen, *Phakopsora pachyrhizi*, develops a nonmelanised appressorium but generates high turgor pressure in a melanin-independent manner^60^. However, we found that the genome of *P. pachyrhizi* likely lacked the *PKS2* and *PBG13* gene pair as in the case with other Basidiomycota fungi^15^, implying an alternative mechanism for turgor generation. Nevertheless, melanisation remains essential for appressorium-mediated plant invasion of *Colletotrichum* fungi except for NMAE-type strains (Fig. 4). The penetration ability of *Corb* into cellulose membrane occurs independently of turgor but depends on melanisation^15^, highlighting the importance of concerted invasion strategies employing various melanisation-dependent and -independent appressorial functions. While the degree of dependence in each function varies among *Colletotrichum* strains, NMAE-type strains in acutatum SC may achieve almost full functionality for appressorial penetration (Supplementary Fig. 8). Alternatively, acutatum SC may qualitatively rewire infection-related developmental programmes compared to conventional *Colletotrichum* fungi. Consistent with this idea, acutatum SC and its closely-related species *C. orchidophilum* lack the tandem Perox–AlcOx metalloenzyme system that chemically remodels host cuticular components into signalling molecules required for proper appressorium development^61^. However, except for appressorial pigmentation, MAE- and NMAE-type strains do not exhibit evident morphological differences.

Compared with conventional MAE, NMAE shortens the time required for plant penetration and likely facilitates early plant infection with *Colletotrichum* fungi (Fig. 6 and Supplementary Fig. 16). Indeed, the appressoria of *Cnym* PL1-1-b were gradually pigmented with melanin over time but exhibited early plant invasion and infection via NMAE at the premelanisation stage under MBI-free conditions (Fig. 5a, c, d). Notably, in the *Colletotrichum* phylogenetic tree, the acutatum SC is a relatively recently diverged phylogenetic group^58^, in which more than 50 species have already been described in diverse hosts^10,56^. This rapid expansion of the acutatum SC might be attributed to an unconventional early entry mode, such as NMAE. In *C. orbiculare*, a model MAE-type pathogen, appressorium morphogenesis is tightly integrated with chemical surface sensing, polarity establishment, and cell-cycle control. This integration is mediated through conserved pathways, such as the MOR/NDR kinase cascade^62^ and the Bub2–Bfa1–Tem1 module, which regulates G1/S progression during infection-related morphogenesis^63^. In contrast, the alternative entry modes of the acutatum SC, including NMAE and HTE, combined with the absence of chemical gating mechanisms (e.g., Perox–AlcOx), suggest that these fungi may employ alternative combinations of morphogenetic timing, polarity control, and host surface engagement to achieve rapid and effective penetration.

Except for *Cfio* HF3, most NMAE-type *Colletotrichum* strains in the acutatum SC still exhibited definitive appressorial melanisation (Figs. 1d and 5, and Supplementary Fig. 10). Given the polyphagous behaviour of acutatum SC^56^ and the noncommittal requirement of melanisation ability in lesion formation among plant species (Figs. 1, 2, and Supplementary Figs. 14, 15), NMAE-type fungi may retain appressorial melanisation for the infection of specific plants that develop effective preinvasive resistance against NMAE but allow MAE. We confirmed the occurrence of NMAE resistance regardless of the type of monocot or dicot herbaceous plant; cockscomb, maize, and sorghum prevented lesion formation by NMAE-type *Cfio* CC1 Δ*scd1*, whereas cosmos, spinach, and rice did not (Fig. 2 and Supplementary Fig. 14). The same is true of woody plants; Japanese andromeda prevented lesion formations of NMAE-type pathogens, whereas sacred bamboo and Japanese summer orange did not (Supplementary Fig. 15). The plant immune factor(s) that cause these differences in preinvasive resistance against NMAE remain to be elucidated. Nonmelanised appressoria of *Cfio* CC1 Δ*scd1* still maintained almost all the invasion-linked functions analysed in the present study (Supplementary Fig. 8); therefore, the unknown appressorial function(s) responsible for the ability to overcome the preinvasive defence(s) of NMAE-resistant plants in a melanisation-dependent manner also remain to be explored. Alternatively, successful appressorium-mediated entry into plants with erratic appressorial melanisation by *Cfio* HF3 can be regarded as a drastic evolution, where the pathogen eliminated melanisation dependency for plant invasion.

In addition to NMAE, we rediscovered several HTE-type *Colletotrichum* fungi with early invasion capabilities in the gloeosporioides and acutatum SCs (Fig. 4b and Supplementary Fig. 16). The gloeosporioides SC is also a relatively recently diverged group^58^ and, similar to the acutatum SC, exhibits expansion with many polyphagous strains and diverse host plants in the genus *Colletotrichum*^10,56^. Early plant invasion and/or the use of distinct entry modes according to the environment on the plant surface might be beneficial for survival. While HTE is occasionally accompanied by appressorium-like swelling at the elongated hyphal tip^34^, it exhibits more invasive penetration and increased lesion formation than MAE^34^ (Fig. 7 and Supplementary Fig. 16). HTE clearly differs from NMAE, whose entry rates are comparable to or lower than those of MAE (Figs. 1b, 2c, 5d and Supplementary Figs. 11b, 16). Whether HTE-type strains exist in SCs other than the gloeosporioides and acutatum SCs remains to be addressed. Given the distribution of HTE-type strains within these distantly related SCs, HTE and NMAE represent essentially distinct early entry modes for plant infection (Fig. 4b and Supplementary Fig. 16).

To date, *Colletotrichum* strains that can infect rice have not been reported; rather, some *Colletotrichum* strains have been evaluated as potential bioherbicides in rice fields^64,65^. In the present study, we found that *Cfio* CC1 could invade and develop visible lesions on rice leaves without appressorial melanisation (Supplementary Fig. 14e, f). However, the progression of faint lesions was restricted and assumed to be a hypersensitive-like response in rice (Supplementary Fig. 14e), which was consistent with the well-known incompatibility of *Colletotrichum* fungi with rice. Therefore, at present, it is unlikely that NMAE-type and polyphagous *Colletotrichum* strains in the acutatum SC will become fully adapted to rice and pose a serious threat.

The MBI-R compound, pyroquilon, induced appressorium-independent HTE-related morphogenesis, HTE, and consequent pyroquilon-insensitive lesion production on *Arabidopsis* in *Ctro* Ishigaki-Banana2 (Fig. 8). Therefore, we speculate that pyroquilon may increase fungal virulence by inducing a more invasive entry mode in the specific plant–fungus interaction. However, *Ctro* Ishigaki-Banana2 develops HTE-type morphogenesis and causes subsequent necrotic lesion formation on the host banana, irrespective of the MBI treatments (Fig. 8f, g). As MBIs are not used and do not exert selection pressure on *Colletotrichum* species in the field, pyroquilon-induced HTE might be incidentally acquired. Therefore, at present, no consideration is required for the adverse side effects of MBI-R on *Colletotrichum* infection strategies in agricultural practice. Although MAE-type *P. oryzae* also employs an appressorium-independent invasion strategy for rice root colonisation, where the penetration occurs via HTE-like hyphal swelling^66^, typical foliar infection is tightly linked to MAE-based invasion. This strong dependency explains why *P. oryzae* has not yet developed resistance to MBI-R fungicides^57^; there is no evidence to support NMAE-based invasion or MBI-induced HTE-like invasion in *Pyricularia* fungi to date.

In the present study, we propose a key paradigm for the *Colletotrichum* fungi plant infection strategy, which requires a perspective shift from the conventional MAE-based single-entry mode to a diversified entry mode comprising MAE, NMAE, and HTE (Supplementary Fig. 16). In the evolution of appressoria-producing phytopathogenic fungi, nonmelanised appressoria may be the ancestral form^1^. MAE-type *Colletotrichum* and *Pyricularia* fungi with strong reliance on melanisation diverged independently from ancestral fungi with nonmelanised appressoria. However, a certain group of *Colletotrichum* fungi, the acutatum SC, have evolved into NMAE-type pathogens, a process that appears morphologically regressive, yet confers the advantage of early plant invasion (Supplementary Fig. 16). The same may also be true for HTE-type pathogens, which exhibit similar morphology to protoappressorium. Given the rapid expansion of the acutatum and gloeosporioides SCs^10,56^, the emergence of NMAE- and HTE-type strains may be a turning point in the evolutionary history of *Colletotrichum* fungi.

## Methods

### Fungal strains and media

All *Colletotrichum* and *Pyricularia* strains, including their sources, used in this study are listed in Supplementary Data 1. Transgenic *Colletotrichum tropicale* strain S9275 expressing *mRFP*^34^ has been previously described. Cultures of all fungal isolates of *Colletotrichum* were maintained on 2.5 or 3.9% (w/v) PDA medium (Nissui Pharma or BD Difco) at 24 °C in the dark. Cultures of *Pyricularia oryzae* were maintained on oatmeal agar medium (BD Difco) at 24 °C in the dark. For conidiation, some fungi were cultured under a 16 h black light/8 h dark cycle.

### Plant lines and growth

*Arabidopsis thaliana* seeds were sown on rockwool (Grodan) and grown at 22 °C with 16 h of illumination per day in nutrient medium. *Arabidopsis thaliana* accession Col-0 was used as the wild-type. *pen2-1*^67^, *edr1-1 pen2-1*^55^, and *edr1-1 pen2-1 gsh1-1*^36^ mutants have been previously described. Seeds of cucumber (*Cucumis sativus* L. cv. Suyo), cosmos (*Cosmos bipinnatus* cv. dwarf early Vega), spinach (*Spinacia oleracea* L. cv. Nihon), cockscomb (*Celosia argentea* L. cv. Yachiyo Keitou), and rice (*Oryza sativa* cvs. Koshihikari, Milyang 23, and Silewah) were grown on soil and grown at 22 °C with 16 h of illumination per day. Saplings of apple tree (*Malus domestica* Borkh. cv. Kogyoku), Japanese ivy (*Hedera rhombea*), Japanese summer orange (*Citrus natsudaidai*), and hyacinth (*Hyacinthus orientalis*) were grown at 24 °C with 16 h of illumination per day. Trees of Japanese flowering cherry (*Cerasus* × *yedoensis* (syn. *Prunus* × *yedoensis*) cv. Somei-Yoshino and *Cerasus* × *subhirtella* (syn. *Prunus* × *subhirtella*) cv. Takato-kohigan), Sacred bamboo (*Nandina domestica* wild-type and cv. Otafukunanten), and Japanese andromeda (*Pieris japonica*) were grown in the field. Banana (*Musa acuminata* cv. Gros Michel and *Musa acuminata* × *balbisiana* cv. Cardaba) was used at the ripening stage of fruits.

### Genotyping of *Arabidopsis* mutants

Genetic cross of *pen2-1*^67^ and *ein2-1* (CS3071) was performed, then the *pen2-1 ein2-1* was crossed with *edr1-1 pen2-1 gsh1-1 eds5-1*^36^ to generate *edr1-1 pen2-1 gsh1-1 eds5-1 ein2-1*. The *edr1-1 pen2-1 gsh1-1 eds5-1 ein2-1* was crossed with *edr1-1 pen2-1 gsh1-1 eds5-1 cas chup1*^36^ to establish *edr1-1 pen2-1 gsh1-1 eds5-1 ein2-1 cas chup1*. The primers used for genotyping are listed in Supplementary Data 2. The *pen2-1*, *ein2-1*, *edr1-1*, *gsh1-1*, and *eds5-1* mutations were checked with the corresponding specific primers for the derived cleaved amplified polymorphic sequence (dCAPS) markers using dCAPS Finder 2.0 (http://helix.wustl.edu/dcaps/dcaps.html), and the PCR product types (wild-type or mutant) were cleaved with their corresponding restriction enzymes listed in Supplementary Data 2. Transfer DNA insertions into *CAS1* and *CHUP1* genes were checked using specific primers.

### Plasmid construction

The *Escherichia coli* strain DH5α was used as the host for DNA manipulation. All primers used for plasmid construction are listed in Supplementary Data 3. All plasmids are derivatives of pCB1636^68^ that carry the hygromycin phosphotransferase (*HPH*) gene to confer hygromycin resistance. To generate *SCD1* gene disruption vectors, the 2.1–3.0 kb upstream regions and the 3.0 kb downstream regions of the *SCD1* genes of *Chig* Abr1-5, *Corb* 104-T, *Csia* MAF1, and *Cfio* CC1 were amplified from the genome of the corresponding strain. For the upstream regions, the amplified *Xho*I-*Xho*I (*Chig* Abr1-5), *Apa*I-*Xho*I (*Corb* 104-T), *Kpn*I-*Kpn*I (*Csia* MAF1), and *Xho*I-*Xho*I (*Cfio* CC1) fragments, respectively, were digested with the indicated enzymes and introduced into the corresponding 5′-sites of *HPH*. For the downstream regions, the amplified *Eco*RV-*Xba*I (*Chig* Abr1-5), *Eco*RI-*Spe*I (*Corb* 104-T), *Hind*III-*Spe*I (*Csia* MAF1), and *Cla*I-*Spe*I (*Cfio* CC1) fragments, respectively, were digested with the indicated enzymes and introduced into the corresponding 3′-sites of *HPH*, resulting in pHYChigAbr1-5SCD1del, pHYCorb104-TSCD1del, pHYCsiaMAF1SCD1del, and pHYCfioCC1SCD1del, respectively.

### Fungal transformation and gene disruption

Protoplast preparation and transformation were performed as previously described^69^. In brief, to obtain protoplasts, a cultured mycelium was harvested and treated with an enzyme solution containing 10 mg/mL of Driselase (Sigma-Aldrich) and 10 mg/mL of lysing enzymes (LEs) from *Trichoderma harzianum* (Sigma-Aldrich) in 1.2 M MgSO_4_ and 10 mM Na_2_HPO_4_ for 1–3.5 h. For transformation, the mixture of protoplasts and plasmid DNA was incubated on ice, and then gradually treated with 40% polyethylene glycol (PEG) solution at room temperature. After the removal of the PEG solution, protoplasts were poured onto a selection plate. Targeted gene disruption of *SCD1* was performed via homologous recombination in each *Colletotrichum* strain using the corresponding gene disruption vector (Supplementary Fig. 4). Hygromycin-resistant transformants were selected on plates containing 100 µg/mL of hygromycin B (Wako Pure Chemicals). To confirm *SCD1* gene disruptions, genomic PCR analysis was performed using two primers containing the border sequences of *SCD1* of each fungal strain.

### Fungal inoculation

For the inoculation assay of *Colletotrichum* fungi on plants, a conidial suspension of each *Colletotrichum* fungus (5 × 10^5^ conidia/mL) was drop-inoculated onto leaves, cotyledons, leaf sheaths, petals, or fruits of plants and incubated at 22 °C. For inoculation on leaves of apple and Japanese summer orange, a conidial suspension of each *Colletotrichum* fungus (1.0 × 10^6^ conidia/mL) was prepared. For inoculation on leaves of sacred bamboo and Japanese andromeda, a conidial suspension of each *Colletotrichum* fungus (2.0 × 10^6^ conidia/mL) was prepared. For analyses of appressorium melanisation and penetration, turgor pressure, and LE resistance, a conidial suspension (5 × 10^5^ conidia/mL 0.1% yeast extract solution) was placed on a glass slide or cellulose membrane and incubated at 24 °C. The 0.1% yeast extract solution was removed and replaced with water at 15 min after incubation. For *P. oryzae* inoculation, a conidial suspension (5 × 10^5^ conidia/mL) was placed on a coverslip and incubated for 60 min at 24 °C. After incubation, the conidia were washed and resuspended in water. When necessary, 0.5% glucose and the MBIs, 10 μg/mL carpropamid, 100 μg/mL pyroquilon, 50 μg/mL tolprocarb, and 200 μg/mL fthalide were added. In the dose-dependent assay, 100, 200, or 500 μg/mL pyroquilon were added.

### Microscopy

To measure the rates of fungal entry into the plant epidermis and the cellulose membrane, the inoculated cotyledons, leaf sheaths, and cellulose membranes with fungal cultures were mounted in water under a coverslip, with the inoculated surface facing the objective lens. The samples were observed using an Axio Imager. A2 microscope (Zeiss) equipped with an EC Plan-Neofluar 100x (1.30 numerical aperture) oil-immersion objective. The MAE and NMAE rates (%) were calculated using the following numerical formula: (the number of appressoria with formation of invasive hypha)/(the number of appressoria) × 100. To quantify the MAE- and HTE-related morphogenesis, after incubation on the plants, samples were observed using an Axio Imager. A2 microscope (Zeiss) equipped with an EC Plan-Neofluar 100x (1.30 numerical aperture) oil-immersion objective. The proportion of appressorium formation, germ tube-like hyphal extension, short germ tube formation, and no germination were then quantified using the following numerical formula: (the number of conidia forming appressorium)/(the number of conidia) × 100, (the number of conidia with hyphal extension)/(the number of conidia) × 100, (the number of conidia with short germ tube)/(the number of conidia) × 100, (the number of nongerminated conidia)/(the number of conidia) × 100. For observation of HTE into the banana epidermis by *Cmus* Noudai-Netsu-Saku-Hogo-28(3), the samples were observed using an all-in-one fluorescence microscope BZ-X800 (KEYENCE) equipped with a Plan Apochromat 100x (1.45 numerical aperture) oil-immersion objective. To evaluate the appressorial turgor pressure, after incubation on the glass surface for 24 h (*Colletotrichum* strains) or 20 h (*P. oryzae*), the appressoria were exposed to a range of PEG8000 or PEG6000 concentrations for 10 min. The samples were then observed using an Axio Imager. A2 microscope (Zeiss) equipped with an EC Plan-Neofluar 100x (1.30 numerical aperture) oil-immersion objective. The proportion of appressoria collapsed by cytorrhysis or cavitation was then quantified using the following numerical formula: (the number of collapsed appressoria)/(the number of appressoria) × 100. To assess the durability of appressoria on the plant surface, the samples were observed at 1 dpi and evaluated using the following numerical formula: (the number of appressoria with normal shape)/(the number of appressoria) × 100. For visualisation of the cell wall components and cytoplasmic lipid bodies in the analysis of LE resistance, samples incubated on the glass surface for 24 h were treated with LEs, then stained using 0.1 mM Calcofluor White M2R solution (Cosmo Bio Co.) for 10 min and Nile red (FUJIFILM Wako Pure Chemical Co.) for 5 min, respectively. Stained samples were washed two times with DW and observed using confocal laser-scanning microscopy. To analyse the ECR, the inoculated cotyledons were observed using confocal laser-scanning microscopy. Fluorescence was detected using an IX81 confocal microscope (Olympus) equipped with a diode laser (405/473/635 nm), LDD559 laser (559 nm), and a 60x UPlanSApo (1.35 numerical aperture) oil-immersion objective. Images were acquired and processed using FLUOVIEW FV1000-D (Olympus), GIMP, and ImageJ (rsb.info.nih.gov/ij/). To detect papillary callose deposition in *Arabidopsis* epidermal cells, a DAPI filter was used. To detect the signals of the cell wall components, cytoplasmic lipid bodies, and chlorophyll, fluorescence filters for DAPI, PI, and Cy5 were used, respectively. The dichroic mirror, beam splitter, and emission filter sets were DM405/473/559, SDM560, and BA425–475 for DAPI and BA570–670 for PI, and DM405/473/559/635, SDM560, and BA650–750 for Chlorophyll. The LE resistance rate (%) was evaluated using the following numerical formula: (the number of appressoria with normal shape and cytoplasmic lipid bodies)/(the number of appressoria) × 100. The ECR rate (%) was calculated using the following numerical formula: (the number of epidermal cells with surface chloroplasts)/(the number of epidermal cells that contact the melanised appressorium) × 100.

### Statistical analysis

Statistical analyses were performed using R version 3.5.2. Levene’s test was applied to check for heteroscedasticity between the treatment groups. To examine the differences among experimental groups, data were analysed with Tukey’s honestly significant difference and Dunnett’s test, as appropriate. When necessary, the unpaired *t*-test (two-tailed) was used. Differences were considered significant at *P* < 0.05.

### Reporting summary

Further information on research design is available in the Nature Portfolio Reporting Summary linked to this article.

## Supplementary information

Supplementary Information

Descriptions of Additional Supplementary Files

Supplementary Data 1

Supplementary Data 2

Supplementary Data 3

Reporting Summary

Transparent Peer Review file

## Source data

Source data

## Data Availability

All data presented in graphs and unprocessed gel images are available in the Source Data file of this paper. Other data are available upon request to the lead contact author. Source data are provided with this paper.
