# Supplementary material for: Breaking dependence on melanisation imparts diversity to a dogmatic invasion strategy of phytopathogenic fungi
Source: Nat Commun. 2026 Jun 27;17:6126. doi: 10.1038/s41467-026-74937-6 (PMC13365536; doi:10.1038/s41467-026-74937-6)
Supplement: Supplementary file 1 — Supplementary Information [file 41467_2026_74937_MOESM1_ESM.pdf]

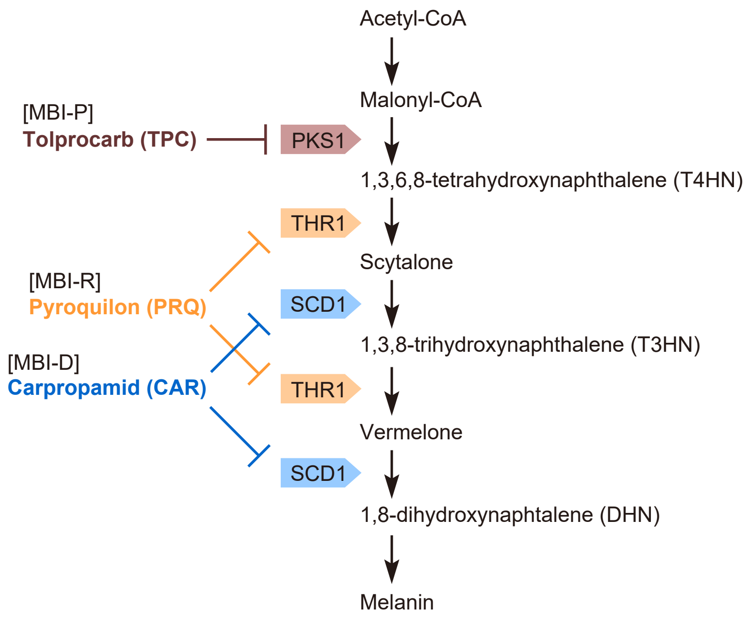

Supplementary Fig. 1. Fungal melanin biosynthesis pathway and inhibitors.

The melanin biosynthesis inhibitors (MBIs) carpropamid (CAR), pyroquilon (PRQ), and tolprocarb (TPC) are effective agrochemicals. MBI-D CAR, MBI-R PRQ, and MBI-P TPC inhibit scytalone dehydratase (SCD1), tetrahydroxynaphthalene reductase (THR1), and polyketide synthase (PKS1), respectively, in the fungal melanin biosynthesis pathway.

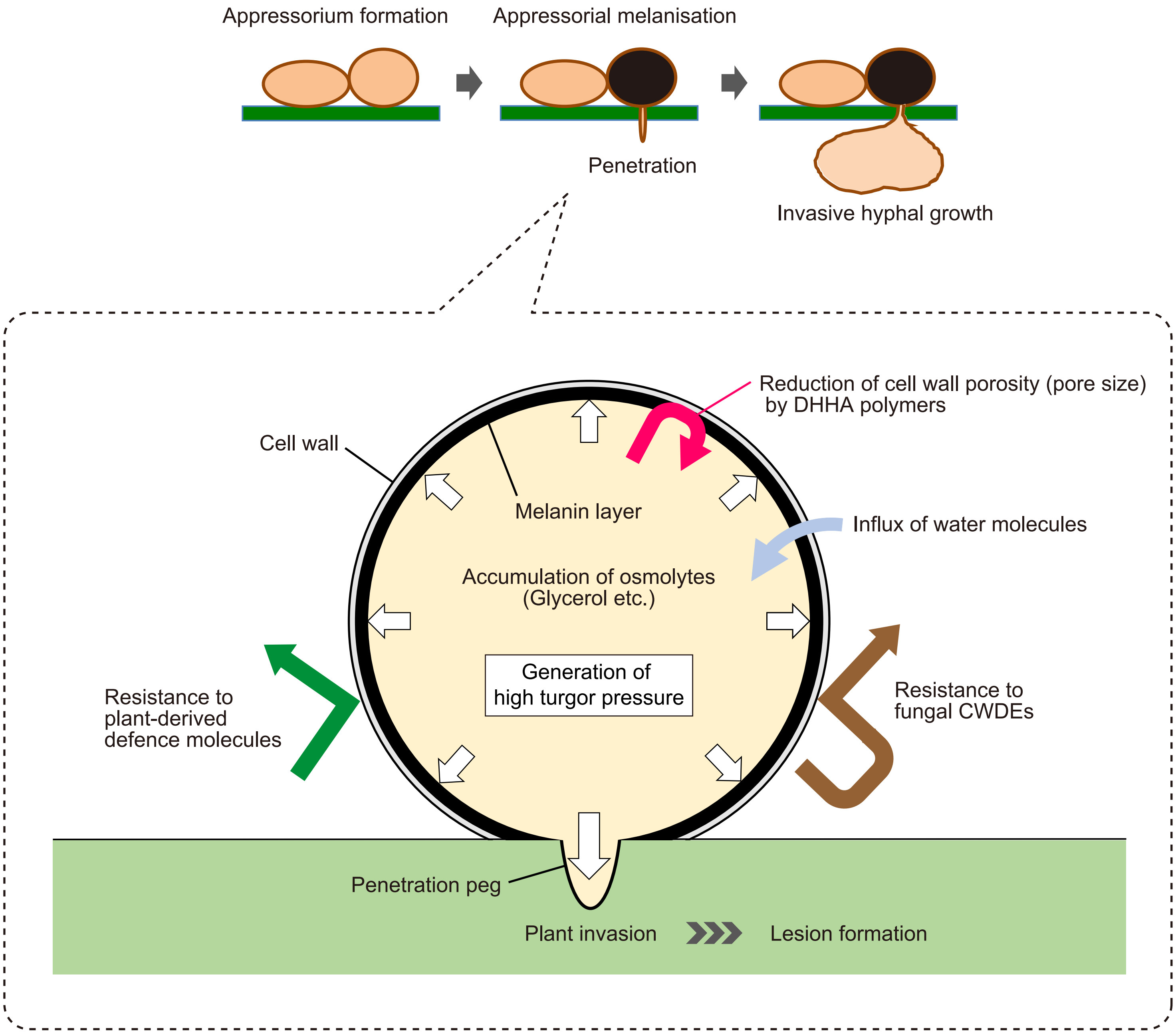

Supplementary Fig. 2. Appressorial functionality and melanisation dependencies in *Colletotrichum* and *Pyricularia* fungi.

Upper: *Colletotrichum* and *Pyricularia* fungi commonly invade the plant epidermis via melanised appressorium-mediated entry (MAE). The melanisation of appressoria is essential for successful MAE into plants. **Lower:** A melanin layer is formed in the appressorial cell wall and contributes to various appressorial functions, including the generation of high turgor pressure, proper positioning of penetration pores, and maintenance of cell wall rigidity against fungal cell wall-degrading enzymes (CWDEs) and plant digestive enzymes. Finally, the melanised appressorium acquires the capability for plant invasion and lesion formation.

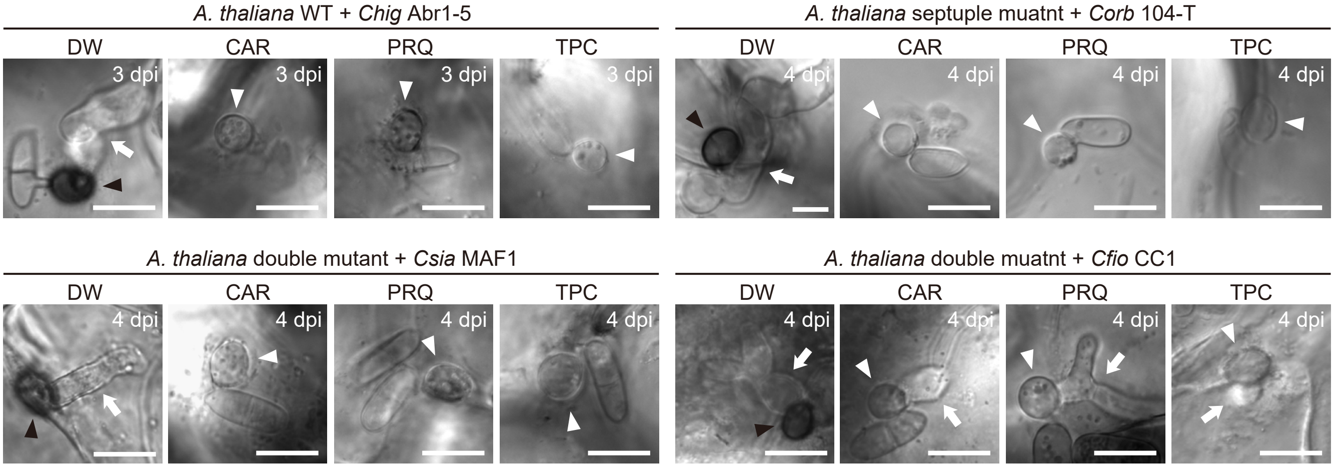

Supplementary Fig. 3. Melanin biosynthesis inhibitors do not prevent the invasion of *C. fioriniae* CC1 into *Arabidopsis thaliana* immunocompromised mutants.

Fungal invasion of *Arabidopsis* epidermis. A conidial suspension of *Chig* Abr1-5, *Corb* 104-T, *Csia* MAF1, and *Cfio* CC1 was inoculated onto cotyledons of *A. thaliana* wild-type, double (*edr1 pen2*), or septuple (*edr1 pen2 gsh1 eds5 ein2 cas chup1*) immunocompromised mutants with or without the melanin biosynthesis inhibitors carpropamid (CAR), pyroquilon (PRQ), and tolprocarb (TPC), and incubated for 4 d. Only the inoculum of *Chig* Abr1-5 was analysed at 3 d post-inoculation (dpi). Arrowheads indicate melanised (black) and nonmelanised (white) appressoria. White arrows indicate invasive hyphae. Scale bar = 10 µm.

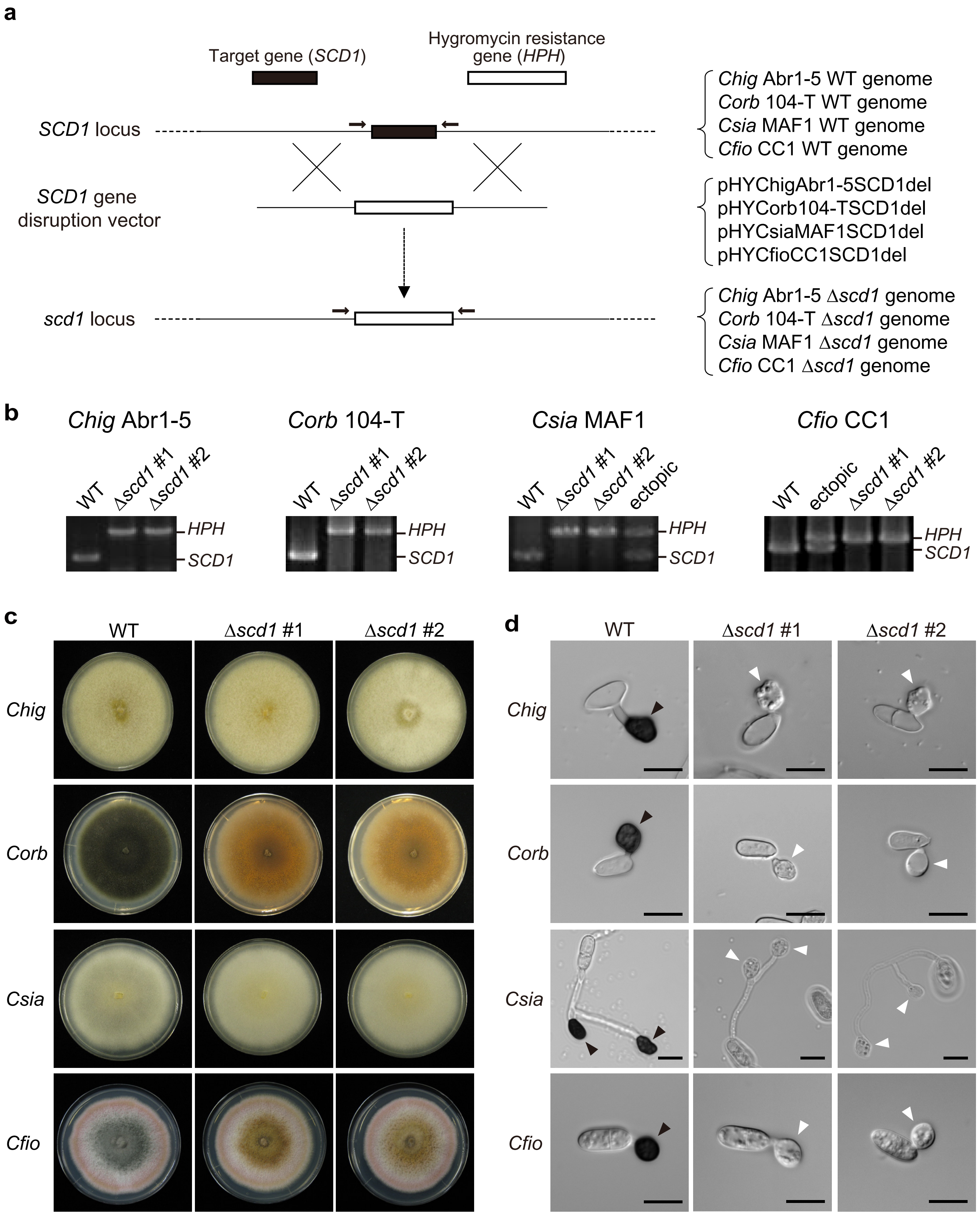

a
Target gene (SCD1)
Hygromycin resistance gene (HPH)
SCD1 locus
SCD1 gene disruption vector
scd1 locus
Chig Abr1-5 WT genome
Corb 104-T WT genome
Csia MAF1 WT genome
Cfio CC1 WT genome
pHYChigAbr1-5SCD1del
pHYCorb104-TSCD1del
pHYCsiaMAF1SCD1del
pHYCfioCC1SCD1del
Chig Abr1-5 Δscd1 genome
Corb 104-T Δscd1 genome
Csia MAF1 Δscd1 genome
Cfio CC1 Δscd1 genome
b
Chig Abr1-5
WT
Δscd1 #1
Δscd1 #2
HPH
SCD1
Corb 104-T
WT
Δscd1 #1
Δscd1 #2
HPH
SCD1
Csia MAF1
WT
Δscd1 #1
Δscd1 #2
ectopic
HPH
SCD1
Cfio CC1
WT
ectopic
Δscd1 #1
Δscd1 #2
HPH
SCD1
c
WT
Δscd1 #1
Δscd1 #2
Chig
Corb
Csia
Cfio
d
WT
Δscd1 #1
Δscd1 #2
Chig
Corb
Csia
Cfio

Supplementary Fig. 4. Generation of *SCD1* gene disruption mutants in the four *Colletotrichum* strains.

a Targeted gene disruption of *SCD1*. The *SCD1* locus and *SCD1* gene disruption vector contained a hygromycin phosphotransferase gene (*HPH*) cassette flanked by the border sequences of *SCD1* in *Chig* Abr1-5, *Corb* 104-T, *Csia* MAF1, and *Cfio* CC1. The primers used for PCR of the border sequences in each fungal strain were designed based on the genome data of *Chig* Abr1-5, *Corb*104-T, *Csia* Cg363, and *Cfio* PJ7 (Supplementary Data 3). The primers used for genomic PCR are indicated by arrowheads (Supplementary Data 3). **b** Genomic PCR analysis was performed using DNA isolated from the wild-type, Δ*scd1* mutants, and ectopic transformants of each fungal strain. Sequencing analysis revealed that the 5′ flanking region of *SCD1* in *Cfio* CC1 included a 452 bp insertion compared with that in *Cfio* PJ7; therefore, a slightly larger DNA fragment was detected in genomic PCR than in others. **c** Colony phenotypes of Δ*scd1* mutants on PDA agar in each fungal strain. Images were obtained after 8 (*Chig* Abr1-5), 14 (*Corb* 104-T), 14 (*Csia* MAF1), and 10 (*Cfio* CC1) d of incubation. Δ*scd1* mutants exhibited a loss of hyphal melanisation compared with the wild-type, except *Chig* Abr1-5, the wild-type of which did not demonstrate hyphal melanisation under the present growth conditions. **d** Appressorial phenotype of the Δ*scd1* mutants of each fungal strain. Conidial suspensions (5×10^5 conidia/mL) were incubated on glass surfaces for 24 h. Δ*scd1* mutants exhibited a loss of appressorial melanisation compared with the wild-type. Arrowheads indicate melanised (black) and nonmelanised (white) appressoria. Scale bar = 10 μm.

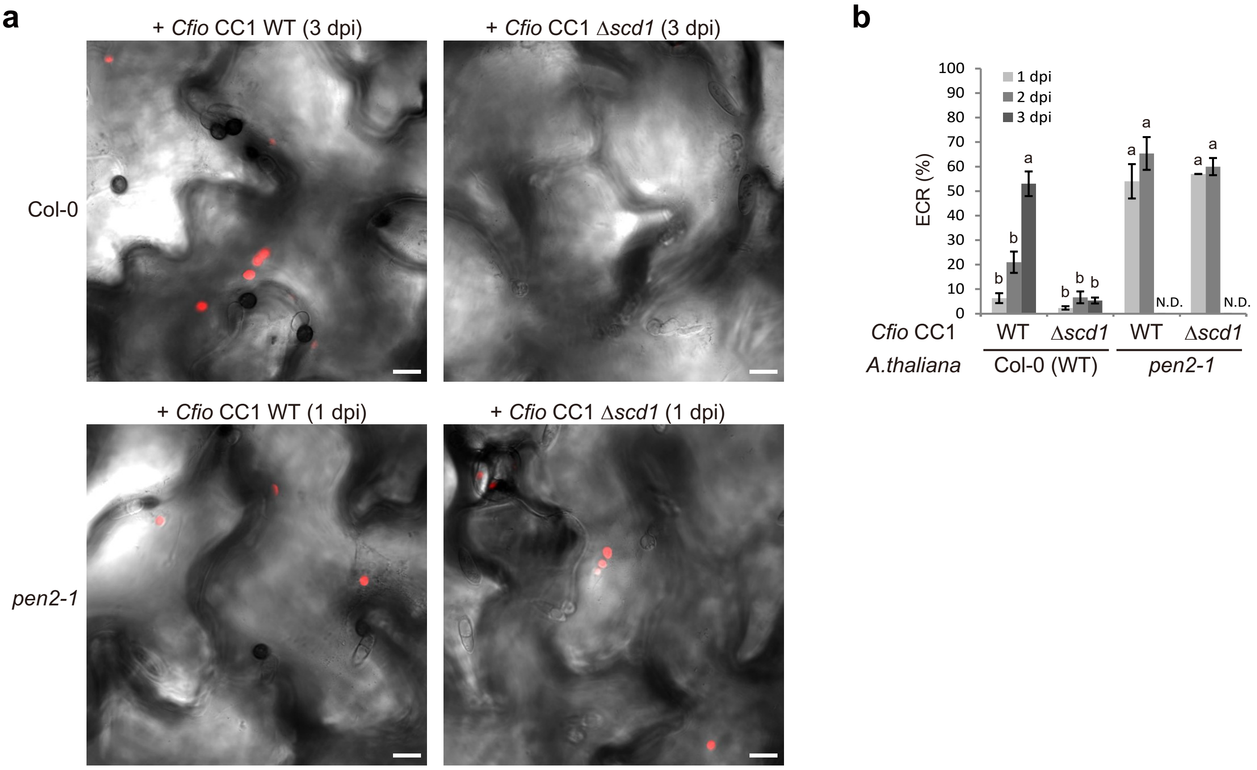

Supplementary Fig. 5. ***Arabidopsis*** **epidermal chloroplast response against** ***C. fioriniae*** **CC1 is attenuated by an appressorial melanisation defect.**

a Epidermal chloroplast response (ECR) in *A. thaliana* after inoculation with *Cfio* CC1 wild-type and Δ*scd1* mutants. The epidermal surfaces of *Cfio*-inoculated cotyledons of Col-0 and *pen2-1* mutants were investigated at 3 and 1 d post-inoculation (dpi), respectively. Chloroplasts were visualised using chlorophyll autofluorescence. DIC images were captured using confocal microscopy. Scale bar = 10 µm. **b** The ratio of epidermal cells with surface chloroplasts was investigated at 1, 2, and 3 dpi. A total of 100 cells in contact with the appressorium were observed. N.D.: not determined owing to damage of the epidermal cells by fungal invasion. The mean and SE were calculated from three independent plants. Means with different letters significantly differ ($P < 0.05$, two-way analysis of variance with Tukey's HSD).

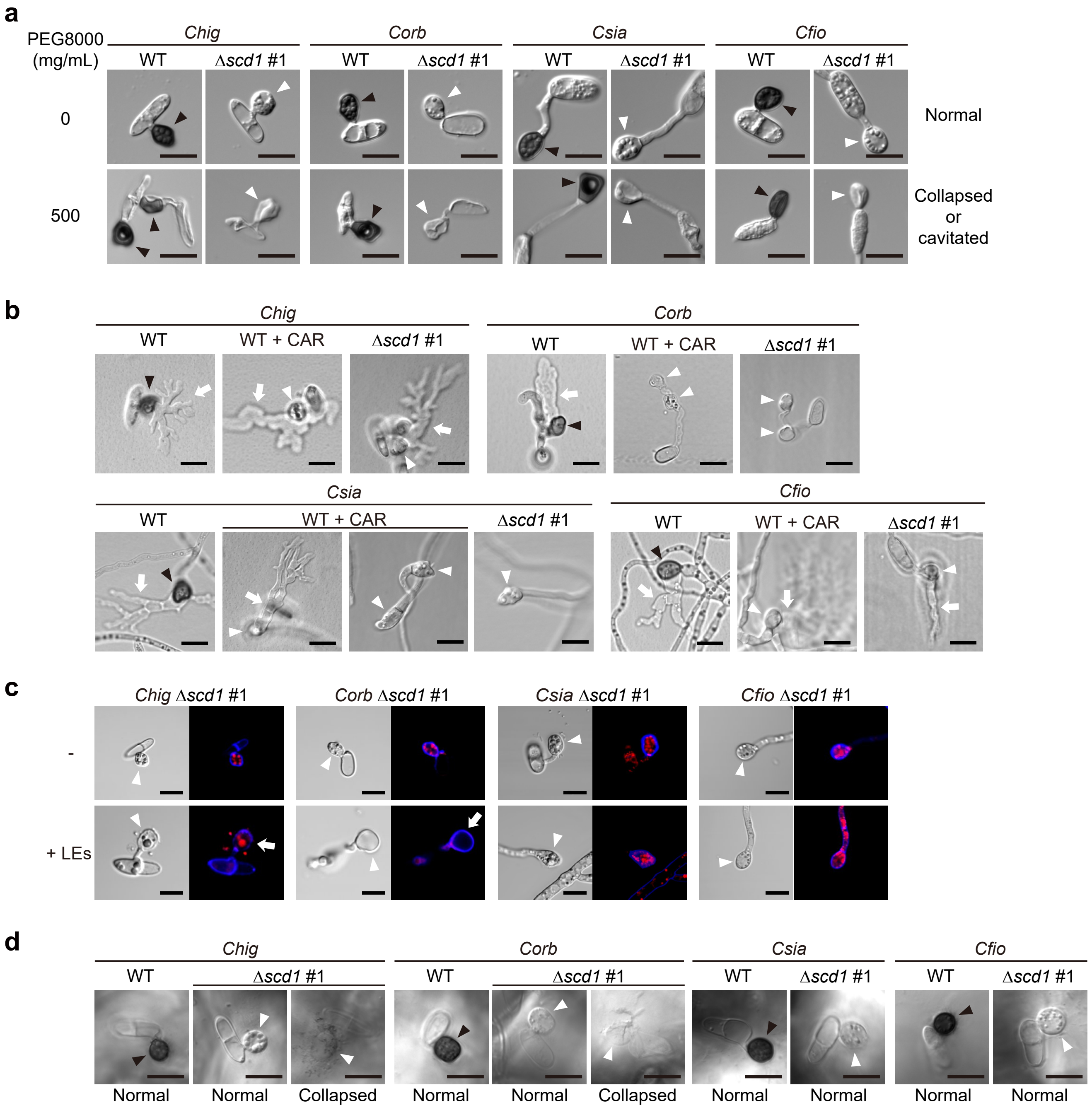

a
PEG8000 (mg/mL)
Chig
Corb
Csia
Cfio
WT
Δscd1 #1
0
500
Normal
Collapsed or cavitated
b
WT + CAR
c
Chig Δscd1 #1
Corb Δscd1 #1
Csia Δscd1 #1
Cfio Δscd1 #1
-
+ LEs
d
Normal
Collapsed

Supplementary Fig. 6. Melanisation dependence of each appressorial function varies among *Colletotrichum* fungi.

a Appressorial turgor was evaluated to assess the frequency of collapse or cavitation of appressoria in wild-type and Δ*scd1* mutants of *Chig* Abr1-5, *Corb* 104-T, *Csia* MAF1, and *Cfio* CC1 on a glass surface after exposure to a range of PEG8000 concentrations. The images are representative of normal (0 mg/mL PEG8000) and collapsed/cavitated (500 mg/mL PEG8000) appressoria. Arrowheads indicate melanised (black) and nonmelanised (white) appressoria. Scale bar = 10 µm. **b** Fungal entry into the artificial substratum cellulose membrane. A conidial suspension of each fungus was incubated on cellophane for 24 (*Chig* Abr1-5 and *Corb* 104-T) or 48 h (*Csia* MAF1 and *Cfio* CC1). When necessary, carpropamid (CAR) was added. The nonmelanised appressoria of *Corb* 104-T frequently formed secondary appressoria. *Csia* MAF1 occasionally formed abnormally-shaped appressoria on cellophane in melanisation inhibition conditions. Arrowheads indicate melanised (black) and nonmelanised (white) appressoria. White arrows indicate pseudoinvasive hyphae. Scale bar = 10 µm. **c** Integrity of the appressorial cell wall against cell wall-degrading enzymes. Morphologically mature appressoria of each fungus were incubated with lysing enzymes (LEs) from *Trichoderma harzianum*. The images are representative of nonmelanised appressoria of Δ*scd1* mutants in the presence or absence of LEs. Cytoplasmic lipid bodies stained with Nile Red were released when the appressorium raptured. Fungal cell walls were stained with Calcofluor white M2R. Arrowheads indicate nonmelanised appressoria. Arrows indicate collapsed appressoria. Scale bar = 10 µm. **d** Durability of appressoria on plants. A conidial suspension of each fungus was inoculated onto *A. thaliana* Col-0. Images are representative of normal and collapsed appressoria. Arrowheads indicate melanised (black) and nonmelanised (white) appressoria. Scale bar = 10 µm.

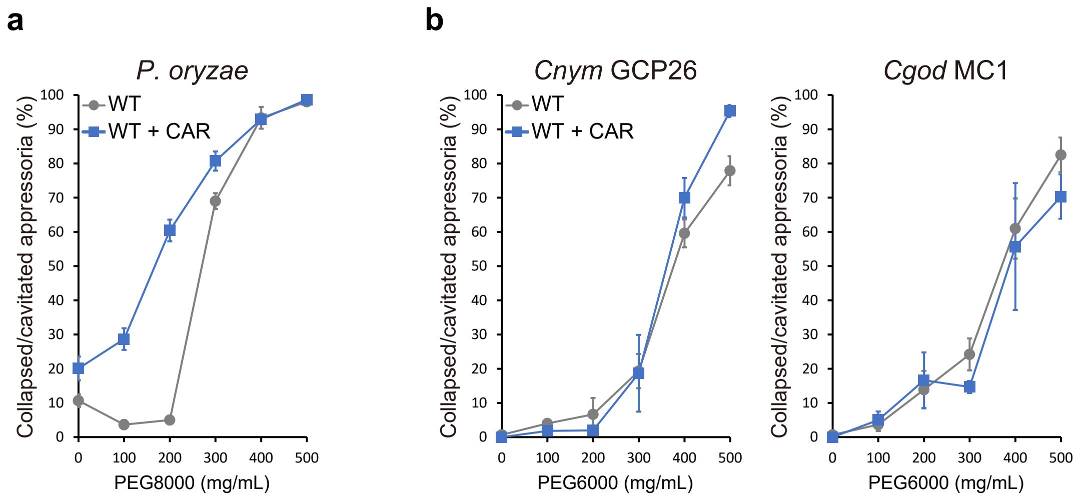

Supplementary Fig. 7. Appressorial melanisation and turgor generation in *Colletotrichum* strains and *Pyricularia oryzae*.

Turgor was evaluated to assess the frequency of appressorial collapse or cavitation. **a** The appressoria of *P. oryzae* Ina86-137 on glass surfaces were exposed to a range of PEG8000 concentrations. When necessary, the melanin biosynthesis inhibitor, carpropamid (CAR), was added. At least 85 appressoria were investigated. The mean and SE were calculated from three independent samples. **b** The appressoria of *Cnym* GCP26 and *Cgod* MC1 on glass surfaces were exposed to a range of PEG6000 concentrations. When necessary, CAR was added. At least 50 appressoria were investigated. The mean and SE were calculated from three independent samples.

Appressorial function \ *Colletotrichum* strain	***Chig* Abr1-5**		***Corb* 104-T**		***Csia* MAF1**		***Cfio* CC1**	
	+ CAR	Δ*scd1*	+ CAR	Δ*scd1*	+ CAR	Δ*scd1*	+ CAR	Δ*scd1*
High turgor generation and cell wall rigidity	○	○	×	×	○	○	○	○
Penetration of artificial substratum	○	○	×	×	×	×	○	○
Resistance to exogenous CWDEs	×	×	×	×	○	○	○	○
Normal morphology of appressoria on plant	△	△	○	○	○	○	○	○
Plant invasions	×	×	×	×	×	×	○	○
Lesion formation on plants	×	×	×	×	×	×	○	○

Supplementary Fig. 8. Appressorial functionality and melanisation dependencies in *Colletotrichum* fungi.

Summary of appressorial functions and melanisation dependencies of the four *Colletotrichum* strains. *Corb* 104-T strongly requires melanisation for almost all appressorial functions. *Cfio* CC1 maintains all functions in nonmelanised appressoria and develops lesions via nonmelanised appressorium-mediated entry (NMAE). *Chig* Abr1-5, *Corb* 104-T, and *Csia* MAF1 did not invade and infect the plants via NMAE.

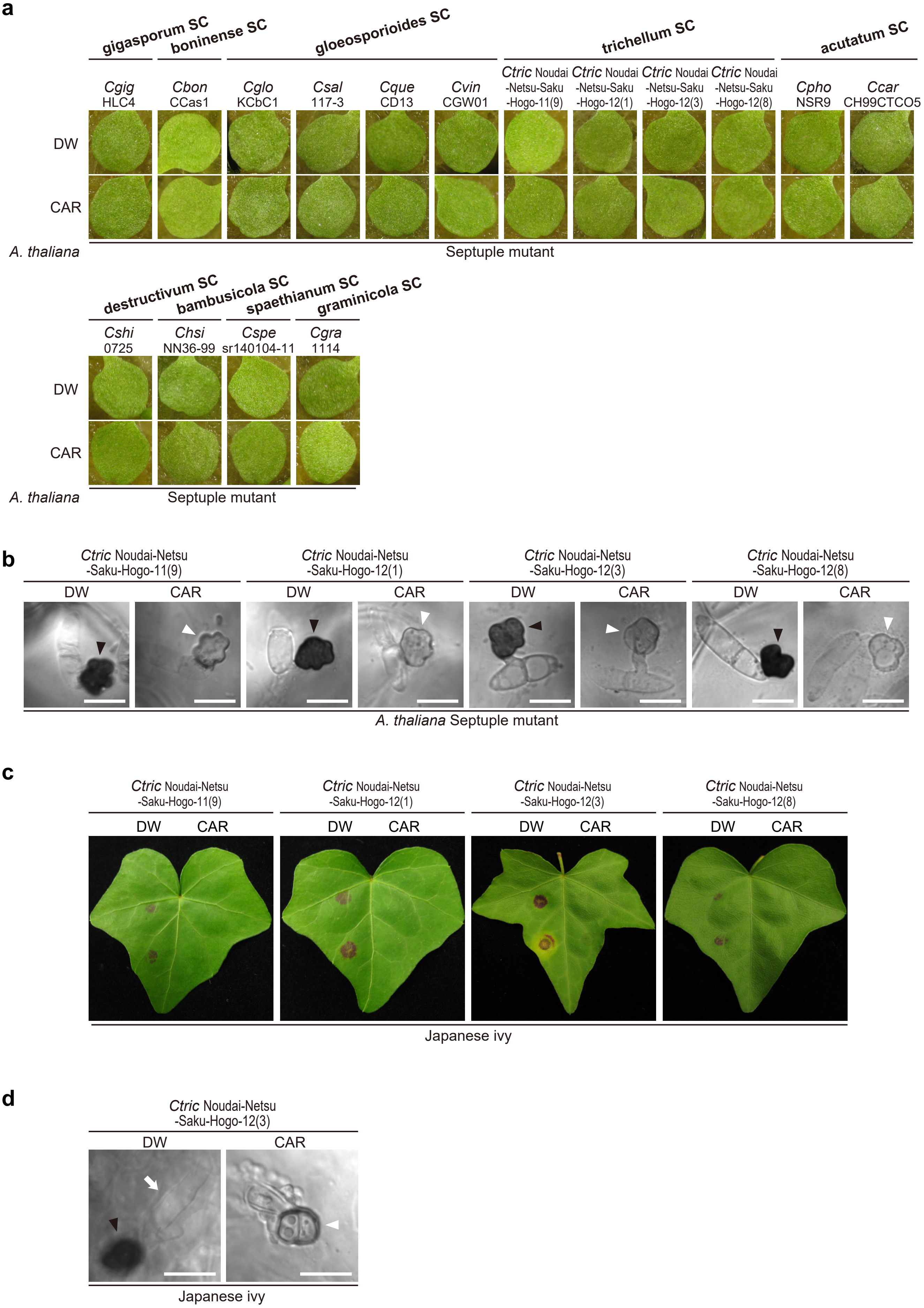
a
gigasporum SC
boninense SC
gloeosporioides SC
trichellum SC
acutatum SC
Cgig HLC4
Cbon CCas1
Cglo KCbC1
Csal 117-3
Cque CD13
Cvin CGW01
Ctric Noudai -Netsu-Saku -Hogo-11(9)
Ctric Noudai -Netsu-Saku -Hogo-12(1)
Ctric Noudai -Netsu-Saku -Hogo-12(3)
Ctric Noudai -Netsu-Saku -Hogo-12(8)
Cpho NSR9
Ccar CH99CTCO5
DW
CAR
A. thaliana
Septuple mutant
destructivum SC
bambusicola SC
spaethianum SC
graminicola SC
Cshi 0725
Chsi NN36-99
Cspe sr140104-11
Cgra 1114
DW
CAR
A. thaliana
Septuple mutant
b
Ctric Noudai-Netsu -Saku-Hogo-11(9)
Ctric Noudai-Netsu -Saku-Hogo-12(1)
Ctric Noudai-Netsu -Saku-Hogo-12(3)
Ctric Noudai-Netsu -Saku-Hogo-12(8)
DW
CAR
A. thaliana Septuple mutant
c
Ctric Noudai-Netsu -Saku-Hogo-11(9)
Ctric Noudai-Netsu -Saku-Hogo-12(1)
Ctric Noudai-Netsu -Saku-Hogo-12(3)
Ctric Noudai-Netsu -Saku-Hogo-12(8)
DW
CAR
Japanese ivy
d
Ctric Noudai-Netsu -Saku-Hogo-12(3)
DW
CAR
Japanese ivy

Supplementary Fig. 9. ***C. trichellum*** **strains are melanised appressorium-mediated entry (MAE)-type pathogens.**

a *Colletotrichum* strains with no lesion formation, even on the *Arabidopsis thaliana* septuple immunocompromised mutants (*edr1 pen2 gsh1 eds5 ein2 cas chup1*) in candidate screening of NMAE-type pathogens. Various nonadapted *Colletotrichum* strains, including *C. gigasporum* (*Cgig*) HLC4, *C.* boninense (*Cbon*) CCas1, *C. gloeosporioides* (*Cglo*) KCbC1, *C. salsolae* (*Csal*) 117-3, *C. queenslandicum* (*Cque*) CD13, *C. viniferum* (*Cvin*) CGW01, *C. trichellum* (*Ctric*) Noudai-Netsu-Saku-Hogo-11(9), *Ctric* Noudai-Netsu-Saku-Hogo-12(1), *Ctric* Noudai-Netsu-Saku-Hogo-12(3), *Ctric* Noudai-Netsu-Saku-Hogo-12(8), *C.phormii* (*Cpho*) NSR9, *C.* carthami (*Ccar*) CH99CTCO5, *C. shisoi* (*Cshi*) 0725, *C. hsienjenchang* (*Chsi*) NN36-99, *C. spaethianum* (*Cspe*) sr140104-11, and *C. graminicola* (*Cgra*) 1114, belonging to various species complexes (SCs) were inoculated onto the cotyledons of *A. thaliana* septuple mutants with or without carpropamid (CAR) and incubated for 7 d. **b** Appressorium formation by *Ctric* strains on *A. thaliana*. A conidial suspension of each *Ctric* strain was inoculated onto the cotyledons of *A. thaliana* septuple mutants, with or without CAR. The inoculated cotyledons were observed at 4 d post-inoculation (dpi). Arrowheads indicate melanised (black) and nonmelanised (white) appressoria. Scale bar = 10 µm. **c** Pathogenicity of the four *Ctric* strains on host plants. A conidial suspension of each strain was inoculated onto adult leaves of Japanese ivy with or without CAR and incubated for 12 d. **d** Fungal invasion of *Ctric* Noudai-Netsu-Saku-Hogo-12(3) on the host epidermis via MAE. A conidial suspension was inoculated onto juvenile leaves of Japanese ivy with or without CAR. The inoculated leaves were observed at 4 dpi. Arrowheads indicate melanised (black) and nonmelanised (white) appressoria. The white arrow indicates invasive hyphae. Scale bar = 10 µm.

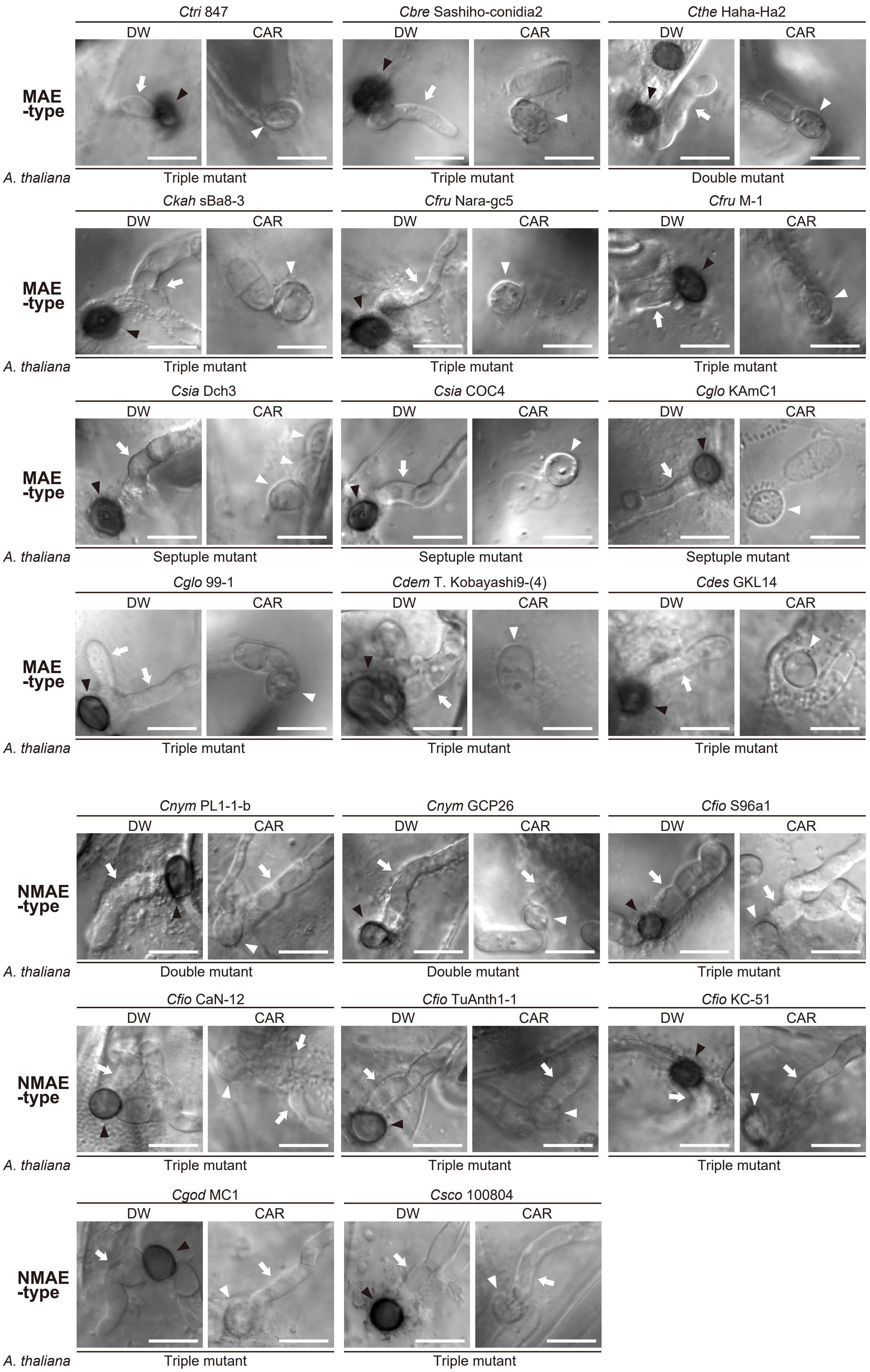

Ctri 847
Cbre Sashiho-conidia2
Cthe Haha-Ha2
DW
CAR
MAE -type
A. thaliana
Triple mutant
Triple mutant
Double mutant
Ckah sBa8-3
Cfru Nara-gc5
Cfru M-1
MAE -type
A. thaliana
Triple mutant
Triple mutant
Triple mutant
Csia Dch3
Csia COC4
Cglo KAmC1
MAE -type
A. thaliana
Septuple mutant
Septuple mutant
Septuple mutant
Cglo 99-1
Cdem T. Kobayashi9-(4)
Cdes GKL14
MAE -type
A. thaliana
Triple mutant
Triple mutant
Triple mutant
Cnym PL1-1-b
Cnym GCP26
Cfio S96a1
NMAE -type
A. thaliana
Double mutant
Double mutant
Triple mutant
Cfio CaN-12
Cfio TuAnth1-1
Cfio KC-51
NMAE -type
A. thaliana
Triple mutant
Triple mutant
Triple mutant
Cgod MC1
Csco 100804
NMAE -type
A. thaliana
Triple mutant
Triple mutant

Supplementary Fig. 10. Many nonmelanised appressorium-mediated entry (NMAE)-type *Colletotrichum* fungi were identified.

Classification of MAE- and NMAE-type *Colletotrichum* fungi. A conidial suspension of various nonadapted *Colletotrichum* strains, including, *C. trifolii* (*Ctrif*) 847, *C. brevisporum* (*Cbre*) Sashiho-conidia2, *C. theobromicola* (*Cthe*) Haha-Ha2, *C. kahawae* (*Ckah*) sBa8-3, *C. fructicola* (*Cfru*) Nara-gc5, *Cfru* M-1, *C. siamense* (*Csia*) Dch3, *Csia* COC4, *C. gloeosporioides* (*Cglo*) KAmC1, *Cglo* 99-1, *C. dematium* (*Cdem*) T. Kobayashi 9-(4), *C. nymphaeae* (*Cnym*) PL1-1-b, *Cnym* GCP26, *C. fioriniae* (*Cfio*) S96a1, *Cfio* CaN-12, *Cfio* TuAnth1-1, *Cfio* KC-51, *C. godetia* (*Cgod*) MC1, *C. scovillei* (*Csco*) 100804, and *C. destructivum* (*Cdes*) GKL14, were inoculated onto the cotyledons of *A. thaliana* double (*edr1 pen2*), triple (*edr1 pen2 gsh1*), or septuple (*edr1 pen2 gsh1 eds5 ein2 cas chup1*) immunocompromised mutants with or without carpropamid (CAR). The inoculated cotyledons were observed at 4 d post-inoculation (dpi). Only the inoculum of *Ctrif* 847 was observed at 7 dpi. Arrowheads indicate melanised (black) and nonmelanised (white) appressoria. White arrows indicate invasive hyphae. Scale bar = 10 µm.

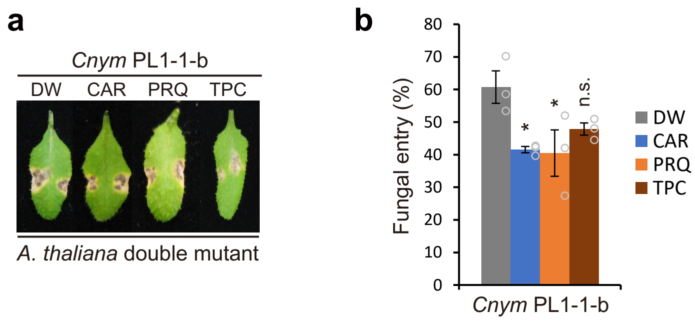

Supplementary Fig. 11. Plant invasion and lesion formation of *C. nymphaeae* PL1-1-b that is insensitive to agrochemical melanin biosynthesis inhibitors (MBIs).

a Efficacy of MBIs against lesion formation in *Arabidopsis* mutants by *Cnym* PL1-1-b. A conidial suspension of *Cnym* PL1-1-b was inoculated onto the leaves of *Arabidopsis thaliana* double (*edr1 pen2*) immunocompromised mutants with or without MBIs, carpropamid (CAR), pyroquilon (PRQ), and tolprocarb (TPC), and incubated for 7 d. **b** Fungal invasion of *Arabidopsis* epidermis. A conidial suspension was inoculated onto the cotyledons of *A. thaliana* double mutants, with or without MBIs. The entry ratio was quantified at 4 d post-inoculation (dpi). At least 100 appressoria were investigated. The mean and SE were calculated from three independent plants. Asterisks indicate significant differences from the control (DW) (*P < 0.05, one-way analysis of variance with Dunnett's test). n.s.: not significant.

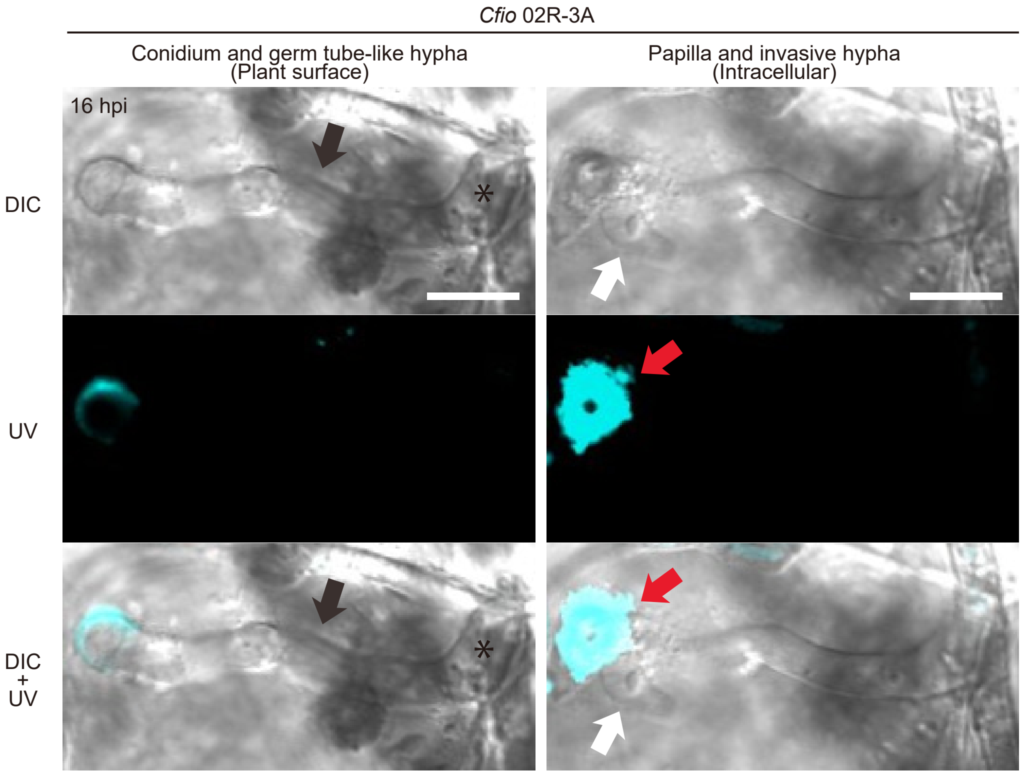

Supplementary Fig. 12. Hyphal tip-based entry (HTE)-type *Colletotrichum* strain initiate invasion through differentiation of penetration peg-like structures.

Papilla formation at the hyphal tip-based entry (HTE) trial site of *Colletotrichum fioriniae* (*Cfio*) 02R-3A. A conidial suspension of nonadapted sugar-induced HTE-type *Cfio* 02R-3A was inoculated onto the cotyledons of *A. thaliana* double (*edr1 pen2*) immunocompromised mutants with 0.5% glucose. Inoculated plants were subjected to staining with aniline blue fluorochrome for visualisation of callose deposition. At 16 h post-inoculation (hpi), callose was visualised under ultraviolet excitation. DIC images were captured using confocal microscopy. Black and white arrows indicate the elongated germ tube-like and invasive hyphae, respectively. The red arrow indicates papillary callose deposition underneath the HTE site. Asterisks indicate conidia. Scale bar = 10 µm.

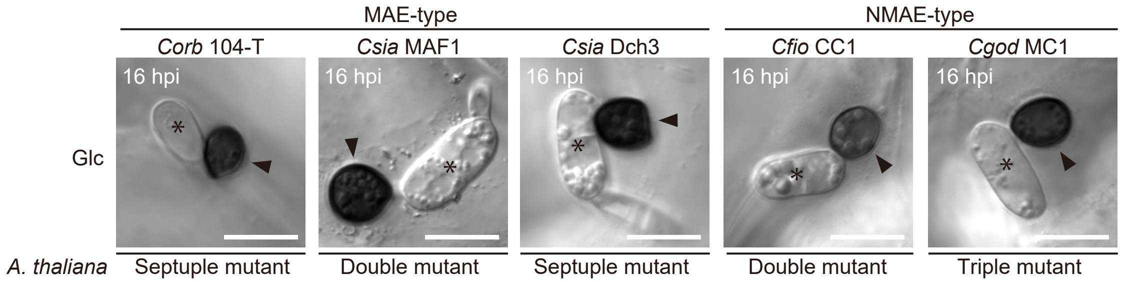

Supplementary Fig. 13. Some appressorium mediated entry-type *Colletotrichum* fungi do not exhibit HTE-related morphogenesis.

No induction of HTE-related morphogenesis in some melanised appressorium-mediated entry (MAE)- and nonmelanised appressorium-mediated entry (NMAE)-type *Colletotrichum* fungi in the presence of sugar was confirmed. Conidial suspensions of *C. orbiculare* (*Corb*) 104-T, *C. siamense* (*Csia*) MAF1, *Csia* Dch3, *C. fioriniae* (*Cfio*) CC1, and *C. godetiae* (*Cgod*) MC1 were inoculated onto the cotyledons of *Arabidopsis thaliana* double (*edr1 pen2*), triple (*edr1 pen2 gsh1*), or septuple (*edr1 pen2 gsh1 eds5 ein2 cas chup1*) mutants with glucose (Glc). Inoculated cotyledons were observed at 16 h post-inoculation (hpi). Arrowheads indicate melanised appressoria. The asterisks indicate conidia. Scale bar = 10 µm.

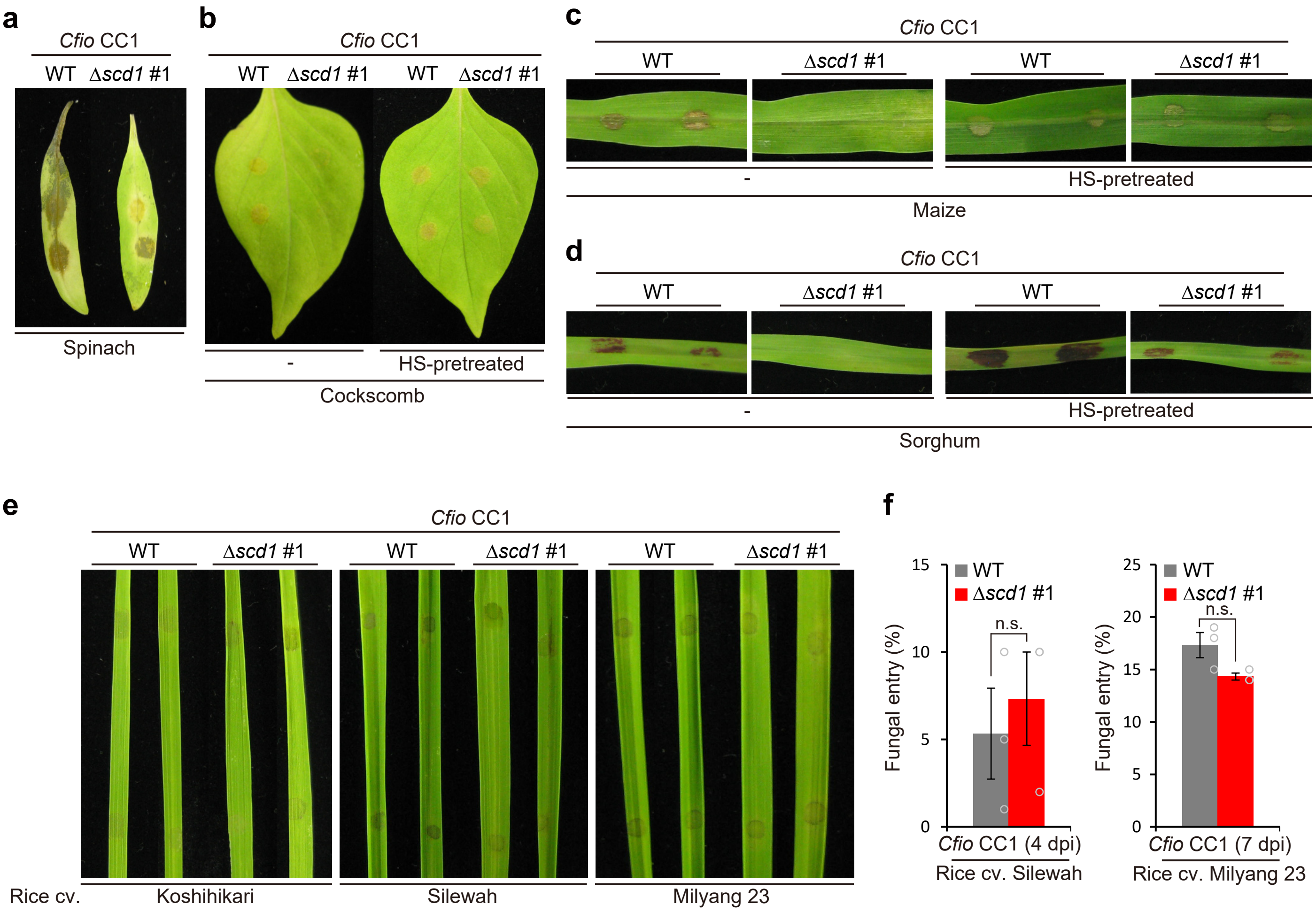

Supplementary Fig. 14. Plant species-dependent lesion induction by nonmelanised appressorium-mediated entry (NMAE) in *C. fioriniae* CC1 on herbaceous plants.

a–e Effects of appressorial melanisation defects on lesion formation in the interactions between *Cfio* CC1 and several herbaceous plant species. Conidial suspensions of *Cfio* CC1 wild-type and Δ*scd1* were inoculated onto the leaves of spinach, cockscomb, maize, sorghum, and rice cultivars ('Koshihikari', 'Silewah', and 'Milyang 23'). The inoculated plants were incubated for 10 (spinach and cockscomb), 7 (maize and sorghum), or 5 d (rice). **b–d** Plant leaves were pretreated with heat shock (HS) to partially compromise plant immunity. **f** Fungal invasion of the rice epidermis. Conidial suspensions of *Cfio* CC1 wild-type and Δ*scd1* were inoculated onto rice leaf sheaths (cvs. Silewah and Milyang 23). The entry ratio was quantified at 4 or 7 d post-inoculation (dpi). A total of 100 appressoria were investigated. The mean and SE were calculated from three independent plants. Differences between means were compared using an unpaired *t*-test (two-tailed). n.s.: not significant.

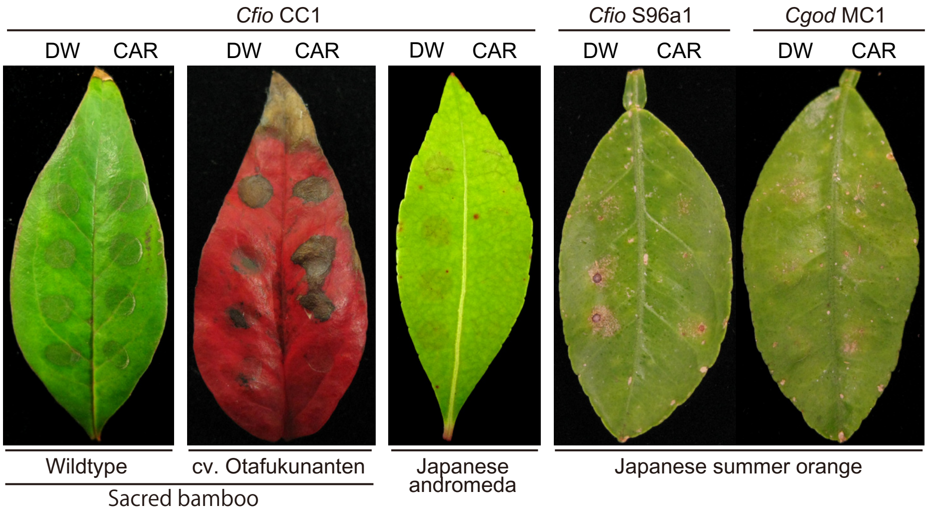

Supplementary Fig. 15. Plant species-dependent lesion induction by nonmelanised appressorium-mediated entry (NMAE)-type *Colletotrichum* fungi on woody plants.

Effects of the melanin biosynthesis inhibitor carpropamid (CAR) on lesion formation in the interactions between NMAE-type *Colletotrichum* fungi and several woody plant species. Conidial suspensions of *Cfio* CC1, *Cfio* S96a1, or *Cgod* MC1 were inoculated onto the leaves of sacred bamboo wild-type and cv. 'Otafukuknanten', Japanese andromeda, or Japanese summer orange. The inoculated plants were incubated for 6 (sacred bamboo and Japanese andromeda) or 7 d (Japanese summer orange).

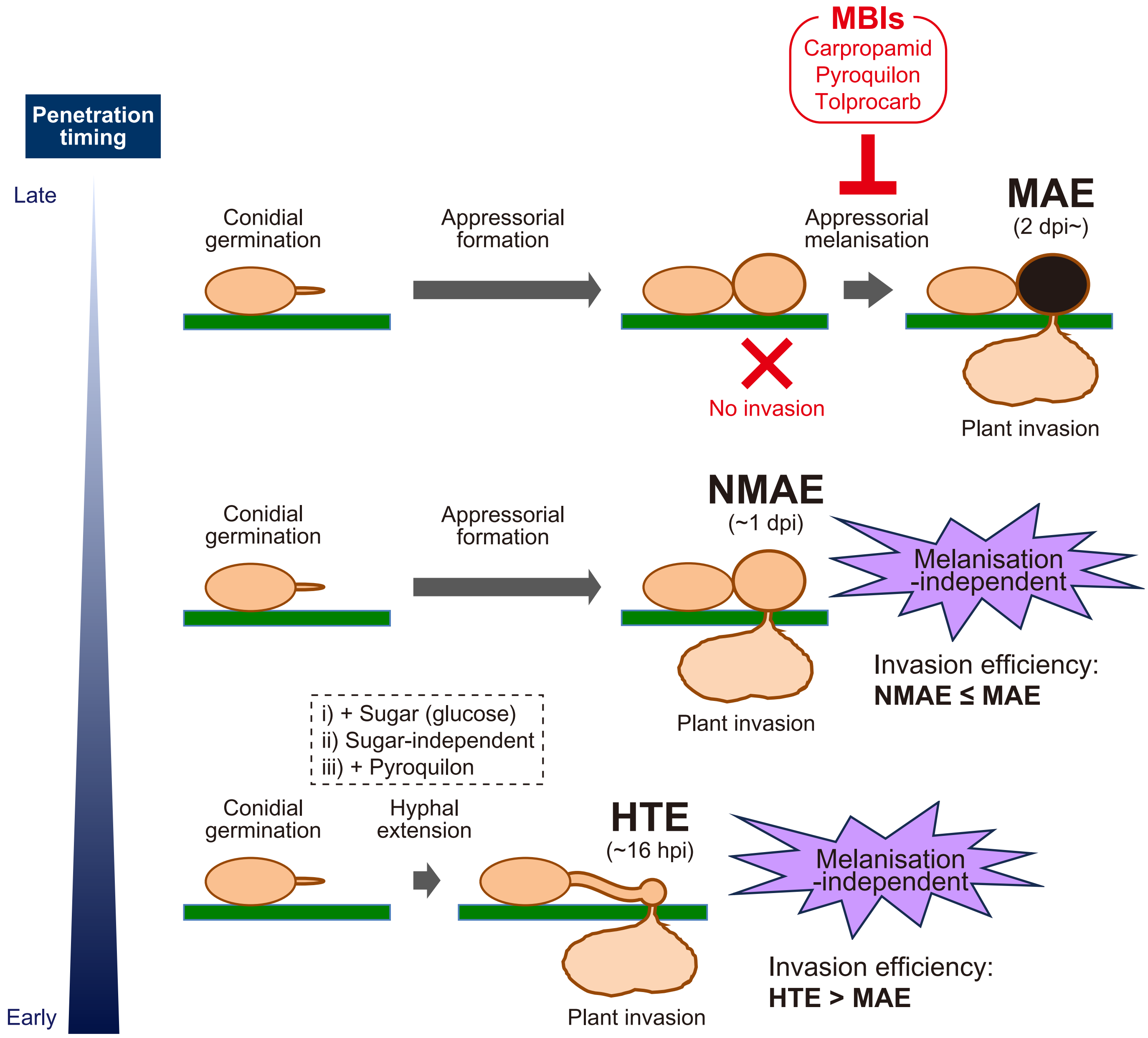

Supplementary Fig. 16. Three types of entry mode into the plant epidermis by *Colletotrichum* fungi.

Upper: Conventional melanised appressorium-mediated entry (MAE). Most *Colletotrichum* fungi, similar to *Pyricularia*, are MAE-type. They exhibit absolute dependence on melanisation and start to invade plants via the appressorium, usually after 2 d post-inoculation (dpi). MAE-type pathogens are sensitive to melanin biosynthesis inhibitors (MBIs), such as carpropamid, pyroquilon, and tolprocarb, and nonmelanised appressoria cannot develop into invasive hyphae. **Middle:** Unprecedented nonmelanised appressorium-mediated entry (NMAE). *Colletotrichum* strains belonging to the acutatum species complex are NMAE-type, exhibit low dependence on melanisation, and begin to invade plants via the appressorium by 1 dpi. NMAE-type pathogens are MBI-insensitive, and nonmelanised appressoria can develop into invasive hyphae. The invasion efficiency of NMAE may be comparable to or lower than that of MAE. **Lower:** Rediscovered hyphal tip-based entry (HTE). Specific *Colletotrichum* strains belonging to the gloeosporioides and acutatum species complexes are HTE-type, which suppress appressorium formation in the presence of sugar and sufficiently invade plants via HTE by 16 h post-inoculation (hpi). Some pathogens exhibit sugar-independent or pyroquilon-induced HTE. HTE-type pathogens are MBI-insensitive, and the invasion efficiency of HTE may be greater than that of MAE.
