# Supplementary material for: Breaking dependence on melanisation imparts diversity to a dogmatic invasion strategy of phytopathogenic fungi
Source: Nat Commun. 2026 Jun 27;17:6126. doi: 10.1038/s41467-026-74937-6 (PMC13365536; doi:10.1038/s41467-026-74937-6)
Supplement: Supplementary file 2 — Descriptions of Additional Supplementary Files [file 41467_2026_74937_MOESM2_ESM.pdf]

Descriptions of Additional Supplementary Files

Supplementary Data 1. Fungal strains used in this study.

Supplementary Data 2. Primers used for the genotyping of Arabidopsis.

Supplementary Data 3. Primers used for the plasmid construction and fungal gene disruption
