# Supplementary material for: Breaking dependence on melanisation imparts diversity to a dogmatic invasion strategy of phytopathogenic fungi
Source: Nat Commun. 2026 Jun 27;17:6126. doi: 10.1038/s41467-026-74937-6 (PMC13365536; doi:10.1038/s41467-026-74937-6)
Supplement: Supplementary file 3 — Supplementary Data 1 [file 41467_2026_74937_MOESM3_ESM.docx]

**Supplementary Data 1. Fungal strains used in this study.**

| **Strain** | **MAFF No.** | **Source** |
| --- | --- | --- |
| *Colletotrichum orbiculare* (syn. *C. lagenarium*) 104-T | 240422 | Laboratory of Plant Pathology, Kyoto University (Dr. Yoshitaka Takano) |
| *Colletotrichum trifolii* 847 | 305383 | Ministry of Agriculture, Forestry and Fisheries GenBank, Japan |
| *Colletotrichum brevisporum* Sashiho-conidia2 | 305751 | Ministry of Agriculture, Forestry and Fisheries GenBank, Japan |
| *Colletotrichum gigasporum* HLC4 | 243177 | Ministry of Agriculture, Forestry and Fisheries GenBank, Japan |
| *Colletotrichum boninense* CCas1 | 305972 | Ministry of Agriculture, Forestry and Fisheries GenBank, Japan |
| *Colletotrichum theobromicola* Haha-Ha2 | 305994 | Ministry of Agriculture, Forestry and Fisheries GenBank, Japan |
| *Colletotrichum kahawae* sBa8-3 | 240193 | Ministry of Agriculture, Forestry and Fisheries GenBank, Japan |
| *Colletotrichum fructicola* (syn. *C. gloeosporioides*) Nara-gc5 | 245243 | Ministry of Agriculture, Forestry and Fisheries GenBank, Japan |
| *Colletotrichum fructicola* M-1 | 731010 | Ministry of Agriculture, Forestry and Fisheries GenBank, Japan |
| *Colletotrichum siamense* MAF1 | 243010 | Ministry of Agriculture, Forestry and Fisheries GenBank, Japan |
| *Colletotrichum siamense* Dch3 | 240428 | Ministry of Agriculture, Forestry and Fisheries GenBank, Japan |
| *Colletotrichum siamense* COC4 | 243696 | Ministry of Agriculture, Forestry and Fisheries GenBank, Japan |
| *Colletotrichum gloeosporioides* KAmC1 | 243180 | Ministry of Agriculture, Forestry and Fisheries GenBank, Japan |
| *Colletotrichum gloeosporioides* 99-1 | 240510 | Ministry of Agriculture, Forestry and Fisheries GenBank, Japan |
| *Colletotrichum gloeosporioides* KCbC1 | 243182 | Ministry of Agriculture, Forestry and Fisheries GenBank, Japan |
| *Colletotrichum salsolae* 117-3 | 240542 | Ministry of Agriculture, Forestry and Fisheries GenBank, Japan |
| *Colletotrichum queenslandicum* CD13 | 244212 | Ministry of Agriculture, Forestry and Fisheries GenBank, Japan |
| *Colletotrichum viniferum* CGW01 | 665013 | Ministry of Agriculture, Forestry and Fisheries GenBank, Japan |
| *Colletotrichum tropicale* S9275 (syn. *C. gloeosporioides* S9275) +mRFP | 840071 (Original strain) | Laboratory of Plant Pathology, Kyoto University (Dr. Yoshitaka Takano) |
| *Colletotrichum tropicale* Ishigaki-Banana2 | 306174 | Ministry of Agriculture, Forestry and Fisheries GenBank, Japan |
| *Colletotrichum musae* Noudai-Netsu-Saku-Hogo-28(3) | 239087 | Ministry of Agriculture, Forestry and Fisheries GenBank, Japan |
| *Colletotrichum dematium* T. Kobayashi 9-(4) | 236699 | Ministry of Agriculture, Forestry and Fisheries GenBank, Japan |
| *Colletotrichum trichellum* Noudai-Netsu-Saku-Hogo-11(9) | 237992 | Ministry of Agriculture, Forestry and Fisheries GenBank, Japan |
| *Colletotrichum trichellum* Noudai-Netsu-Saku-Hogo-12(1) | 238020 | Ministry of Agriculture, Forestry and Fisheries GenBank, Japan |
| *Colletotrichum trichellum* Noudai-Netsu-Saku-Hogo-12(3) | 238022 | Ministry of Agriculture, Forestry and Fisheries GenBank, Japan |
| *Colletotrichum trichellum* Noudai-Netsu-Saku-Hogo-12(8) | 238027 | Ministry of Agriculture, Forestry and Fisheries GenBank, Japan |
| *Colletotrichum nymphaeae* PL1-1-b | 240037 | Ministry of Agriculture, Forestry and Fisheries GenBank, Japan |
| *Colletotrichum nymphaeae* GCP26 | 306505 | Ministry of Agriculture, Forestry and Fisheries GenBank, Japan |
| *Colletotrichum fioriniae* CC1 | 306550 | Ministry of Agriculture, Forestry and Fisheries GenBank, Japan |
| *Colletotrichum fioriniae* S96a1 | 238647 | Ministry of Agriculture, Forestry and Fisheries GenBank, Japan |
| *Colletotrichum fioriniae* HF3 | 306542 | Ministry of Agriculture, Forestry and Fisheries GenBank, Japan |
| *Colletotrichum fioriniae* CaN-12 | 244084 | Ministry of Agriculture, Forestry and Fisheries GenBank, Japan |
| *Colletotrichum fioriniae* TuAnth1-1 | 243478 | Ministry of Agriculture, Forestry and Fisheries GenBank, Japan |
| *Colletotrichum fioriniae* KC-51 | 245595 | Ministry of Agriculture, Forestry and Fisheries GenBank, Japan |
| *Colletotrichum fioriniae* 02R-3A | 241878 | Ministry of Agriculture, Forestry and Fisheries GenBank, Japan |
| *Colletotrichum fioriniae* NIAS G 4-1-4 | 305140 | Ministry of Agriculture, Forestry and Fisheries GenBank, Japan |
| *Colletotrichum fioriniae* Kishi-5(4) | 237240 | Ministry of Agriculture, Forestry and Fisheries GenBank, Japan |
| *Colletotrichum aenigma* IS4 | 306553 | Ministry of Agriculture, Forestry and Fisheries GenBank, Japan |
| *Colletotrichum godetiae* MC1 | 241296 | Ministry of Agriculture, Forestry and Fisheries GenBank, Japan |
| *Colletotrichum godetiae* FA06-48 | 240289 | Ministry of Agriculture, Forestry and Fisheries GenBank, Japan |
| *Colletotrichum scovillei* 100804 nagano shimoina | 243038 | Ministry of Agriculture, Forestry and Fisheries GenBank, Japan |
| *Colletotrichum phormii* NSR9 | 245099 | Ministry of Agriculture, Forestry and Fisheries GenBank, Japan |
| *Colletotrichum carthami* CH99CTCO5 | 239372 | Ministry of Agriculture, Forestry and Fisheries GenBank, Japan |
| *Colletotrichum higginsianum* Abr1-5 | 305635 | Ministry of Agriculture, Forestry and Fisheries GenBank, Japan |
| *Colletotrichum destructivum* GKL14 | 235094 | Ministry of Agriculture, Forestry and Fisheries GenBank, Japan |
| *Colletotrichum shisoi* 0725 | 240106 | Ministry of Agriculture, Forestry and Fisheries GenBank, Japan |
| *Colletotrichum hsienjenchang* NN36-99 | 243051 | Ministry of Agriculture, Forestry and Fisheries GenBank, Japan |
| *Colletotrichum spaethianum* sr140104-11 | 245208 | Ministry of Agriculture, Forestry and Fisheries GenBank, Japan |
| *Colletotrichum graminicola* 1114 | 305427 | Ministry of Agriculture, Forestry and Fisheries GenBank, Japan |
| *Pyricularia oryzae* Ina86-137 | 101511 | Ministry of Agriculture, Forestry and Fisheries GenBank, Japan |
