# Supplementary material for: Breaking dependence on melanisation imparts diversity to a dogmatic invasion strategy of phytopathogenic fungi
Source: Nat Commun. 2026 Jun 27;17:6126. doi: 10.1038/s41467-026-74937-6 (PMC13365536; doi:10.1038/s41467-026-74937-6)
Supplement: Supplementary file 4 — Supplementary Data 2 [file 41467_2026_74937_MOESM4_ESM.docx]

**Supplementary Data 2. Primers used for the genotyping of *Arabidopsis*.**

| **Name** | **Sequence (5′-3′)** | **Mutation and restriction enzyme** |
| --- | --- | --- |
| pen2-1F | TCAGGTAAATCAGTTCGAATCAAGAAC | *pen2-1*  *Bam*HI |
| pen2-1R | TGAGGAAACCTGTTGGAGAAAGGATC |  |
| ein2-1F | GGATACTACGTCTGTTACTAGC | *ein2-1*  *Xho*I |
| ein2-1R | GTCTTCCTTAAGACTACTAACTC |  |
| edr1-1F | CAGAGGCTGAAAGGACAGATTCTTGGTA | *edr1-1*  *Kpn*I |
| edr1-1R | CCTCACTGTTCTGATTGTAAGG |  |
| gsh1-1F | TTACAACCTGTGAGAGCTCG | *gsh1-1*  *Sph*I |
| gsh1-1R | CTGAATCTAGATACCTTCGCATG |  |
| eds5-1F | CTTGGTCTAATCTGATTCTTGATATGTTTTCTA | *eds5-1*  *Xba*I |
| eds5-1R | GAGACTTATTCAGCTGCTTGCTTCTC |  |
| atcas-LP | CGCTTCTTTGATTACCAATCG | *cas* |
| atcas-RP | TCAAACCCTAAACCCCAAAAC |  |
| atchup1-LP2 | CATCTAGCCAGCTAACGAACG | *chup1* |
| atchup1-RP2 | CTTAAGAAATGGGGCAAAAGC |  |
| LBb1.3 | ATTTTGCCGATTTCGGAAC | *cas*, *chup1* |
