# Supplementary material for: Breaking dependence on melanisation imparts diversity to a dogmatic invasion strategy of phytopathogenic fungi
Source: Nat Commun. 2026 Jun 27;17:6126. doi: 10.1038/s41467-026-74937-6 (PMC13365536; doi:10.1038/s41467-026-74937-6)
Supplement: Supplementary file 5 — Supplementary Data 3 [file 41467_2026_74937_MOESM5_ESM.docx]

**Supplementary Data 3. Primers used for the plasmid construction and fungal gene disruption.**

| **Name** | **Sequence (5’-3’)** | **Destination** |
| --- | --- | --- |
| ChigSCD1-5XhoI-f | CCGCTCGAGTGGGTGATTCGAGGGGCTATG | *SCD1* gene disruption vector for *Chig* Abr1-5 (pHYChigAbr1-5SCD1del) |
| ChigSCD1-5XhoI-r | CCGCTCGAGTTTGAGTGATGGTGGGAGAAGAGG |  |
| ChigSCD1-3EcoRV-f | CCGATATCAGTACAATATGAAGAAGGC |  |
| ChigSCD1-3XbaI-r | GCTCTAGAAATGAGACGTCAAGCCACAGATCC |  |
| CorbSCD1-5ApaI-f | GGGGGCCCATCATGGAGACGGACTATGCCAC | *SCD1* gene disruption vector for *Corb* 104-T (pHYCorb104-TSCD1del) |
| CorbSCD1-5XhoI-r | CCGCTCGAGGATGTCTGATAGGTGGGATATTACGTGG |  |
| CorbSCD1-3EcoRV-f | CCGATATCAGCGGCGTGCTCTGCACATAAC |  |
| CorbSCD1-3SpeI-r | GACTAGTAGCTCGCACTTGACGTATATCG |  |
| CsiaSCD1-5KpnI-f | GGGGTACCGTTAACACATCGGCTCTTCGCC | *SCD1* gene disruption vector for *Csia* MAF1 (pHYCsiaMAF1SCD1del) |
| CsiaSCD1-5KpnI-r | GGGGTACCTATGTCTACTTTTCGAAATTACTCG |  |
| CsiaSCD1-3HindIII-f | CCCAAGCTTATATGTCATATGGGCGTGGAATGG |  |
| CsiaSCD1-3SpeI-r | GACTAGTGGATCGCGCTATTGTGTTGC |  |
| CfioSCD1-5XhoI-f | CCGCTCGAGACCAAAGACTCTCTTGG | *SCD1* gene disruption vector for *Cfio* CC1 (pHYCfioCC1SCD1del) |
| CfioSCD1-5XhoI-r | CCGCTCGAGGTTTGATCACGTCTGACGTG |  |
| CfioSCD1-3ClaI-f | CCATCGATATAATGTCAATGTAATGAGGGAATACCTCCGG |  |
| CfioSCD1-3SpeI-r | GACTAGTCGGCCAACAACAAGATACTTGC |  |
| ChigSCD1OUTS | CCCCCGTCGAATTATAAACCC | Genomic PCR of Δ*scd1* mutants for *Chig* Abr1-5 |
| ChigSCD1OUTAS | CTCACAAGTTACACACGAGGTG |  |
| CorbSCD1OUTS | GAGACCGGACTGGCTTCCTTC | Genomic PCR of Δ*scd1* mutants for *Corb* 104-T |
| CorbSCD1OUTAS | CGTGTGTCGGGGTAAGTTGTAG |  |
| CsiaSCD1OUTS | CCCTTTCGCTTTGCGGGATGAG | Genomic PCR of Δ*scd1* mutants for *Csia* MAF1 |
| CsiaSCD1OUTAS | CGGTATTGCAAGATTTGTGAAGG |  |
| CfioSCD1OUTS | GATTGTGTTCCAAGTTCAACGC | Genomic PCR of Δ*scd1* mutants for *Cfio* CC1 |
| CfioSCD1OUTAS | CTTTGTGCCGACGTCCTCAG |  |
