# Supplementary material for: Breaking dependence on melanisation imparts diversity to a dogmatic invasion strategy of phytopathogenic fungi
Source: Nat Commun. 2026 Jun 27;17:6126. doi: 10.1038/s41467-026-74937-6 (PMC13365536; doi:10.1038/s41467-026-74937-6)
Supplement: Supplementary file 7 — Transparent Peer Review file [file 41467_2026_74937_MOESM7_ESM.pdf]

nature portfolio

Peer Review File

Breaking dependence on melanisation imparts diversity to a dogmatic invasion strategy of phytopathogenic fungi

Corresponding Author: Dr Hiroki Irieda

This file contains all reviewer reports in order by version, followed by all author rebuttals in order by version.

Version 0:

Reviewer comments:

Reviewer #1

(Remarks to the Author)
Manuscript Title
Emergence of melanin biosynthesis inhibitor-resistant and rapidly invasive phytopathogenic fungi

Summary
In this manuscript, the authors report the identification and characterization of a group of *Colletotrichum* fungi that can invade host plants without melanized appressoria, via nonpenalized appressorium-mediated entry (NMAE), as well as additional hyphal tip-based entry (HTE)–type strains. Through extensive phenotypic, phylogenetic, and infection assays, the study challenges the long-standing paradigm that appressorial melanization is universally essential for plant invasion in *Colletotrichum* and related phytopathogenic fungi.
The discovery of melanization-independent infection strategies, particularly within the *Colletotrichum acutatum* species complex, is novel and potentially impactful. However, several conceptual, terminological, and interpretational issues, especially regarding the use of the term "melanin biosynthesis inhibitor-resistant" and the framing of ecological and evolutionary implications require careful reconsideration before publication.

Major Comments
1. Use of the term "melanin biosynthesis inhibitor-resistant" in the title and throughout the manuscript
The term "melanin biosynthesis inhibitor-resistant" (MBI-resistant) requires clearer definition and justification.
• It is ambiguous whether "resistant" refers to:
・resistance at the enzymatic level (i.e., failure of the inhibitor to block melanin biosynthetic enzymes), or
・resistance at the phenotypic/pathological level (i.e., maintenance of infection or virulence despite inhibition of melanization).
• In agricultural field, melanin biosynthesis inhibitors are used exclusively for rice blast disease caused by *Pyricularia oryzae*. They are not applied for diseases caused by *Colletotrichum*. Therefore, applying the term "MBI-resistant" to *Colletotrichum* strains risks deviating from the conventional and regulatory definition of fungicide resistance in agroecosystems.
• Importantly, the data presented for *C. acutatum*–group strains indicate that melanin biosynthesis is still inhibited, yet pathogenicity is retained. This phenotype is better described as MBI-insensitive infection or melanisation-independent infection, rather than resistance.
• The title phrase "Emergence of melanin biosynthesis inhibitor-resistant…" is potentially misleading:
・"Emergence" implies the recent appearance of a novel resistant population, which is not directly demonstrated.
・The traits described may instead reflect intrinsic properties of the *C. acutatum* lineage, rather than newly evolved resistance.
Given these points, the current title may mislead readers, particularly those in plant pathology, agrochemical science, or resistance management. The core message of the study is the discovery of melanin-independent infection strategies, which represents a paradigm shift relative to established dogma. A title reflecting this conceptual advance, rather than fungicide resistance, would be more accurate.

2. Conceptual alignment between the title, Introduction, and main conclusions
The final sentence of the Introduction states:
"In the present study, we identified and characterized a novel group of *Colletotrichum* fungi that can invade plants via

nonmelanised appressorium-mediated entry (NMAE) and rediscovered HTE-type strains."
This sentence accurately captures the primary contribution of the work. However, it does not align well with the emphasis on "MBI-resistant" pathogens suggested by the title.
Similarly, the statement:
"Our findings challenge the prevailing notion that appressorial formation and melanization are universally essential for plant infection."
appears overly broad. The findings are highly significant for *Colletotrichum* and possibly *Pyricularia,* but do not justify a generalization to all plant pathogenic fungi. The authors should narrow this claim to the taxa actually examined.

3. Experimental framing and ecological relevance of MBI-related arguments
Several sections of the manuscript discuss the potential emergence and risk of MBI-resistant pathogens in agricultural ecosystems. However:
• MBI compounds are not applied to crops affected by *Colletotrichum*.
• The experimental systems used do not reflect the systemic application, field-level exposure, or selective pressures associated with real-world fungicide use.
• If the authors wish to argue ecological or agronomic risk, experimental designs explicitly addressing these contexts would be required.
Otherwise, the discussion should be reframed to avoid overstating real-world resistance risks.

4. Interpretation of "in nature" and field relevance
The phrase "NMAE-based plant infection in nature" (Line 253) is problematic. The data are derived from controlled laboratory inoculation assays, not field studies. This wording could be misinterpreted as evidence for natural infection dynamics. A more precise phrasing (e.g., "across multiple species" or "in diverse *Colletotrichum* lineages") would be preferable.

5. Evolutionary and mechanistic implications within the *C. acutatum* group
The *C. acutatum* species complex is known for:
• broad host range,
• high aggressiveness,
• relative tolerance to chemical control.
The data presented (e.g., Fig. 4a, Supplementary Fig. 13) suggest that NMAE-type strains may possess fundamentally different infection programs, potentially relying more heavily on enzymatic cell wall degradation or secondary metabolites. Notably, within this clade, species such as *C. orchidophilum* and C. fioriniae lack the tandem Perox–AlcOx metalloenzyme system, which chemically remodels host cuticular components into signaling molecules required for proper appressorium development (Bissaro et al., Sci. Adv., 2022).
Given known differences from *C. orbiculare*, including loss of conserved tandem gene clusters involved in plant surface signal perception, the authors are encouraged to discuss whether appressorium development, polarity establishment, and cell-cycle coupling may be qualitatively distinct in this group. This perspective would significantly strengthen the mechanistic and evolutionary interpretation of the findings.
Please also see section 7.

6. Consideration of Agrochemical Usage and Societal Implications
Line 470: The discussion suggesting that melanin biosynthesis inhibitors may induce or promote hyphal tip–based entry (HTE)–type infection warrants careful reconsideration in light of real-world agrochemical usage and regulatory context. Pyroquilone and related MBI-D fungicides are registered and applied exclusively for the control of rice blast caused by Pyricularia oryzae. To date, there is no experimental or field-based evidence demonstrating that MBI application induces HTE-type invasion in *Pyricularia*, nor that such compounds alter infection strategies under agronomically relevant conditions.
In contrast, HTE-type infection described in this study has been demonstrated only in *Colletotrichum* species, for which melanin biosynthesis inhibitors are not used in agricultural practice. As currently phrased, the discussion may be interpreted as implying a fungicide-driven emergence of alternative infection strategies in crop systems, a claim that carries potential societal and regulatory implications. In the absence of direct supporting evidence, the authors are encouraged to substantially tone down this argument, clearly delineate laboratory observations from field-relevant scenarios, or explicitly frame this idea as speculative rather than established risk.

7. Suggested Perspective: Evolutionary Remodeling of Infection Programs in the *Colletotrichum acutatum* Clade
Findings presented in this study strongly suggest that infection strategies within the Colletotrichum acutatum clade cannot be fully interpreted as simple variants of the canonical melanized appressorium-mediated entry paradigm established for C. orbiculare. Given the reported divergence of this clade, including the loss of conserved gene clusters implicated in plant surface signal perception and the absence of the tandem Perox–AlcOx metalloenzyme system that chemically remodels host cuticular components (Bissaro et al., Sci. Adv., 2022). The data presented in this manuscript are more consistent with a qualitative rewiring of infection-related developmental programs rather than with partial relaxation of melanization-dependent mechanisms.
In C. orbiculare, appressorium morphogenesis is tightly integrated with chemical surface sensing, polarity establishment, and cell-cycle control through conserved pathways such as the MOR/NDR kinase cascade (Kodama et al., PLoS Pathog., 2017) and the Bub2–Bfa1–Tem1 module regulating G1/S progression during infection-related morphogenesis (Fukada & Kubo, Plant Cell, 2015). By contrast, the diversity of strategies observed within the acutatum lineage, including nonmelanized appressorium-mediated entry (NMAE), hyphal tip–based entry (HTE), and the presence or absence of chemical gating mechanisms such as Perox–AlcOx, suggests that these fungi may deploy alternative combinations of morphogenetic timing, polarity control, and host-surface engagement to achieve rapid and effective penetration.
I therefore suggest that the authors explicitly interpret NMAE not merely as exceptions to a melanization-centered dogma, but as outcomes of evolutionary diversification of infection programs within Colletotrichum.

Minor Comments

• Line 124 and elsewhere: The term hyaline may be misleading; hyaline means non-visible, colorless or non-melanized would be more precise.
• Line 140: The term albino is typically annotated for PKS-deficient DHN-melanin mutants. Consider alternative wording.
• Line 149: "MBI-resistant" may be better replaced with "MBI-insensitive" or rephrased.
• Line 318: Fig. 5i is cited but appears to be missing.
• Line 360: Clarify whether the discussion refers to Colletotrichum or Pyricularia. If the latter, direct evidence of NMAE in Pyricularia would be required.

Overall Assessment
This manuscript reports a genuinely important and conceptually novel finding: that melanization is not universally required for appressorium-mediated plant invasion in Colletotrichum. The work challenges a long-standing dogma and is supported by extensive experimental evidence.
However, substantial revision is needed to:
• correct potentially misleading terminology,
• align the title and conclusions with the actual data,
• moderate claims regarding ecological emergence and fungicide resistance,
• and deepen the mechanistic discussion within an evolutionary framework.
With these revisions, the manuscript would represent a strong and influential contribution to the field.

Strengths of the Manuscript
• Discovery of melanin-independent appressorium-mediated infection in the C. acutatum group.
• Rigorous experimental analysis using Arabidopsis immune-deficient mutants.
• Willingness to challenge an entrenched paradigm in fungal plant pathology.
• Insight into qualitative differences in host immune responses depending on infection mode.

Reviewer #2

(Remarks to the Author)
The manuscript describes the existence of a melanin-independent infection mode in Colletotrichum fungi—non-melanized appressorium-mediated entry (NMAE). Strains with this mode exhibit resistance to melanin biosynthesis inhibitors (MBIs) and rapidly penetrate plant tissues via mature non-melanized appressorium. Additionally, strains relying on hyphal tip entry (HTE) were identified, indicating that appressorial melanization is not universally essential. These findings challenge the conventional view that melanization of appressorium is a prerequisite for plant infection and underscore the necessity of considering the emergence of NMAE-type and HTE-type pathogens. However, a major revision is still needed before considering the acceptance.
1. Colletotrichum fungi have a broad host range. Given that the plants inoculated in the manuscript were mostly herbaceous, should woody plants be included as controls to verify the generalizability of the experimental results?
2. Does Pyricularia fungi also exhibit a melanin-independent infection mode?
3. How does the non-melanized appressorium-mediated entry (NMAE) mode maintain turgor in Colletotrichum appressorium?

Reviewer #3

(Remarks to the Author)
This manuscript challenges the long-standing dogma in plant pathology that melanized appressoria are universally essential for host penetration by identifying and characterizing two alternative invasion strategies in Colletotrichum fungi: nonmelanized appressorium-mediated entry (NMAE) and hyphal tip-based entry (HTE). The authors found that NMAE-type strains, which form a rapidly expanding monophyletic group, can invade hosts earlier and independently of melanization. The work employs a combination of phenotypic, phylogenetic, and functional assays, supporting its conclusion that fungal invasion strategies are more diverse than previously recognized. However, this study primarily focuses on the discovery and description of NMAE and HTE phenomenon, with limited exploration of their underlying mechanisms.
1. The title/abstract focuses on Colletotrichum, yet the introduction pairs it with Pyricularia. Are the findings specific to Colletotrichum, or is the paradigm shift suggested to be broader? The manuscript also lacks findings related to NMAE or HTE in Pyricularia.
2. A key mechanistic question is how NMAE appressoria generate sufficient turgor pressure without melanin for penetration. The study notes variation in dependence but does not elucidate the alternative mechanism(s) compensating for the lack of melanin.
3. For the HTE strains, the precise infection process is undescribed. How do hyphal tips overcome plant barriers without a specialized appressorium? Ultrastructural or cytological evidence is lacking.
4. While the study excellently characterizes NMAE in vitro or under controlled conditions (appressoria are formed on the immunocompromised A. thaliana), the agronomic significance and real-world impact remain unclear. Do NMAE and HTE strains cause disease of comparable severity and spread in field conditions? This is critical for assessing the practical implication for disease management.
5. The gene knockout mutants in Colletotrichum used in this study lack southern blotting validation and complementation assays.

Version 1:

Reviewer comments:

Reviewer #1

(Remarks to the Author)
The authors have carefully and thoroughly addressed all of the reviewer’s comments. The revisions have significantly improved the clarity, rigor, and overall quality of the manuscript. In particular, the conceptual framing, terminology, and interpretation have been appropriately refined in line with the reviewer’s suggestions.
I am satisfied with the authors’ responses and consider that the revised manuscript is now suitable for publication in its current form.

Reviewer #2

(Remarks to the Author)
The authors have responded adequately to each of the concerns/suggestions and made changes to reflect their responses in the manuscript itself.The manuscript is know acceptable for publication.

Reviewer #3

(Remarks to the Author)
The authors have addressed the reviewers' comments and revised the manuscript accordingly. I have no additional concerns.

Open Access *This Peer Review File is licensed under a Creative Commons Attribution 4.0 International License, which permits use, sharing, adaptation, distribution and reproduction in any medium or format, as long as you give appropriate credit to the original author(s) and the source, provide a link to the Creative Commons license, and indicate if changes were made.*
In cases where reviewers are anonymous, credit should be given to 'Anonymous Referee' and the source.
The images or other third party material in this Peer Review File are included in the article’s Creative Commons license, unless indicated otherwise in a credit line to the material. If material is not included in the article’s Creative Commons license and your intended use is not permitted by statutory regulation or exceeds the permitted use, you will need to obtain permission directly from the copyright holder.
To view a copy of this license, visit https://creativecommons.org/licenses/by/4.0/

Responses to reviewer 1:

//Reviewer 1 comment:

Reviewer #1 (Remarks to the Author):

Manuscript Title

Emergence of melanin biosynthesis inhibitor-resistant and rapidly invasive phytopathogenic fungi

Summary

In this manuscript, the authors report the identification and characterization of a group of Colletotrichum fungi that can invade host plants without melanized appressoria, via nonpenalized appressorium-mediated entry (NMAE), as well as additional hyphal tip-based entry (HTE)–type strains. Through extensive phenotypic, phylogenetic, and infection assays, the study challenges the long-standing paradigm that appressorial melanization is universally essential for plant invasion in Colletotrichum and related phytopathogenic fungi.

The discovery of melanization-independent infection strategies, particularly within the Colletotrichum acutatum species complex, is novel and potentially impactful. However, several conceptual, terminological, and interpretational issues, especially regarding the use of the term "melanin biosynthesis inhibitor-resistant" and the framing of ecological and evolutionary implications require careful reconsideration before publication.

//Author reply:

Thank you for your critical reading of our manuscript and your insightful suggestions and positive comments, which have helped us to improve our manuscript considerably. Based on the comments provided by the reviewers, we revised our manuscript after performing new experiments. We have addressed all of the reviewers' concerns. Please see our point-by-point responses.

//Reviewer 1 comment:

Major Comments

1. Use of the term "melanin biosynthesis inhibitor-resistant" in the title and throughout the manuscript

The term "melanin biosynthesis inhibitor-resistant" (MBI-resistant) requires clearer definition and justification.

• It is ambiguous whether "resistant" refers to:

•resistance at the enzymatic level (i.e., failure of the inhibitor to block melanin biosynthetic

enzymes), or

•resistance at the phenotypic/pathological level (i.e., maintenance of infection or virulence despite inhibition of melanization).

• In agricultural field, melanin biosynthesis inhibitors are used exclusively for rice blast disease caused by Pyricularia oryzae. They are not applied for diseases caused by Colletotrichum. Therefore, applying the term "MBI-resistant" to Colletotrichum strains risks deviating from the conventional and regulatory definition of fungicide resistance in agroecosystems.

• Importantly, the data presented for C. acutatum–group strains indicate that melanin biosynthesis is still inhibited, yet pathogenicity is retained. This phenotype is better described as MBI-insensitive infection or melanisation-independent infection, rather than resistance.

• The title phrase "Emergence of melanin biosynthesis inhibitor-resistant…" is potentially misleading:

•"Emergence" implies the recent appearance of a novel resistant population, which is not directly demonstrated.

•The traits described may instead reflect intrinsic properties of the C. acutatum lineage, rather than newly evolved resistance.

Given these points, the current title may mislead readers, particularly those in plant pathology, agrochemical science, or resistance management. The core message of the study is the discovery of melanin-independent infection strategies, which represents a paradigm shift relative to established dogma. A title reflecting this conceptual advance, rather than fungicide resistance, would be more accurate.

//Author reply:

In accordance with the reviewer's comments, we changed the title to "Breaking dependence on melanisation imparts diversity to a dogmatic invasion strategy of phytopathogenic fungi" in the revised manuscript (Lines 1–2).

//Reviewer 1 comment:

2. Conceptual alignment between the title, Introduction, and main conclusions

The final sentence of the Introduction states:

"In the present study, we identified and characterized a novel group of Colletotrichum fungi that can invade plants via nonmelanised appressorium-mediated entry (NMAE) and rediscovered HTE-type strains."

This sentence accurately captures the primary contribution of the work. However, it does not align well with the emphasis on "MBI-resistant" pathogens suggested by the title.
Similarly, the statement:
"Our findings challenge the prevailing notion that appressorial formation and melanization are universally essential for plant infection."
appears overly broad. The findings are highly significant for Colletotrichum and possibly Pyricularia, but do not justify a generalization to all plant pathogenic fungi. The authors should narrow this claim to the taxa actually examined.

//**Author reply:**
In accordance with the reviewer's comments, we changed the title to "Breaking dependence on melanisation imparts diversity to a dogmatic invasion strategy of phytopathogenic fungi" in the revised manuscript (Lines 1–2).
We also narrowed our claim and revised the sentence in the revised manuscript as follows:
"Our findings challenge the prevailing notion that appressorial formation and melanisation are universally essential for plant infections by *Colletotrichum*." (lines 109–111).

//Reviewer 1 comment:
3. Experimental framing and ecological relevance of MBI-related arguments
Several sections of the manuscript discuss the potential emergence and risk of MBI-resistant pathogens in agricultural ecosystems. However:
• MBI compounds are not applied to crops affected by Colletotrichum.
• The experimental systems used do not reflect the systemic application, field-level exposure, or selective pressures associated with real-world fungicide use.
• If the authors wish to argue ecological or agronomic risk, experimental designs explicitly addressing these contexts would be required.
Otherwise, the discussion should be reframed to avoid overstating real-world resistance risks.

//**Author reply:**
In accordance with the reviewer's comments, we reframed the style of writing to avoid overstating. We deleted the sentence "More importantly, in paddy field under agrochemical disease control using MBIs, rice may remain susceptible to NMAE-based invasion." (Line 393). We also revised the sentences in the revised manuscript as follow:
"These phenomena indicate that, in addition to carbohydrates, specific agrochemicals may act as factors that induce appressorium-independent, HTE-related morphogenesis and the subsequent plant invasion via HTE by specific *Colletotrichum* strains, although MBIs are not applied to crops affected

by *Colletotrichum*.” (Lines 412–416).
“However, the progression of faint lesions was restricted and assumed to be a hypersensitive-like response in rice (Supplementary Fig. 14e), which was consistent with the well-known incompatibility of *Colletotrichum* fungi with rice. Therefore, at present, it is unlikely that NMAE-type and polyphagous *Colletotrichum* strains in the acutatum SC will become fully adapted to rice and pose a serious threat.” (Lines 543–548).

//Reviewer 1 comment:
4. Interpretation of “in nature” and field relevance
The phrase “NMAE-based plant infection in nature” (Line 253) is problematic. The data are derived from controlled laboratory inoculation assays, not field studies. This wording could be misinterpreted as evidence for natural infection dynamics. A more precise phrasing (e.g., “across multiple species” or “in diverse Colletotrichum lineages”) would be preferable.

//**Author reply:**
In accordance with the reviewer’s comment, we deleted the phrase “in nature” and used the phrase “Spontaneous NMAE” (Lines 269, 289, and 1038). We replaced the phrase “natural conditions” to “MBI-free conditions” (Lines 273 and 488). We also revised the sentences in the revised manuscript as follow:
“Collectively, these results indicate that *Cfio* HF3 naturally follows melanisation-independent strategies to infect plants.” (Lines 298–299).

//Reviewer 1 comment:
5. Evolutionary and mechanistic implications within the C. acutatum group
The C. acutatum species complex is known for:
• broad host range,
• high aggressiveness,
• relative tolerance to chemical control.
The data presented (e.g., Fig. 4a, Supplementary Fig. 13) suggest that NMAE-type strains may possess fundamentally different infection programs, potentially relying more heavily on enzymatic cell wall degradation or secondary metabolites.
Notably, within this clade, species such as C. orchidophilum and C. fioriniae lack the tandem Perox–AlcOx metalloenzyme system, which chemically remodels host cuticular components into signaling molecules required for proper appressorium development (Bissaro et al., Sci. Adv., 2022).
Given known differences from C. orbiculare, including loss of conserved tandem gene clusters involved in plant surface signal perception, the authors are encouraged to discuss whether

appressorium development, polarity establishment, and cell-cycle coupling may be qualitatively distinct in this group. This perspective would significantly strengthen the mechanistic and evolutionary interpretation of the findings.

Please also see section 7.

//Author reply:

As the reviewer suggested, we added the following sentences in the Discussion section with the following references:

"Alternatively, acutatum SC may qualitatively rewire infection-related developmental programmes compared to conventional *Colletotrichum* fungi. Consistent with this idea, acutatum SC and its closely-related species *C. orchidophilum* lack the tandem Perox–AlcOx metalloenzyme system that chemically remodels host cuticular components into signalling molecules required for proper appressorium development[61]. However, except for appressorial pigmentation, MAE- and NMAE-type strains do not exhibit evident morphological differences." (Lines 476–482).

"In *C. orbiculare*, a model MAE-type pathogen, appressorium morphogenesis is tightly integrated with chemical surface sensing, polarity establishment, and cell-cycle control. This integration is mediated through conserved pathways, such as the MOR/NDR kinase cascade[62] and the Bub2–Bfa1–Tem1 module, which regulates G1/S progression during infection-related morphogenesis[63]. In contrast, the alternative entry modes of the acutatum SC, including NMAE and HTE, combined with the absence of chemical gating mechanisms (e.g., Perox–AlcOx), suggest that these fungi may employ alternative combinations of morphogenetic timing, polarity control, and host surface engagement to achieve rapid and effective penetration." (Lines 492–501).

References

<u>Added:</u>

61. Bissaro, B. et al. Tandem metalloenzymes gate plant cell entry by pathogenic fungi. *Sci. Adv.* **8**, eade9982 (2022).

62. Kodama, S. et al. The morphogenesis-related NDR kinase pathway of *Colletotrichum orbiculare* is required for translating plant surface signals into infection-related morphogenesis and pathogenesis. *PLoS Pathog.* **13**, e1006189 (2017).

63. Fukada, F. & Kubo, Y. *Colletotrichum orbiculare* regulates cell cycle G1/S progression via a two-component GAP and a GTPase to establish plant infection. *Plant Cell* **27**, 2530–44 (2015).

//Reviewer 1 comment:

6. Consideration of Agrochemical Usage and Societal Implications

Line 470: The discussion suggesting that melanin biosynthesis inhibitors may induce or promote

hyphal tip–based entry (HTE)–type infection warrants careful reconsideration in light of real-world agrochemical usage and regulatory context. Pyroquilone and related MBI-D fungicides are registered and applied exclusively for the control of rice blast caused by Pyricularia oryzae. To date, there is no experimental or field-based evidence demonstrating that MBI application induces HTE-type invasion in Pyricularia, nor that such compounds alter infection strategies under agronomically relevant conditions.

In contrast, HTE-type infection described in this study has been demonstrated only in Colletotrichum species, for which melanin biosynthesis inhibitors are not used in agricultural practice. As currently phrased, the discussion may be interpreted as implying a fungicide-driven emergence of alternative infection strategies in crop systems, a claim that carries potential societal and regulatory implications. In the absence of direct supporting evidence, the authors are encouraged to substantially tone down this argument, clearly delineate laboratory observations from field-relevant scenarios, or explicitly frame this idea as speculative rather than established risk.

//Author reply:

We agree with the reviewer; we also think the pyroquilon-driven emergence of alternative infection strategy is a careful issue. First of all, to strengthen the validity of pyroquilon-induced HTE, we performed additional experiments (New Fig. 8). They include dose-dependent effects of pyroquilon on the lesion formation by *Ctro* Ishigaki-Banana2 (New Fig. 8b), quantification of pyroquilon-induced infection-related morphogenesis on *Arabidopsis* (New Fig. 8d) and sugar-induced infection-related morphogenesis of the same pathogen (New Fig. 8e), and pathogenicity assay on the host banana fruits (New Fig. 8f) with microscopic observations (New Fig. 8g). We also added the microscopic images of nonmelanised appressoria of *Ctro* Ishigaki-Banana2 on *Arabidopsis* in the presence of pyroquilon (New Fig. 8c). Quantification assay strongly supported the pyroquilon-specific induction of HTE in *Ctro* Ishigaki-Banana2 on *Arabidopsis* (New Fig. 8d). We additionally found that *Ctro* Ishigaki-Banana2 was also a putative sugar-induced HTE-type pathogen (New Fig. 8e). More importantly, we revealed that this pathogen showed HTE-type morphogenesis and exhibited pathogenicity on host banana fruits irrespective of the MBI treatments (New Fig. 8f, g). Based on these additional results, we toned down the expression and mentioned that, at present, it is not necessary to consider the adverse side effects of MBI-R on *Colletotrichum* infection strategies in agricultural practice. In accordance with the reviewer's comments, we also added the description about the low risk of the emergence of MBI-resistant *P. oryzae* strain by the MBI treatments. On the other hand, as the reviewer also suggested, we explicitly phrased the risk of the adverse side effects of pyroquilon as speculative idea.

We drastically revised the Result section regarding to pyroquilon-induced HTE as follows:
"In contrast to MBI-D[31–33], *P. oryzae* strains resistant to MBI-R fungicides have not emerged, despite several decades of continuous use[57]. Therefore, the potential risk for resistance development to MBI-R fungicides is estimated to be low. Intriguingly, we found that *Ctro* Ishigaki-Banana2 exhibited specific insensitiveness to MBI-R pyroquilon in the lesion production on *A. thaliana* immunocompromised mutants, whereas other MBIs, such as carpropamid and tolprocarb, remained effective (Fig. 8a). Another MBI-R, fthalide, strongly prevented the *Ctro* Ishigaki-Banana2-induced lesion formation in *A. thaliana*, in contrast with the dose-dependent lesion-producing effects of pyroquilon (Fig. 8b). Specifically, in the presence of carpropamid or tolprocarb, appressoria formed on the plant surface were arrested at the premelanisation stage and could not penetrate plant epidermis (Fig. 8c). Therefore, *Ctro* Ishigaki-Banana2 was classified as a conventional MAE-type pathogen (Fig. 4b). In contrast, *Ctro* Ishigaki-Banana2 unexpectedly exhibited suppressed appressorial formation, germ tube-like hyphal extension and HTE-based invasive hyphal development in the presence of pyroquilon, while melanisation of partly formed appressoria was sufficiently prevented (Fig. 8c, d). Therefore, *Ctro* Ishigaki-Banana2 was characterised as pyroquilon-induced HTE-type (Fig. 4b). Moreover, this pathogen was classified as a putative sugar-induced HTE-type strain (Fig. 8e). These phenomena indicate that, in addition to carbohydrates, specific agrochemicals may act as factors that induce appressorium-independent, HTE-related morphogenesis and the subsequent plant invasion via HTE by specific *Colletotrichum* strains, although MBIs are not applied to crops affected by *Colletotrichum*. On the contrary, the virulence of *Ctro* Ishigaki-Banana2 on ripe fruits of its host banana was not affected by treatments with any of the investigated MBIs (Fig. 8f). *Ctro* Ishigaki-Banana2 consistently exhibited HTE-type morphogenesis on the host surface regardless of the presence or absence of MBIs, suggesting host infection via HTE induced by sugar and/or other ripe fruit-derived signal(s) (Fig. 8g)." (Lines 396–421).

We also revised the sentences in the relevant part of the Discussion section as follows:
"The MBI-R compound, pyroquilon, induced appressorium-independent HTE-related morphogenesis, HTE, and consequent pyroquilon-insensitive lesion production on *Arabidopsis* in *Ctro* Ishigaki-Banana2 (Fig. 8). Therefore, we speculate that pyroquilon may increase fungal virulence by inducing a more invasive entry mode in the specific plant–fungus interaction. However, *Ctro* Ishigaki-Banana2 develops HTE-type morphogenesis and causes subsequent necrotic lesion formation on the host banana, irrespective of the MBI treatments (Fig. 8f, g). As MBIs are not used and do not exert selection pressure on *Colletotrichum* species in the field, pyroquilon-induced HTE might be incidentally acquired. Therefore, at present, no consideration is required for the adverse side effects of MBI-R on *Colletotrichum* infection strategies in agricultural practice. Although MAE-

type *P. oryzae* also employs an appressorium-independent invasion strategy for rice root colonisation, where the penetration occurs via HTE-like hyphal swelling[66], typical foliar infection is tightly linked to MAE-based invasion. This strong dependency explains why *P. oryzae* has not yet developed resistance to MBI-R fungicides[57]; there is no evidence to support NMAE-based invasion or MBI-induced HTE-like invasion in *Pyricularia* fungi to date." (Lines 550–565)

Reference

Added:

66. Sesma, A. & Osbourn, A. E. The rice leaf blast pathogen undergoes developmental processes typical of root-infecting fungi. *Nature* **431**, 582–586 (2004).

//Reviewer 1 comment:

7. Suggested Perspective: Evolutionary Remodeling of Infection Programs in the *Colletotrichum acutatum Clade*

Findings presented in this study strongly suggest that infection strategies within the Colletotrichum acutatum clade cannot be fully interpreted as simple variants of the canonical melanized appressorium-mediated entry paradigm established for C. orbiculare. Given the reported divergence of this clade, including the loss of conserved gene clusters implicated in plant surface signal perception and the absence of the tandem Perox–AlcOx metalloenzyme system that chemically remodels host cuticular components (Bissaro et al., Sci. Adv., 2022). The data presented in this manuscript are more consistent with a qualitative rewiring of infection-related developmental programs rather than with partial relaxation of melanization-dependent mechanisms.

In C. orbiculare, appressorium morphogenesis is tightly integrated with chemical surface sensing, polarity establishment, and cell-cycle control through conserved pathways such as the MOR/NDR kinase cascade (Kodama et al., PLoS Pathog., 2017) and the Bub2–Bfa1–Tem1 module regulating G1/S progression during infection-related morphogenesis (Fukada & Kubo, Plant Cell, 2015). By contrast, the diversity of strategies observed within the acutatum lineage, including nonmelanized appressorium-mediated entry (NMAE), hyphal tip–based entry (HTE), and the presence or absence of chemical gating mechanisms such as Perox–AlcOx, suggests that these fungi may deploy alternative combinations of morphogenetic timing, polarity control, and host-surface engagement to achieve rapid and effective penetration.

I therefore suggest that the authors explicitly interpret NMAE not merely as exceptions to a melanization-centered dogma, but as outcomes of evolutionary diversification of infection programs within Colletotrichum.

//Author reply:

In accordance with the reviewer’s comments, we added the following sentences and references in the Discussion section:

“Alternatively, acutatum SC may qualitatively rewire infection-related developmental programmes compared to conventional *Colletotrichum* fungi. Consistent with this idea, acutatum SC and its closely-related species *C. orchidophilum* lack the tandem Perox–AlcOx metalloenzyme system that chemically remodels host cuticular components into signalling molecules required for proper appressorium development[61]. However, except for appressorial pigmentation, MAE- and NMAE-type strains do not exhibit evident morphological differences.” (Lines 476–482).

“In *C. orbiculare*, a model MAE-type pathogen, appressorium morphogenesis is tightly integrated with chemical surface sensing, polarity establishment, and cell-cycle control. This integration is mediated through conserved pathways, such as the MOR/NDR kinase cascade[62] and the Bub2–Bfa1–Tem1 module, which regulates G1/S progression during infection-related morphogenesis[63]. In contrast, the alternative entry modes of the acutatum SC, including NMAE and HTE, combined with the absence of chemical gating mechanisms (e.g., Perox–AlcOx), suggest that these fungi may employ alternative combinations of morphogenetic timing, polarity control, and host surface engagement to achieve rapid and effective penetration.” (Lines 492–501).

References

<u>Added:</u>

61. Bissaro, B. et al. Tandem metalloenzymes gate plant cell entry by pathogenic fungi. *Sci. Adv.* **8**, eade9982 (2022).

62. Kodama, S. et al. The morphogenesis-related NDR kinase pathway of *Colletotrichum orbiculare* is required for translating plant surface signals into infection-related morphogenesis and pathogenesis. *PLoS Pathog.* **13**, e1006189 (2017).

63. Fukada, F. & Kubo, Y. *Colletotrichum orbiculare* regulates cell cycle G1/S progression via a two-component GAP and a GTPase to establish plant infection. *Plant Cell* **27**, 2530–44 (2015).

//Reviewer 1 comment:

Minor Comments

• Line 124 and elsewhere: The term hyaline may be misleading; hyaline means non-visible, colorless or non-melanized would be more precise.

//Author reply:

In accordance with the reviewer’s comment, we have replaced the word “hyaline” to

"nonmelanised" throughout the manuscript (Lines 132, 142, 149, 430, 465, 571, and 573).

//Reviewer 1 comment:

• Line 140: The term albino is typically annotated for PKS-deficient DHN-melanin mutants. Consider alternative wording.

//**Author reply:**

Although previous report annotated the term "albino" for Δ*scd1* mutants (Wang et al., 2020), to avoid confusion, we deleted the word "albino" and used "melanin-deficient" throughout the manuscript (Lines 69, 82, 91, 147, 166, 224, 375, and 974).

Reference

21. Wang, T. et al. CgSCD1 is essential for melanin biosynthesis and pathogenicity of *Colletotrichum gloeosporioides*. *Pathogens* **9**, 141 (2020).

//Reviewer 1 comment:

• Line 149: "MBI-resistant" may be better replaced with "MBI-insensitive" or rephrased.

//**Author reply:**

In accordance with the reviewer's comment, we have replaced the word "MBI-resistant" to "MBI-insensitive" or "melanisation-independent" throughout the manuscript (Lines 114, 159, 243, 245–246, 296, 589, 948, 1301, 1371, and 1378).

//Reviewer 1 comment:

• Line 318: Fig. 5i is cited but appears to be missing.

//**Author reply:**

As the reviewer pointed out, we corrected the figure number into Fig. 5g (Line 334).

//Reviewer 1 comment:

• Line 360: Clarify whether the discussion refers to Colletotrichum or Pyricularia. If the latter, direct evidence of NMAE in Pyricularia would be required.

//**Author reply:**

We agree with the reviewer. To avoid confusion, we deleted the following sentence in the revised manuscript.

“More importantly, in paddy field under agrochemical disease control using MBIs, rice may remain susceptible to NMAE-based invasion.” (line 393).

//Reviewer 1 comment:

Overall Assessment

This manuscript reports a genuinely important and conceptually novel finding: that melanization is not universally required for appressorium-mediated plant invasion in Colletotrichum. The work challenges a long-standing dogma and is supported by extensive experimental evidence.

However, substantial revision is needed to:

- *correct potentially misleading terminology,*
- *align the title and conclusions with the actual data,*
- *moderate claims regarding ecological emergence and fungicide resistance,*
- *and deepen the mechanistic discussion within an evolutionary framework.*

With these revisions, the manuscript would represent a strong and influential contribution to the field.

Strengths of the Manuscript

- *Discovery of melanin-independent appressorium-mediated infection in the C. acutatum group.*
- *Rigorous experimental analysis using Arabidopsis immune-deficient mutants.*
- *Willingness to challenge an entrenched paradigm in fungal plant pathology.*
- *Insight into qualitative differences in host immune responses depending on infection mode.*

//Author reply:

Thank you again for your critical reading of our manuscript and your insightful suggestions and positive comments.

Finally, we reiterate our thanks to Reviewer 1 for improving our manuscript.

Responses to reviewer 2:

//Reviewer 2 comment:

Reviewer #2 (Remarks to the Author):

The manuscript describes the existence of a melanin-independent infection mode in Colletotrichum fungi—non-melanized appressorium-mediated entry (NMAE). Strains with this mode exhibit resistance to melanin biosynthesis inhibitors (MBIs) and rapidly penetrate plant tissues via mature non-melanized appressorium. Additionally, strains relying on hyphal tip entry (HTE) were identified, indicating that appressorial melanization is not universally essential. These findings challenge the conventional view that melanization of appressorium is a prerequisite for plant infection and underscore the necessity of considering the emergence of NMAE-type and HTE-type pathogens. However, a major revision is still needed before considering the acceptance.

//**Author reply:**

Thank you for your critical reading of our manuscript and your insightful suggestions and positive comments, which have helped us to improve our manuscript considerably. Based on the comments provided by the reviewers, we revised our manuscript after performing new experiments. We have addressed all of the reviewers' concerns. Please see our point-by-point responses.

//Reviewer 2 comment:

1. Colletotrichum fungi have a broad host range. Given that the plants inoculated in the manuscript were mostly herbaceous, should woody plants be included as controls to verify the generalizability of the experimental results?

//**Author reply:**

In the original manuscript, our inoculated plants included both herbaceous (*A. thaliana*, cucumber, cosmos, hyacinth, spinach, cockscomb, maize, sorghum, and rice) and woody plants (apple, Japanese flowering cherry, and Japanese ivy). However, as the reviewer pointed out, the numbers of tested woody plants were little smaller than that of herbaceous plants. In accordance with the reviewer's comment, to verify the generalizability of newly discovered MBI-insensitive invasion mode of *Colletotrichum* fungi for broad types of plants, we performed additional inoculation assays on many woody plants (New Supplementary Fig. 15). In the face of difficulties of seasonal deciduous trees, we managed to prepare the leaves of sacred bamboo wild-type and cv. 'Otafukunanten', Chinese mahonia, loquat, olive, Japanese spindle tree, Japanese andromeda, Japanese holly, Chinese holly, camellia, tea tree, kumquat, Japanese summer orange, and gardenia,

and inoculated three NMAE-type *Colletotrichum* strains on them in the presence or absence of melanin biosynthesis inhibitor (MBI) CAR. Of those, the results of sacred bamboo wild-type and cv. 'Otafukunanten', Japanese andromeda, and Japanese summer orange, which showed susceptibility (with regards to lesion formation) to inoculated pathogens in the absence of CAR, were added as new Supplementary Fig. 15. NMAE-type strains could induce MBI-insensitive lesions on the leaves of sacred bamboo wild-type and cv. 'Otafukunanten' and Japanese summer orange, whereas it exhibited melanisation dependency on the leaf of Japanese andromeda (New Supplementary Fig. 15). Based on all our results (New Supplementary Fig. 14 and 15), the MBI-insensitive lesion formation of NMAE-type pathogens has no correlation with herbaceous- or woody-type of the inoculated plants. Therefore, we added the following sentences in the revised manuscript:
"To verify generalisability, we performed inoculation assays of NMAE-type strains on woody plants and obtained similar results. Specifically, even in the presence of MBI, *Cfio* CC1 produced lesions on sacred bamboo wild-type and cv. Otafukunanten, and *Cfio* S96a1 and *Cgod* MC1 formed lesions on Japanese summer orange, whereas lesion formation by *Cfio* CC1 on Japanese andromeda was prevented by MBI (Supplementary Fig. 15)." (Lines 384–389).
"The same is true of woody plants; Japanese andromeda prevented lesion formations of NMAE-type pathogens, whereas sacred bamboo and Japanese summer orange did not (Supplementary Fig. 15)." (Lines 512–514).

//Reviewer 2 comment:
2. Does Pyricularia fungi also exhibit a melanin-independent infection mode?

//Author reply:
Only in underground tissues (roots) of rice, not in aboveground tissues, it was reported from the controlled laboratory inoculation assays that *Pyricularia oryzae* could invade and infect host rice via appressorium-independent HTE-like invasion mode (Sesma et al., 2004). In agricultural field, if the MBI-resistant *P. oryzae* emerged, it should become a great important issue in a moment. However, known reports on MBI-resistant *P. oryzae* in field study just demonstrated the failure of the specific MBI to block appressorial melanisation (Sawada et al., 2004; Takagaki et al., 2004; Yamada et al., 2004). In short, there is nothing in an example for identifying NMAE- or HTE-type *P. oryzae* in paddy field, where farmers were likely always conducting practical monitoring for the MBI-resistant strains. As another reviewer also pointed out, MBI-induced HTE of *P. oryzae* has not been reported. Therefore, in answer to your query, and in accordance with another reviewer's comment, we clearly mentioned above contents and toned down the expression regarding to the risk of emergence of MBI-resistant *P. oryzae*. The substantially revised sentences in the discussion section with the additional reference (Sesma et al., 2004) is as follows:

“Although MAE-type *P. oryzae* also employs an appressorium-independent invasion strategy for rice root colonisation, where the penetration occurs via HTE-like hyphal swelling[66], typical foliar infection is tightly linked to MAE-based invasion. This strong dependency explains why *P. oryzae* has not yet developed resistance to MBI-R fungicides[57]; there is no evidence to support NMAE-based invasion or MBI-induced HTE-like invasion in *Pyricularia* fungi to date.” (Lines 560–565).

References

31. Sawada, H., Sugihara, M., Takagaki, M. & Nagayama, K. Monitoring and characterization of Magnaporthe grisea isolates with decreased sensitivity to scytalone dehydratase inhibitors. *Pest. Manag. Sci.* **60**, 777–785 (2004).
32. Takagaki, M. et al. Mechanism of resistance to carpropamid in Magnaporthe grisea. *Pest. Manag. Sci.* **60**, 921–926 (2004).
33. Yamada, N. et al. Enzymatic characterization of scytalone dehydratase Val75Met variant found in melanin biosynthesis dehydratase inhibitor (MBI-D) resistant strains of the rice blast fungus. *Biosci. Biotechnol. Biochem.* **68**, 615–621 (2004).

Added:

66. Sesma, A. & Osbourn, A. E. The rice leaf blast pathogen undergoes developmental processes typical of root-infecting fungi. *Nature* **431**, 582–586 (2004).

//Reviewer 2 comment:

3. How does the non-melanized appressorium-mediated entry (NMAE) mode maintain turgor in Colletotrichum appressorium?

//Author reply:

As the reviewer pointed out, we also think the validity of the link between appressorial melanisation and turgor generation is an important issue. First, we replaced the preprint-based reference “Kumakura et al., bioRxiv” to recently published one in *Science* “Kumakura et al., 2026”. In this paper, it is reported that two dihydroxyhexanoic acid (DHHA) polymer synthase genes, which are conserved among phytopathogenic fungi, are strongly contributed to the direct generation of appressorial turgor. Kumakura et al. also demonstrated that appressorial melanisation is not directly necessary for the cell wall permeability (pore size) for osmolytes, rather contributes to the cell wall rigidity (Kumakura et al., 2026). In fact, the stiffness of appressorial cell wall in the albino mutants (Δ*pks1*) of *Corb* were greatly lower than that in wild-type (Kumakura et al., 2026). This is consistent with our results of *Corb* in evaluation assays for the appressorial resistance against external cell wall-degrading enzymes (Fig. 3c and Supplementary Fig. 6c). Based on the results obtained from five *Colletotrichum* species, *Cgra*, *Cglo*, *Chig*, *Csia*, and *Cfio*, in the previous reports (Ludwig et al.,

2014; Wang et al., 2020) and our study (Fig. 3), along with the recent findings by Kumakura et al. (Kumakura et al., 2026), it is suggested that many *Colletotrichum* strains do not primarily require melanisation for appressorial turgor generation, whereas only well-studied model pathogens *Corb* and likely *P. oryzae* exhibited malanisation-dependent characteristics in turgor evaluation assays. To support this idea, we additionally performed turgor evaluation assays of *P. oryzae* and another two *Colletotrichum* species, *Cnym* and *Cgod*, in the presence or absence of CAR (New Supplementary Fig. 7). As expected, we confirmed that nonmelanised appressoria of *P. oryzae* exhibited melanisation-dependent turgor and/or cell wall rigidity in appressoria, whereas the appressoria of *Cnym* and *Cgod* showed melanisation independency (New Supplementary Fig. 7). These melanisation-independent turgor generation in most *Colletotrichum* species is consistent with the previous reports on other, not *Colletotrichum*, nonmelanised appressorium-type (Chang et al., 2014) and protoappressorium-type (HTE-like) (Müller et al., 2024) phytopathogens, both of which generated turgor pressure without melanisation. Altogether, this issue has been discussed as basic characteristics among turgor-generating appressoria of phytopathogens, not specific to that of *Colletotrichum* NMAE-type appressoria; it is likely that many phytopathogenic fungi regulate semipermeability of appressorial cell wall without relying on the melanisation. Based on the above, we substantially revised the manuscript and added the new results as Supplementary Fig. 7. The revised sentences are as follows:

"First, it serves as a semipermeable barrier retains osmolytes while allowing water influx, thereby generating high turgor pressure for host penetration[12–14]; however, dihydroxyhexanoic acid (DHHA) polymers have recently been identified as genuine factors that reduce cell wall porosity for semipermeability[15]." (Lines 59–63).

"The identification of the semipermeability control by DHHA polymers resolved the contradiction between melanisation and turgor generation; the melanin layer provides cell wall rigidity to withstand high turgor pressure[15]." (Lines 96–99).

"Consistent with previous reports[13,52], the nonmelanised appressoria of *Corb* 104-T exhibited reduced resistance to external high osmotic stress, suggesting melanisation-dependent turgor generation and/or cell wall rigidity in the appressoria[15] (Fig. 3a). Notably, similar to *Cgra* CgM2[20] and *Cglo* EX2016-02[21], the appressoria of *Chig* Abr1-5, *Csia* MAF1, and *Cfio* CC1 generated sufficient turgor pressure, regardless of melanisation (Fig. 3a). The results of these five *Colletotrichum* species were consistent with the recent finding that DHHA polymers, but not melanin, directly contributed to the pore size reduction of appressorial cell wall and consequent turgor generation[15]. We also confirmed the melanisation dependency of *P. oryzae* in the same appressorial turgor evaluation assay (Supplementary Fig. 7a). Collectively, only the data of the well-studied *Corb* 104-T and *P. oryzae* aligned with the long-accepted theory on melanisation-dependent turgor and/or cell wall rigidity in appressoria (Fig. 3a and Supplementary Fig. 7a). To further

strengthen the notion that the appressorial turgor and/or cell wall rigidity in most *Colletotrichum* fungal strains is essentially melanisation-independent, we evaluated the turgor of another two *Colletotrichum* strains belonging to different species (Supplementary Fig. 7b). As expected, the appressoria of *Colletotrichum nymphaeae* (*Cnym*) GCP26 and *Colletotrichum godetiae* (*Cgod*) MC1 exhibited melanisation-independent turgor generation (Supplementary Fig. 7b)." (Lines 177–194).
"Notably, the nonmelanised appressoria of *Corb* 104-T moderately collapsed even in the absence of LEs and further collapsed by subsequent LE application, suggesting further decreased cell wall rigidity compared to other strains (Fig. 3c and Supplementary Fig. 6c). We did not detect any difference in CWDE resistance between melanised and nonmelanised appressoria in *Csia* MAF1 and *Cfio* CC1 (Fig. 3c and Supplementary Fig. 6c)." (Lines 212–217).
"In a recent study, Kumakura et al. demonstrated that, instead of the melanin biosynthesis gene *PKS1*, DHHA polymer biosynthesis genes *PKS2* and *PBG13* conserved across multiple fungal species in Ascomycota, directly reduce appressorial cell wall porosity in *Colletotrichum* fungi. These findings suggest that melanisation is not primarily required for turgor generation[15]. Notably, the Ascomycota fungus *Botrytis cinerea* produces a slightly swollen, nonmelanised germ tube apex called a protoappressorium and penetrates plants with turgor generation[60]; this is consistent with the presence of the *PKS2* and *PBG13* gene pair in *B. cinerea*[15]. Instead, Kumakura et al. indicated that melanin accumulation appeared to increase the physical rigidity of the appressorial cell wall, as evidenced by reduced stiffness in the Δ*pks1* mutant of *Corb*[15]. Notably, in our turgor evaluation assays, *Corb* 104-T exhibited a higher rate of collapsed or cavitated appressoria than other *Colletotrichum* fungi, including MAE- and NMAE-type strains, likely owing to low cell wall rigidity (Fig. 3a, b). Another fungal phytopathogen, *Phakopsora pachyrhizi*, develops a nonmelanised appressorium but generates high turgor pressure in a melanin-independent manner[59]. However, we found that the genome of *P. pachyrhizi* likely lacked the *PKS2* and *PBG13* gene pair as in the case with other Basidiomycota fungi[15], implying an alternative mechanism for turgor generation. Nevertheless, melanisation remains essential for appressorium-mediated plant invasion of *Colletotrichum* fungi except for NMAE-type strains (Fig. 4). The penetration ability of *Corb* into cellulose membrane occurs independently of turgor but depends on melanisation[15], highlighting the importance of concerted invasion strategies employing various melanisation-dependent and -independent appressorial functions. While the degree of dependence in each function varies among *Colletotrichum* strains, NMAE-type strains in acutatum SC may achieve almost full functionality for appressorial penetration (Supplementary Fig. 8)." (Lines 452–476).

We also deleted the results of solute exclusion assays (Original Supplementary Fig. 7) and related sentences as follows, because Kumakura et al. demonstrated the noninvolvement of appressorial melanisation in the pore size (porosity) determination, and also pointed out unreliability of solute

exclusion technique-based porosity measurements in case incubated nonmelanised appressoria exhibited abnormal shape (Kumakura et al., 2026).

"To investigate the osmolyte impermeability of appressorial cell walls under melanisation inhibition, we performed a solute exclusion assay using polyethylene glycols of different molecular sizes (Supplementary Fig. 7). Replicating previous research[47], we confirmed that the appressoria of the low-melanisation mutants of *Corb* 104-T (*thr4*) were more permeable than those of the wild-type, likely due to altered appressorial cell wall porosity (Supplementary Fig. 7a). Similar results were obtained with the albino mutants of *Csia* MAF1 and *Cfio* CC1, suggesting porosity similar to that of the appressorial cell wall of *Corb* 104-T; however, they generated high turgor pressure in a melanisation-independent manner (Supplementary Fig. 7b, c)." (Line 194).

References

<u>Deleted:</u>

51. Kumakura, N. et al. Dihydroxyhexanoic acid biosynthesis controls turgor in pathogenic fungi. *bioRxiv* 606736, Preprint at https://doi.org/10.1101/2024.08.07.606736 (2024).

<u>Added:</u>

15. Kumakura, N. et al. Dihydroxyhexanoic acid biosynthesis controls turgor in pathogenic fungi. *Science* **391**, 700–706 (2026).

60. Müller, T. et al. Plant infection by the necrotrophic fungus Botrytis requires actin-dependent generation of high invasive turgor pressure. *New Phytol.* **244**, 192–201 (2024).

Finally, we reiterate our thanks to Reviewer 2 for improving our manuscript.

Responses to reviewer 3:

//Reviewer 3 comment:

Reviewer #3 (Remarks to the Author):

This manuscript challenges the long-standing dogma in plant pathology that melanized appressoria are universally essential for host penetration by identifying and characterizing two alternative invasion strategies in Colletotrichum fungi: nonmelanized appressorium-mediated entry (NMAE) and hyphal tip-based entry (HTE). The authors found that NMAE-type strains, which form a rapidly expanding monophyletic group, can invade hosts earlier and independently of melanization. The work employs a combination of phenotypic, phylogenetic, and functional assays, supporting its conclusion that fungal invasion strategies are more diverse than previously recognized. However, this study primarily focuses on the discovery and description of NMAE and HTE phenomenon, with limited exploration of their underlying mechanisms.

//**Author reply:**

Thank you for your critical reading of our manuscript and your insightful suggestions and positive comments, which have helped us to improve our manuscript considerably. Based on the comments provided by the reviewers, we revised our manuscript after performing new experiments. We have addressed all of the reviewers' concerns. Please see our point-by-point responses.

//Reviewer 3 comment:

1. The title/abstract focuses on Colletotrichum, yet the introduction pairs it with Pyricularia. Are the findings specific to Colletotrichum, or is the paradigm shift suggested to be broader? The manuscript also lacks findings related to NMAE or HTE in Pyricularia.

//**Author reply:**

As the reviewer pointed out, our findings are paradigm shift specific to *Colletotrichum*, therefore, as the other reviewer suggested, we explicitly stated it in the revised manuscript as follows:
"Our findings challenge the prevailing notion that appressorial formation and melanisation are universally essential for plant infections by *Colletotrichum*." (Lines 109–111).

Only in underground tissues (roots), not in aboveground tissues, it was reported from the controlled laboratory inoculation assays that *Pyricularia oryzae* could invade and infect host rice via appressorium-independent HTE-like invasion mode (Sesma et al., 2004). In agricultural field, if the MBI-resistant *P. oryzae* emerged, it should become a great important issue in a moment. However,

known reports on MBI-resistant *P. oryzae* in field study just demonstrated the failure of the specific MBI to block appressorial melanisation (Sawada et al., 2004; Takagaki et al., 2004; Yamada et al., 2004). In short, there is nothing in an example for identifying NMAE- or HTE-type *P. oryzae* in paddy field, where farmers were likely always conducting practical monitoring for the MBI-resistant strains. As another reviewer also pointed out, MBI-induced HTE of *P. oryzae* has not been reported. Therefore, in answer to your query, and in accordance with another reviewer's comment, we clearly mentioned above contents and toned down the expression regarding to the risk of emergence of MBI-resistant *P. oryzae*. The substantially revised sentences in the discussion section with the additional reference (Sesma et al., 2004) is as follows:

"Although MAE-type *P. oryzae* also employs an appressorium-independent invasion strategy for rice root colonisation, where the penetration occurs via HTE-like hyphal swelling[66], typical foliar infection is tightly linked to MAE-based invasion. This strong dependency explains why *P. oryzae* has not yet developed resistance to MBI-R fungicides[57]; there is no evidence to support NMAE-based invasion or MBI-induced HTE-like invasion in *Pyricularia* fungi to date." (Lines 560–565).

References

31. Sawada, H., Sugihara, M., Takagaki, M. & Nagayama, K. Monitoring and characterization of Magnaporthe grisea isolates with decreased sensitivity to scytalone dehydratase inhibitors. *Pest. Manag. Sci.* **60**, 777–785 (2004).
32. Takagaki, M. et al. Mechanism of resistance to carpropamid in Magnaporthe grisea. *Pest. Manag. Sci.* **60**, 921–926 (2004).
33. Yamada, N. et al. Enzymatic characterization of scytalone dehydratase Val75Met variant found in melanin biosynthesis dehydratase inhibitor (MBI-D) resistant strains of the rice blast fungus. *Biosci. Biotechnol. Biochem.* **68**, 615–621 (2004).

Added:

66. Sesma, A. & Osbourn, A. E. The rice leaf blast pathogen undergoes developmental processes typical of root-infecting fungi. *Nature* **431**, 582–586 (2004).

//Reviewer 3 comment:

2. A key mechanistic question is how NMAE appressoria generate sufficient turgor pressure without melanin for penetration. The study notes variation in dependence but does not elucidate the alternative mechanism(s) compensating for the lack of melanin.

//Author reply:

As the reviewer pointed out, we also think the validity of the link between appressorial melanisation and turgor generation is an important issue. First, we replaced the preprint-based reference

“Kumakura et al., bioRxiv” to recently published one in *Science* “Kumakura et al., 2026”. In this paper, it is reported that two dihydroxyhexanoic acid (DHHA) polymer synthase genes, which are conserved among phytopathogenic fungi, are strongly contributed to the direct generation of appressorial turgor. Kumakura et al. also demonstrated that appressorial melanisation is not directly necessary for the cell wall permeability (pore size) for osmolytes, rather contributes to the cell wall rigidity (Kumakura et al., 2026). In fact, the stiffness of appressorial cell wall in the albino mutants (Δ*pks1*) of *Corb* were greatly lower than that in wild-type (Kumakura et al., 2026). This is consistent with our results of *Corb* in evaluation assays for the appressorial resistance against external cell wall-degrading enzymes (Fig. 3c and Supplementary Fig. 6c). Based on the results obtained from five *Colletotrichum* species, *Cgra*, *Cglo*, *Chig*, *Csia*, and *Cfio*, in the previous reports (Ludwig et al., 2014; Wang et al., 2020) and our study (Fig. 3), along with the recent findings by Kumakura et al. (Kumakura et al., 2026), it is suggested that many *Colletotrichum* strains do not primarily require melanisation for appressorial turgor generation, whereas only well-studied model pathogens *Corb* and likely *P. oryzae* exhibited malanisation-dependent characteristics in turgor evaluation assays. To support this idea, we additionally performed turgor evaluation assays of *P. oryzae* and another two *Colletotrichum* species, *Cnym* and *Cgod*, in the presence or absence of CAR (New Supplementary Fig. 7). As expected, we confirmed that nonmelanised appressoria of *P. oryzae* exhibited melanisation-dependent turgor and/or cell wall rigidity in appressoria, whereas the appressoria of *Cnym* and *Cgod* showed melanisation independency (New Supplementary Fig. 7). These melanisation-independent turgor generation in most *Colletotrichum* species is consistent with the previous reports on other, not *Colletotrichum*, nonmelanised appressorium-type (Chang et al., 2014) and protoappressorium-type (HTE-like) (Müller et al., 2024) phytopathogens, both of which generated turgor pressure without melanisation. Altogether, this issue has been discussed as basic characteristics among turgor-generating appressoria of phytopathogens, not specific to that of *Colletotrichum* NMAE-type appressoria; it is likely that many phytopathogenic fungi regulate semipermeability of appressorial cell wall without relying on the melanisation. Based on the above, we substantially revised the manuscript and added the new results as Supplementary Fig. 7. The revised sentences are as follows:

“First, it serves as a semipermeable barrier retains osmolytes while allowing water influx, thereby generating high turgor pressure for host penetration[12–14]; however, dihydroxyhexanoic acid (DHHA) polymers have recently been identified as genuine factors that reduce cell wall porosity for semipermeability[15].” (Lines 59–63).

“The identification of the semipermeability control by DHHA polymers resolved the contradiction between melanisation and turgor generation; the melanin layer provides cell wall rigidity to withstand high turgor pressure[15].” (Lines 96–99).

“Consistent with previous reports[13,52], the nonmelanised appressoria of *Corb* 104-T exhibited

reduced resistance to external high osmotic stress, suggesting melanisation-dependent turgor generation and/or cell wall rigidity in the appressoria[15] (Fig. 3a). Notably, similar to *Cgra* CgM2[20] and *Cglo* EX2016-02[21], the appressoria of *Chig* Abr1-5, *Csia* MAF1, and *Cfio* CC1 generated sufficient turgor pressure, regardless of melanisation (Fig. 3a). The results of these five *Colletotrichum* species were consistent with the recent finding that DHHA polymers, but not melanin, directly contributed to the pore size reduction of appressorial cell wall and consequent turgor generation[15]. We also confirmed the melanisation dependency of *P. oryzae* in the same appressorial turgor evaluation assay (Supplementary Fig. 7a). Collectively, only the data of the well-studied *Corb* 104-T and *P. oryzae* aligned with the long-accepted theory on melanisation-dependent turgor and/or cell wall rigidity in appressoria (Fig. 3a and Supplementary Fig. 7a). To further strengthen the notion that the appressorial turgor and/or cell wall rigidity in most *Colletotrichum* fungal strains is essentially melanisation-independent, we evaluated the turgor of another two *Colletotrichum* strains belonging to different species (Supplementary Fig. 7b). As expected, the appressoria of *Colletotrichum nymphaeae* (*Cnym*) GCP26 and *Colletotrichum godetiae* (*Cgod*) MC1 exhibited melanisation-independent turgor generation (Supplementary Fig. 7b)." (Lines 177–194).

"Notably, the nonmelanised appressoria of *Corb* 104-T moderately collapsed even in the absence of LEs and further collapsed by subsequent LE application, suggesting further decreased cell wall rigidity compared to other strains (Fig. 3c and Supplementary Fig. 6c). We did not detect any difference in CWDE resistance between melanised and nonmelanised appressoria in *Csia* MAF1 and *Cfio* CC1 (Fig. 3c and Supplementary Fig. 6c)." (Lines 212–217).

"In a recent study, Kumakura et al. demonstrated that, instead of the melanin biosynthesis gene *PKS1*, DHHA polymer biosynthesis genes *PKS2* and *PBG13* conserved across multiple fungal species in Ascomycota, directly reduce appressorial cell wall porosity in *Colletotrichum* fungi. These findings suggest that melanisation is not primarily required for turgor generation[15]. Notably, the Ascomycota fungus *Botrytis cinerea* produces a slightly swollen, nonmelanised germ tube apex called a protoappressorium and penetrates plants with turgor generation[60]; this is consistent with the presence of the *PKS2* and *PBG13* gene pair in *B. cinerea*[15]. Instead, Kumakura et al. indicated that melanin accumulation appeared to increase the physical rigidity of the appressorial cell wall, as evidenced by reduced stiffness in the Δ*pks1* mutant of *Corb*[15]. Notably, in our turgor evaluation assays, *Corb* 104-T exhibited a higher rate of collapsed or cavitated appressoria than other *Colletotrichum* fungi, including MAE- and NMAE-type strains, likely owing to low cell wall rigidity (Fig. 3a, b). Another fungal phytopathogen, *Phakopsora pachyrhizi*, develops a nonmelanised appressorium but generates high turgor pressure in a melanin-independent manner[59]. However, we found that the genome of *P. pachyrhizi* likely lacked the *PKS2* and *PBG13* gene pair as in the case with other Basidiomycota fungi[15], implying an alternative mechanism for turgor generation. Nevertheless, melanisation remains essential for appressorium-mediated plant invasion of

Colletotrichum fungi except for NMAE-type strains (Fig. 4). The penetration ability of *Corb* into cellulose membrane occurs independently of turgor but depends on melanisation[15], highlighting the importance of concerted invasion strategies employing various melanisation-dependent and -independent appressorial functions. While the degree of dependence in each function varies among *Colletotrichum* strains, NMAE-type strains in acutatum SC may achieve almost full functionality for appressorial penetration (Supplementary Fig. 8).” (Lines 452–476).

We also deleted the results of solute exclusion assays (Original Supplementary Fig. 7) and related sentences as follows, because Kumakura et al. demonstrated the noninvolvement of appressorial melanisation in the pore size (porosity) determination, and also pointed out unreliability of solute exclusion technique-based porosity measurements in case incubated nonmelanised appressoria exhibited abnormal shape (Kumakura et al., 2026).
“To investigate the osmolyte impermeability of appressorial cell walls under melanisation inhibition, we performed a solute exclusion assay using polyethylene glycols of different molecular sizes (Supplementary Fig. 7). Replicating previous research[47], we confirmed that the appressoria of the low-melanisation mutants of *Corb* 104-T (*thr4*) were more permeable than those of the wild-type, likely due to altered appressorial cell wall porosity (Supplementary Fig. 7a). Similar results were obtained with the albino mutants of *Csia* MAF1 and *Cfio* CC1, suggesting porosity similar to that of the appressorial cell wall of *Corb* 104-T; however, they generated high turgor pressure in a melanisation-independent manner (Supplementary Fig. 7b, c).” (Line 194).

References

<u>Deleted:</u>

51. Kumakura, N. et al. Dihydroxyhexanoic acid biosynthesis controls turgor in pathogenic fungi. *bioRxiv* 606736, Preprint at https://doi.org/10.1101/2024.08.07.606736 (2024).

<u>Added:</u>

15. Kumakura, N. et al. Dihydroxyhexanoic acid biosynthesis controls turgor in pathogenic fungi. *Science* **391**, 700–706 (2026).
60. Müller, T. et al. Plant infection by the necrotrophic fungus Botrytis requires actin-dependent generation of high invasive turgor pressure. *New Phytol.* **244**, 192–201 (2024).

//Reviewer 3 comment:

3. For the HTE strains, the precise infection process is undescribed. How do hyphal tips overcome plant barriers without a specialized appressorium? Ultrastructural or cytological evidence is lacking.

//Author reply:

In the pioneer work on the finding of HTE in *Colletotrichum* (Hiruma et al., 2010), the authors had already characterised how appressorium-independent hyphal tips of the *Colletotrichum* overcome plant barriers; Hiruma et al. demonstrated that the infection process of HTE shared entry mode with MAE through penetration peg-like structures without appressoria by the staining of papillary callose deposition, a defence response marker underneath the fungal penetration pegs, in addition to microscopic inspection (Hiruma et al., 2010). However, as the reviewer pointed out, there was no particular description about the infection process of HTE in our original manuscript. Therefore, we referred to the characteristics of *Colletotrichum* HTE infection process described in the previous report (Hiruma et al., 2010), and demonstrated that our HTE-type strain also penetrated plant surface through narrow peg-like structures by additional papilla staining experiment (New Supplementary Fig. 12). The additional sentences in the result section are as follows:

"Notably, a pioneering study has characterised the infection process of HTE as a shared entry with MAE through penetration peg-like structures, excluding appressorium formation, based on staining of papillary callose deposition, a defence response marker beneath the fungal penetration pegs, and microscopic observation[34]. Consistently, we confirmed pathogen-induced papilla beneath successful fungal entry sites, where callose deposition was excluded from the centre of papillae, due to fungal penetration (Supplementary Fig. 12)." (Lines 339–345).

Reference

34. Hiruma, K. et al. Entry mode-dependent function of an indole glucosinolate pathway in *Arabidopsis* for nonhost resistance against anthracnose pathogens. *Plant Cell* **22**, 2429–2443 (2010).

//Reviewer 3 comment:

4. While the study excellently characterizes NMAE in vitro or under controlled conditions (appressoria are formed on the immunocompromised A. thaliana), the agronomic significance and real-world impact remain unclear. Do NMAE and HTE strains cause disease of comparable severity and spread in field conditions? This is critical for assessing the practical implication for disease management.

//Author reply:

As the reviewer pointed out, our data were derived from controlled laboratory inoculation assays, not field studies. As the other reviewer also suggested, to prevent misleading of the readers, we deleted the phrase "in nature" and used "spontaneous NMAE" in the sentences with regards to fungal inoculation assays. We also deleted or changed "natural conditions" to "MBI-free conditions". But

on the other side of that, in the original manuscript, we performed inoculation assays of MAE- and NMAE-type *Colletotrichum* fungi on many host and lesion-producing plants in addition to the immunocompromised mutants of the nonhost model plant *A. thaliana*: *A. thaliana* (*Chig* Abr1-5), cucumber (*Corb* 104-T), Apple (*Csia* MAF1), cosmos (*Cfio* CC1), Japanese flowering cherry (*Cnym* PL1-1-b), hyacinth (*Cfio* HF3), Japanese ivy (four *Ctric* strains), spinach (*Cfio* CC1), cockscomb (*Cfio* CC1), maize (*Cfio* CC1), sorghum (*Cfio* CC1), and rice (*Cfio* CC1) (Figs. 1, 2, 5, Supplementary Figs 9, and original Supplemental Fig. 13 (New Supplemental Fig. 14)). Of those, we had already characterised the NMAE on cosmos (*Cfio* CC1), Japanese flowering cherry (*Cnym* PL1-1-b), hyacinth (*Cfio* HF3), and rice (*Cfio* CC1) (Figs. 1, 2, 5 and original Supplemental Fig. 13 (New Supplemental Fig. 14)) in addition to the *Arabidopsis* mutants. Furthermore, we performed additional inoculation assays of NMAE-type pathogens on non-model plants in the revised manuscript: sacred bamboo (*Cfio* CC1), Japanese andromeda (*Cfio* CC1), and Japanese summer orange (*Cfio* S96a1 and *Cgod* MC1) (New Supplementary Fig. 15). The HTE was originally characterised on the mutants of model plant *A. thaliana* (Hiruma et al., 2010), and we also identified most HTE-type strains on *A. thaliana* mutants (Fig. 7, 8), except for HTE-based host invasion by *Cfio* HF3 (Fig. 5g, h) in the original manuscript. Therefore, we additionally characterised HTE of *Cmus* Noudai-Netsu-Saku-Hogo-28(3) and *Ctro* Ishigaki-Banana2 on their host plants (New Figs. 7, 8). These indirect evidences support the NMAE- and HTE-based fungal invasion and subsequent infection of *Colletotrichum* fungi in the field. However, as the other reviewer also suggested, we reframed the style of writing to avoid overstating regarding to the field relevance, because the real-world severity and the spread are currently unknown, as you pointed out.

We added the new results and the following sentences in the manuscript:
"Notably, the pathogenicity of *Cmus* Noudai-Netsu-Saku-Hogo-28(3) on host banana was attributed to HTE-based invasion, as this pathogen scarcely formed appressoria on the host surface but exhibited HTE, which is induced by sugar and/or other ripe fruit-derived signal(s) (Fig. 7d, e)." (Lines 356–360).
"On the contrary, the virulence of *Ctro* Ishigaki-Banana2 on ripe fruits of its host banana was not affected by treatments with any of the investigated MBIs (Fig. 8f). *Ctro* Ishigaki-Banana2 consistently exhibited HTE-type morphogenesis on the host surface regardless of the presence or absence of MBIs, suggesting host infection via HTE induced by sugar and/or other ripe fruit-derived signal(s) (Fig. 8g)." (Lines 416–421).

We also deleted the sentence "More importantly, in paddy field under agrochemical disease control using MBIs, rice may remain susceptible to NMAE-based invasion." (Line 393).

We substantially toned down the expression and revised the sentences as follows:

"In contrast to MBI-D[31–33], *P. oryzae* strains resistant to MBI-R fungicides have not emerged, despite several decades of continuous use[57]. Therefore, the potential risk for resistance development to MBI-R fungicides is estimated to be low. Intriguingly, we found that *Ctro* Ishigaki-Banana2 exhibited specific insensitiveness to MBI-R pyroquilon in the lesion production on *A. thaliana* immunocompromised mutants, whereas other MBIs, such as carpropamid and tolprocarb, remained effective (Fig. 8a). Another MBI-R, fthalide, strongly prevented the *Ctro* Ishigaki-Banana2-induced lesion formation in *A. thaliana*, in contrast with the dose-dependent lesion-producing effects of pyroquilon (Fig. 8b). Specifically, in the presence of carpropamid or tolprocarb, appressoria formed on the plant surface were arrested at the premelanisation stage and could not penetrate plant epidermis (Fig. 8c). Therefore, *Ctro* Ishigaki-Banana2 was classified as a conventional MAE-type pathogen (Fig. 4b). In contrast, *Ctro* Ishigaki-Banana2 unexpectedly exhibited suppressed appressorial formation, germ tube-like hyphal extension and HTE-based invasive hyphal development in the presence of pyroquilon, while melanisation of partly formed appressoria was sufficiently prevented (Fig. 8c, d). Therefore, *Ctro* Ishigaki-Banana2 was characterised as pyroquilon-induced HTE-type (Fig. 4b). Moreover, this pathogen was classified as a putative sugar-induced HTE-type strain (Fig. 8e). These phenomena indicate that, in addition to carbohydrates, specific agrochemicals may act as factors that induce appressorium-independent, HTE-related morphogenesis and the subsequent plant invasion via HTE by specific *Colletotrichum* strains, although MBIs are not applied to crops affected by *Colletotrichum*. On the contrary, the virulence of *Ctro* Ishigaki-Banana2 on ripe fruits of its host banana was not affected by treatments with any of the investigated MBIs (Fig. 8f). *Ctro* Ishigaki-Banana2 consistently exhibited HTE-type morphogenesis on the host surface regardless of the presence or absence of MBIs, suggesting host infection via HTE induced by sugar and/or other ripe fruit-derived signal(s) (Fig. 8g)." (Lines 396–421).

"However, the progression of faint lesions was restricted and assumed to be a hypersensitive-like response in rice (Supplementary Fig. 14e), which was consistent with the well-known incompatibility of *Colletotrichum* fungi with rice. Therefore, at present, it is unlikely that NMAE-type and polyphagous *Colletotrichum* strains in the acutatum SC will become fully adapted to rice and pose a serious threat." (Lines 543–548).

Reference

34. Hiruma, K. et al. Entry mode-dependent function of an indole glucosinolate pathway in *Arabidopsis* for nonhost resistance against anthracnose pathogens. *Plant Cell* **22**, 2429–2443 (2010).

//Reviewer 3 comment:

5. The gene knockout mutants in Colletotrichum used in this study lack southern blotting validation and complementation assays.

//Author reply:

As the reviewer pointed out, gene complementation assay is usually necessary to confirm the restoration of the lost phenotypic function in gene knockout experiments by eliminating the possibility of ectopic integration of the transforming DNA. However, in the flow of this study, we had already confirmed sufficient melanisation-preventive effects of MBIs on appressoria of *Colletotrichum* in advance (Fig. 1 and Supplementary Fig. 3). The melanin-deficient (Δ*scd1*) mutants were used for additional verification; the same results of *Colletotrichum* Δ*scd1* mutants strongly supported the MBI-treatment experiments (Fig. 2, 3, Supplementary Fig. 8, and New Supplementary Fig.14). The generated lines of Δ*scd1* mutants in each *Colletotrichum* strain exhibited the complete loss of appressorial melanisation (New Supplementary Fig. 4), the same as previously reported Δ*scd1* mutants of other *Colletotrichum* strains. All our Δ*scd1* mutants, except for NMAE-type *Cfio* CC1 Δ*scd1*, also lost the plant penetration ability, which is also consistent with the long-standing theory in plant pathology (Fig. 2 and Supplementary Fig. 3). These coherent set of data with genomic PCR analyses indicate that there is no doubt as to *SCD1* gene disruption, not ectopic transformants. In the MBI treatment assays, the wild-type *Colletotrichum* strains, which has no gene manipulation, substantially had the competence for the complemented strains in a sense. Furthermore, NMAE-type *Cfio* CC1, the key strain in this study, did not exhibit the loss of function regarding to the plant penetration and lesion-producing abilities in both MBI treated and *SCD1* disrupted conditions (Fig. 1 and 2), causing failure of complementation experiment. In addition, even low melanisation mutants still sufficiently lost the plant penetration ability and consequent pathogenicity in previous reports (Lin et al., 2012; Harata and Okuno, 2019). The purpose of generation of the Δ*scd1* mutants in this study is to strengthen the results in MBI treatment experiments, not to characterise the function of already well-known gene *SCD1* itself. We successfully observed nonmelanisation phenotype of appressoria in two lines Δ*scd1* of each *Colletotrichum* strain (New Supplementary Fig. 4) by convention. For these reasons, we think it is not necessarily essential to perform additional complementation assays in our study. As expected, in the genomic PCR analyses, we clearly confirmed *SCD1* gene disruption by the homologous recombination (New Supplementary Fig. 4b). If ectopic integration occurs, we can simultaneously detect the DNA fragments of *SCD1* and hygromycin resistance gene (*HPH*), and easily notice the melanised appressoria of that strain. We also think that the additional southern blot validation is not necessarily essential. Furthermore, many previous report performed PCR analyses but did not additional southern blot analyses for the validation of specific gene disruption in *Colletotrichum*

fungi (Fujihara et al., 2010; Irieda et al., 2014; Dubey et al., 2016; Nakamura et al., 2019; Wang et al., 2021; Zhang et al., 2021; Irieda and Takano, 2021; Kodama et al., 2022; Yamada et al., 2023; Inoue et al., 2023; Hiruma et al., 2023; Tamburrini et al., 2024; Zhang et al., 2025; Kumakura et al., 2026, etc.). On the other hand, we reflect on our insufficient explanation and complicated research flow which led to what the reviewer pointed out, and added the revision as follows.

With regards to strains *Csia* MAF1 and *Cfio* CC1, the melanin-deficient mutants of which are the first appearance in a *Colletotrichum* research, we fortunately obtained ectopic transformants, therefore performed additional genomic PCR analyses and added the data of amplified DNA patterns of the ectopic transformants for comparison to successful disruption mutants (New Supplementary Fig. 4b).

Based on the sufficient preventive effects of MBIs in our study, to avoid undervaluation of the MBI treatment experiments, we deleted the sentence "However, the MBIs may have lacked persistent and effective activity." (Lines 144).

We also revised the sentence as follows: "To further verify the NMAE ability of *Cfio* CC1, we generated melanin-deficient (Δ*scd1*) mutants of the four *Colletotrichum* strains, all of which formed unicellular, morphologically mature, nonmelanised appressoria (Supplementary Fig. 4)." (Lines 147–149).

We also added the sentence "Except for *Cfio* CC1, these typical phenotypes of MBI-treated wild-type (Fig. 1) or melanin-deficient mutant (Fig. 2 and Supplementary Fig. 4) *Colletotrichum* strains were consistent with the long-standing dogma that melanisation was universally essential for plant invasion[5,6]." in the revised manuscript (Lines 155–158).

References

Lin, S. Y., Okuda, S., Ikeda, K., Okuno, T. & Takano, Y. *LAC2* encoding a secreted laccase is involved in appressorial melanization and conidial pigmentation in *Colletotrichum orbiculare*. *Mol. Plant Microbe Interact.* **25**, 1552–1561 (2012).

48. Harata, K. & Okuno, T. Threonine synthase *CoTHR4* is involved in infection-related morphogenesis during the pre-penetration stage in *Colletotrichum orbiculare*. *Microb. Pathog.* **137**, 103746 (2019).

52. Fujihara, N. et al. Peroxisome biogenesis factor PEX13 is required for appressorium-mediated plant infection by the anthracnose fungus *Colletotrichum orbiculare*. *Mol. Plant Microbe Interact.* **23**, 436–445 (2010).

Irieda, H. et al. *Colletotrichum orbiculare* secretes virulence effectors to a biotrophic interface at the primary hyphal neck via exocytosis coupled with SEC22-mediated traffic. *Plant Cell* **26**, 2265–

2281 (2014).

Dubey, A. K. et al. Cation-stress-responsive transcription factors SltA and CrzA regulate morphogenetic processes and pathogenicity of *Colletotrichum gloeosporioides*. *PloS One* **11**, e0168561 (2016).

Nakamura, M., Okamura, Y. & Iwai, H. Plasmid-based and -free methods using CRISPR/Cas9 system for replacement of targeted genes in *Colletotrichum sansevieriae*. *Sci. Rep.* **9**, 18947 (2019).

35. Wang, X., Lu, D. & Tian, C. Analysis of melanin biosynthesis in the plant pathogenic fungus *Colletotrichum gloeosporioides*. *Fungal Biol.* **125**, 679–692 (2021).

Zhang, R. et al. Fungal effector SIB1 of *Colletotrichum orbiculare* has unique structural features and can suppress plant immunity in *Nicotiana benthamiana*. *J. Biol. Chem.* **297**, 101370 (2021).

36. Irieda, H. & Takano, Y. Epidermal chloroplasts are defense-related motile organelles equipped with plant immune components. *Nat. Commun.* **12**, 2739 (2021).

51. Kodama, S., Kajikawa, N., Fukada, F. & Kubo, Y. Niemann-Pick type C proteins are required for sterol transport and appressorium-mediated plant penetration of *Colletotrichum orbiculare*. *mBio* **13**, e0223622 (2022).

Yamada, K., Yamamoto, T., Uwasa, K., Osakabe, K. & Takano, Y. The establishment of multiple knockout mutants of *Colletotrichum orbicular* by CRISPR-Cas9 and Cre-*loxP* systems. *Fungal. Genet. Biol.* **165**, 103777 (2005).

Inoue, Y. et al. Selective deployment of virulence effectors correlates with host specificity in a fungal plant pathogen. *New Phytol.* **238**, 1578–1592 (2023).

Hiruma, K. et al. A fungal sesquiterpene biosynthesis gene cluster critical for mutualist-pathogen transition in *Colletotrichum tofieldiae*. *Nat. Commun.* **14**, 5288 (2023).

Tamburrini, K. C., et al. The disordered C-terminal tail of fungal LPMOs from phytopathogens mediates protein dimerization and impacts plant penetration. *Proc. Natl. Acad. Sci. USA* **121**, e2319998121 (2024).

Zhang, R. et al. Two homologous Zn_2Cys_6 transcription factors play crucial roles in host specificity of *Colletotrichum orbiculare* by controlling the expression of cucurbit-specific virulence effectors. *New Phytol.* **246**, 237–250 (2025).

15. Kumakura, N. et al. Dihydroxyhexanoic acid biosynthesis controls turgor in pathogenic fungi. *Science* **391**, 700–706 (2026).

60. Müller, T. et al. Plant infection by the necrotrophic fungus Botrytis requires actin-dependent generation of high invasive turgor pressure. *New Phytol.* **244**, 192–201 (2024).

Finally, we reiterate our thanks to Reviewer 3 for improving our manuscript.

Responses to reviewer 1:

//Reviewer 1 comment:

Reviewer #1 (Remarks to the Author):

The authors have carefully and thoroughly addressed all of the reviewer's comments. The revisions have significantly improved the clarity, rigor, and overall quality of the manuscript. In particular, the conceptual framing, terminology, and interpretation have been appropriately refined in line with the reviewer's suggestions.

I am satisfied with the authors' responses and consider that the revised manuscript is now suitable for publication in its current form.

//**Author reply:**

We reiterate our thanks to Reviewer 1 for improving our manuscript.

Responses to reviewer 2:

//Reviewer 2 comment:

Reviewer #2 (Remarks to the Author):

The authors have responded adequately to each of the concerns/suggestions and made changes to reflect their responses in the manuscript itself.The manuscript is know acceptable for publication.

//**Author reply:**

We reiterate our thanks to Reviewer 2 for improving our manuscript.

Responses to reviewer 3:

//Reviewer 3 comment:

Reviewer #3 (Remarks to the Author):

The authors have addressed the reviewers' comments and revised the manuscript accordingly. I have no additional concerns.

//Author reply:

We reiterate our thanks to Reviewer 3 for improving our manuscript.
